# Electroencephalography signatures of motor error and stimulus-driven attention in electrical muscle stimulation-induced wrist movements under motor imagery

**DOI:** 10.3389/fnhum.2025.1713908

**Published:** 2026-01-27

**Authors:** Kento Suemitsu, Isao Nambu

**Affiliations:** Graduate School of Engineering, Nagaoka University of Technology, Nagaoka, Japan

**Keywords:** electrical muscle stimulation, electroencephalography, event-related potentials, motor error, motor imagery, stimulus-driven attention

## Abstract

This study examines how electroencephalography (EEG) signatures are modulated during passive movements induced by electrical muscle stimulation (EMS). Specifically, we focused on three key factors: (1) motor errors, (2) stimulus-driven attention, and (3) cognitive conditions (motor imagery and waiting). We tested how error-related neural responses are modulated by the mismatch between predictions and actual sensory inputs during motor imagery. To this end, we introduced a wrist-movement paradigm combining motor imagery with EMS. Participants either imagined wrist dorsiflexion toward visual targets or passively waited. EMS was then applied to induce passive wrist movements. EMS intensity and presentation rate were manipulated so that the magnitude of stepwise motor error (physical deviation from the target) and stimulation-driven attention varied inversely, allowing us to evaluate their respective neural effects. Event-related potential (ERP) components at approximately 700 ms reflected graded evaluation of motor error. An ERP component at approximately 300 ms was modulated primarily by EMS intensity and presentation rate, consistent with stimulus-driven attention. Mu-band suppression reflected the match between predicted and actual sensory inputs. Theta-band enhancement in the absence of motor imagery suggested increased sensory unpredictability due to the lack of internally generated predictions, whereas beta-band activity may have been related to fluctuations in state prediction. To our knowledge, the proposed motor imagery with the EMS wrist-movement paradigm is the first to investigate how (1) motor errors, (2) stimulus-driven attention, and (3) cognitive conditions shape EEG signatures during passive, electrically induced movements. Understanding how these neural signals vary with mismatch between predictions and actual sensory inputs offers valuable insights into the mechanisms of error processing and sensory prediction in the human brain.

## 1 Introduction

Our brains continuously generate predictions about sensory inputs from the external environment and compare them with actual inputs to detect prediction errors, enabling adaptive adjustments in behavior. In motor control, the detection of such errors is regarded as a fundamental cognitive function essential for flexible movement (Kawato and Gomi, 1992). Electroencephalography (EEG) has been widely employed as a non-invasive technique (Chavarriaga et al., 2014; Iwane et al., 2023) to detect neural responses associated with error detection in humans, namely event-related potentials (ERPs) that is associated with error processing including the error-related negativity (ERN), a rapid fronto-central response linked to early error detection (Iwane et al., 2023; Vocat et al., 2011), and error positivity (Pe), a later parietal response that is associated with subsequent evaluative processes (Boldt and Yeung, 2015; Steinhauser and Yeung, 2010; Ridderinkhof et al., 2009; Overbeek et al., 2005; Nieuwenhuis et al., 2001). These responses are collectively referred to as error-related potentials (ErrPs). On the other hand, unpredicted external events can also deviate sensory predictions and trigger responses of stimulus-driven attention. Stimulus-driven attention refers to the automatic orienting of attention toward unpredicted or salient external events. These responses are commonly reflected in the fronto-central P3a and parietal P3b components, indexing rapid detection of unpredicted stimuli and subsequent contextual updating (Wessel, 2018; Polich, 2007).

Notably, early Pe and P3a have been proposed to reflect partially overlapping early-detection processes, and this idea aligns with the Adaptive Orienting Theory, a unified framework linking error processing with orienting responses to unpredicted external events (Wessel, 2018). However, whether early Pe and P3a reflect a common process remains unclear.

Besides unpredicted external events, mismatch between internally generated predictions and incoming sensory feedback can also lead to prediction errors. Even in the absence of voluntary movement, motor imagery engages internal forward models that generate predicted sensory consequences based on action intention (Jeannerod, 2001; Wolpert et al., 1995; Grush, 2004; Kilteni et al., 2018). When these internally generated predictions are compared with sensory feedback, mismatches can give rise to prediction-error responses, a process often accompanied by modulation of the sensorimotor mu rhythm (Kilteni et al., 2018; Shibuya et al., 2018; Evans and Blanke, 2013). In addition, error-related neural activity is known to involve oscillatory responses in fronto-central theta, beta, and gamma bands (Iwane et al., 2023; Moreau et al., 2023; Cavanagh and Frank, 2014; Omedes et al., 2015; Jahani et al., 2020; Griffin et al., 2025; Völker et al., 2018). However, how such internal predictions interact with externally triggered mismatch, particularly during passive movements induced by external stimulation, is not clearly understood.

**Figure 1:**
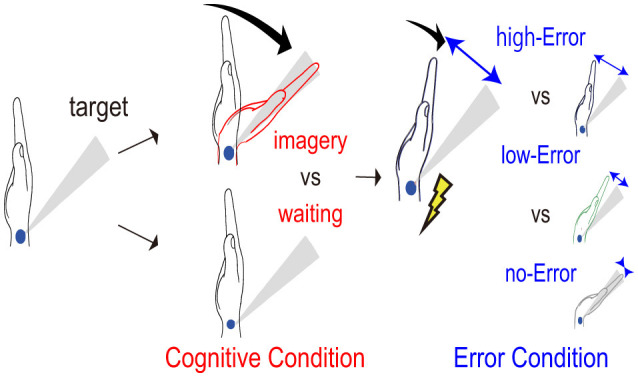
Schematic diagram of the experimental paradigm. Participants performed the experiment under two cognitive conditions: motor imagery of wrist dorsiflexion toward a target or waiting without movement. During these conditions, wrist dorsiflexion was induced by electrical muscle stimulation, and error conditions relative to three targets were presented.

To address these issues, we applied electrical muscle stimulation (EMS) during motor imagery of wrist dorsiflexion. As illustrated in Figure 1, EMS produces passive movements with aligned visual and somatosensory feedback and can partially preserve the sense of agency even under external control (Tajima et al., 2022; Veillette et al., 2023). This paradigm allowed us to examine neural responses arising from mismatches between internally simulated movements and EMS-induced passive movements as well as from effects of attention driven by unpredicted stimulus. In addition, this paradigm was designed such that motor error and stimulus-driven attention varied simultaneously within the same trial, while their magnitudes of mismatch were manipulated in opposite directions, enabling their neural contributions to be analytically separated. Accordingly, if prediction error responses and externally induced attention responses rely on a common mechanism for handling unpredictable events, the EEG signature should increase in response to the magnitude of both types of mismatch.

Using this experimental paradigm, this study examines how motor error, stimulus-driven attention, and motor imagery modulate EEG signatures to EMS-induced passive movements. In this context, motor error denotes the physical deviation from the target. Motor imagery engages internal forward models that generate predicted sensory inputs, thereby making prediction errors more likely to arise from mismatches between predicted and actual sensory inputs. Based on prior work on prediction errors, stimulus-driven attention, and motor imagery, we formulated the following hypotheses:

(H1) Large mismatches between predicted and actual sensory inputs enhance error-related neural responses, whereas low-frequency or high-intensity EMS pulses increase P3a/P3b amplitudes through heightened stimulus-driven attention.
(H2) If prediction error and stimulus-driven attention responses share a common detection mechanism, EEG signatures should show similar responses even when error and attention components—though they vary together within a trial—are examined separately.
(H3) Motor imagery interacts with the magnitude of motor error such that the neural responses will be weaker than those in the waiting condition for small deviations but stronger for large deviations.

## 2 Related work

Error and stimulus-driven attention responses have been jointly explained within the Adaptive Orienting Theory (Wessel, 2018), which proposes that both reflect rapid detection of unpredicted events. On the other hand, internal forward-model theories characterize motor control as predicting the sensory inputs of actions and comparing them with actual sensory inputs, with the resulting mismatches interpreted as prediction errors (Jeannerod, 2001; Wolpert et al., 1995; Grush, 2004). However, the current understanding of how error processing, stimulus-driven attention, and motor imagery interact remains fragmented. To establish the theoretical basis for the present study, we reviewed four major lines of research and highlight unresolved issues that motivated our EMS-based paradigm.

Research on ErrPs has primarily focused on errors arising during voluntary movements, such as button presses or reaching movements. Prior studies have shown that the ERN reflects early error detection and is primarily generated in the anterior cingulate cortex (Ullsperger and von Cramon, 2001), whereas the Pe indexes later evaluative and awareness-related processes with early fronto-central and late parietal–occipital sources (Overbeek et al., 2005; Nieuwenhuis et al., 2001). ErrP components have been shown to vary parametrically with the magnitude of visuomotor mismatches during voluntary movements (Iwane et al., 2023; Vocat et al., 2011), indicating that these components are sensitive to stepwise deviations. Classic findings further show that ERN occurs even when errors go unnoticed whereas the Pe selectively tracks error awareness (Nieuwenhuis et al., 2001), that the two components have been suggested to be functionally distinguishable (Overbeek et al., 2005), and that late Pe resembles a P3b-like evaluative updating process (Ridderinkhof et al., 2009). Other work has shown that the Pe scales continuously with decision confidence (Boldt and Yeung, 2015). Together, these studies support the view that ErrPs reflect partly distinct stages of error processing, with the ERN indexing early error detection and Pe reflecting later evaluation or awareness of errors. However, these mechanisms have been examined during voluntary actions, and whether they are applicable to mismatches between predicted and actual sensory inputs during passive movements, particularly in situations where such mismatches vary across stepwise deviations, is still unclear.

A parallel literature has examined stimulus-driven attention, showing that unpredicted or deviant events elicit rapid, automatic attentional shifts reflected in the P3a and P3b components (Wessel, 2018; Polich, 2007). Sudden somatosensory events can elicit attentional responses. Novel tactile stimuli, in particular, have been shown to evoke orienting-related P3 activity (Yamaguchi and Knight, 1991), and deviant or unpredicted tactile inputs can engage early detection mechanisms (Spackman et al., 2007). According to the Adaptive Orienting Theory (Wessel, 2018), any unpredicted event automatically triggers stimulus-driven attentional orienting toward the unpredicted input. This orienting response is considered to reflect an early evaluation of mismatches between expected and incoming sensory events and is expressed across both stimulus-driven attention and error-related components, which share partially overlapping neural processes. Studies that have independently manipulated errors and unpredicted events further support this view. For example, Holroyd et al. (2009) demonstrated that ERN-like activity can be elicited even in the absence of overt errors when outcomes are unpredicted. Wessel et al. reported that neural responses to errors and unpredicted events share a common neural mechanism (Wessel et al., 2012). Together, these findings indicate that error processing and stimulus-driven attention share an early detection architecture yet diverge at later evaluative stages. However, how error-related and stimulus-driven attentional responses behave when both processes change in stepwise levels within the same task is unclear.

Internal-model theories propose that the motor system continuously predicts the sensory consequences of actions, using forward models to map motor commands onto expected sensory feedback (Wolpert et al., 1995; Jeannerod, 2001; Grush, 2004). Motor imagery has been argued to recruit the same simulation machinery as overt action, generating internal predictions even in the absence of movement (Jeannerod, 2001; Grush, 2004), and previous studies provide direct evidence that motor imagery involves predicting the sensory inputs of the imagined movement (Kilteni et al., 2018). Consistent with this, mu-rhythm modulation has been linked to these predictive processes and to the integration of body ownership and motor imagery, suggesting that sensorimotor oscillations index the comparison between predicted and actual sensory states (Shibuya et al., 2018; Evans and Blanke, 2013). Previous work on the sense of agency emphasizes that perceived control depends on the alignment between action intention and sensory inputs (Haggard, 2017). Extending this framework to externally driven movements, recent EMS studies have shown that when visual and proprioceptive feedback are aligned, externally induced muscle contractions can still elicit a partial sense of agency over the resulting movement (Tajima et al., 2022; Veillette et al., 2023). Despite these advances, very few studies have examined how internal predictions generated during motor imagery interact with EMS-induced passive movements to produce mismatches between predicted and actual sensory inputs or how such mismatches shape error-related neural responses under conditions of partially preserved agency.

Error-related neural activity is expressed in fronto-central theta oscillations. Across visuomotor tasks, theta power increases whenever predicted sensory inputs deviate from actual feedback, such as during visuomotor rotation paradigms where cursor feedback deviates from the intended movement (Iwane et al., 2023) or during gradually accumulating trajectory mismatches in continuous monitoring tasks (Omedes et al., 2015). Beyond one's own movements, theta also increases when observing unpredicted deviations in another agent's actions (Moreau et al., 2023), suggesting that the system tracks deviation of prediction in both self-generated and externally generated motor events. Complementing these findings, frontal-midline theta increases during cognitive conflict and negative feedback (Cavanagh and Frank, 2014), indicating that theta reflects a general computation for detecting deviations from predicted states. Taken together, theta appears to signal unpredicted events across self-generated actions and observed movements.

Beta-band activity is typically interpreted as signaling the maintenance of current sensorimotor or cognitive states. Beta power decreases when a change from the current state is expected (Engel and Fries, 2010). Beyond this general status quo function, spatially distinct beta modulations have been shown to track implicit adaptation and explicit re-aiming during visuomotor learning (Jahani et al., 2020), and beta bursts can oppose learning-related reactivation processes in the motor cortex (Griffin et al., 2025). These results suggest that beta suppression broadly indexes the shift away from the currently predicted or stabilized state. High-gamma activity increases with visuomotor errors (Iwane et al., 2023) and during erroneous outcomes in both noninvasive and intracranial EEG (Völker et al., 2018), reflecting localized neural processing triggered by unpredicted sensory events.

Taken together, prior work has advanced our understanding of error processing, deviance detection, and predictive motor control, yet several key gaps remain. Specifically

1. how neural responses associated with motor error and stimulus-driven attention change during passive movements, particularly when both factors vary in stepwise levels within the same paradigm is unclear;
2. stimulus-driven attention and error processing have usually been treated as separate factors, resulting in knowledge gaps regarding how unpredicted stimulus (e.g., EMS intensity and presentation rate) modulate P3a/P3b amplitudes when both processes occur within the same trials; and
3. little is known about how internally generated predictions during motor imagery influence neural responses to passive, EMS-induced deviations, including whether imagery alters error-related ERP components or oscillatory signatures relative to waiting.

## 3 Materials and methods

### 3.1 Participants

Sixteen healthy participants (two females; mean age: 22.2 ± 2.2 years), who were students at Nagaoka University of Technology and had no skin abnormalities at the site of EMS, were recruited for the experiment. All participants were right-handed according to their Edinburgh handedness test scores (score range: 68.4%–100%). The experimental protocol was approved by the Ethics Committee of Nagaoka University of Technology (Number 20240902). All participants provided informed consent and agreed to undergo screening for skin abnormalities at the EMS site to ensure safety during EMS (screening details are listed in the Supplementary material).

### 3.2 Experimental design

The participants sat in a comfortable, adjustable-height chair and placed their right arm on a cushioned wooden platform positioned in front of a display (65Z570K, TVS REGZA, Kanagawa, Japan) that visualized the degree of wrist dorsiflexion. They were instructed to avoid large movements and excessive blinking. The forearm was secured to a custom wooden platform with bands, and the right hand was placed in wooden fixtures with acrylic plates to prevent finger flexion. A 0.75-mm polypropylene craft sheet (PS-3, Acrylic SUNDAY, Tokyo, Japan) was attached to each corner of the four acrylic plates on the wooden fixtures, enabling smooth wrist dorsiflexion. A wooden block on the palmar side prevented wrist flexion beyond the neutral position, where the hand and forearm were aligned.

The experiment was conducted over 2 days. Three task conditions were set: voluntary, imagery, and waiting. Each day, three sessions were conducted, with each session corresponding to one condition. This paradigm was designed to test how (1) motor errors, (2) stimulus-driven attention, and (3) cognitive conditions modulate EEG markers during passive, EMS-induced wrist movements. In the voluntary session, participants performed active wrist flexion. In the passive sessions (imagery and waiting), wrist dorsiflexion movement was induced by EMS. Each session comprised four blocks, totaling eight blocks for each condition across 2 days. The voluntary session was always conducted first to aid kinesthetic imagery. The order of imagery and waiting sessions was counterbalanced across participants and days.

**Figure 2:**
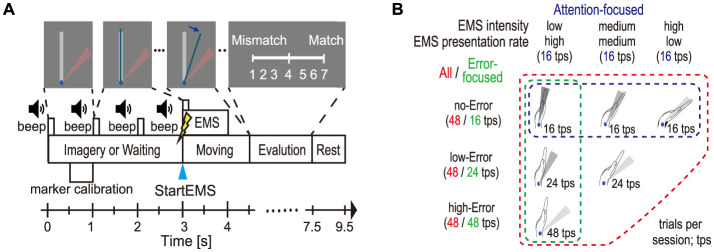
Overview of the experimental design. **(A)** Trial structure and timing in passive sessions(imagery and waiting), including a time-locked event: StartEMS (stim onset). The Evaluation segment was excluded from voluntary sessions. **(B)** Combinations of three target and EMS intensity levels (including presentation rate) in the passive sessions, with the set defined as All set and the subsets as Attention-focused (no-Error) and Error-focused (LowI/HighP) subsets. Trials in All set were evenly distributed across error conditions.

In the voluntary session, the participants moved a blue bar (representing wrist angle) toward a red fan-shaped target displayed on the screen (Figure 2A). Corrective movements were not allowed. Each trial consisted of a 3-s ready period, 1.5-s task period, 3-s evaluation period, and 2-s rest period. In the passive sessions, the evaluation period was omitted. During the ready period, a beep sounded once per second (at 0, 1, 2, and 3 s). The target appeared at the first beep, and wrist angle calibration was performed between 0.5 and 1.0 s. At the final beep (3 s), participants either initiated movement (voluntary session) or received EMS (passive sessions). In the passive imagery session, participants repeatedly engaged in kinesthetic motor imagery of wrist flexion toward the visual target. As soon as the target appeared at the first beep, they began to imagine the sensory experience of the wrist moving toward the target and maintained this kinesthetic imagery throughout the ready period. They were further encouraged to anticipate the final beep at 3 s and to internally generate the same kinesthetic motor command that would initiate the imagined movement at that moment. This procedure ensured continuous engagement of forward-model-based motor prediction. In contrast, in the waiting session, participants passively awaited the EMS without generating any motor intention. After the wrist flexion, in the imagery session, the participants rated the incongruence between their imagined kinesthetic sensation and the sensation induced by EMS using a 7-point scale (1 = mismatch, 7 = match) via keyboard input. In the waiting session, they rated how far the induced wrist movement deviated from the target. During the rest period, the participants were instructed to return to the initial position for calibration.

Three targets (near-target: 7° ± 6°, medium-target: 20° ± 6°, far-target: 33° ± 6°) were presented (Figure 2B, positioned along three diagonal lines toward the lower left). In the voluntary session, trials were repeated if the movement exhibited multiple peaks or failed to reach the target within 1 s. In the passive sessions (imagery and waiting), wrist dorsiflexion was induced by EMS (low-intensity, medium-intensity, high-intensity) with a 300-μs pulse width, 20-Hz frequency, and 1-s duration. The voltage amplitude was individually adjusted prior to the experiment to achieve each EMS intensity. During the imagery session, the participants were instructed to vividly imagine the sensation of wrist dorsiflexion toward the visual target from a first-person perspective. In the waiting session, they passively awaited EMS regardless of the target.

As shown in Figure 2B, the error between the visually presented target and the induced arrival point was categorized into three error conditions (no-Error, low-Error, and high-Error). The no-Error condition referred to physical congruence between the target and movement, while high-Error condition indicated the greatest incongruence. Each error condition included an equal number of trials (24 per day). Combinations of EMS intensity levels and target positions were pseudorandomly presented within each block. Thus, each error condition consisted of a total of 48 trials across the two experimental days (Figure 2B). In addition to error condition, stimulus-driven attention was manipulated through EMS intensity and presentation rate, inspired by oddball paradigms. High-EMS intensity (high-intensity and low-presentation rate) was predicted to elicit high attention, resembling the target stimuli in oddball paradigms; medium EMS intensity was anticipated to induce medium attention; and low-EMS intensity with a high-presentation rate was expected to evoke only low attention. Stimulus patterns in which the EMS-induced movement exceeded the target were excluded since they produced a situation where the error was large when stimulus-driven attention was low.

To clarify how stimulus-driven attention and motor error influence neural markers, we systematically adjusted three parameters—EMS intensity, presentation rate, and error level—each across stepwise levels (Figure 2B). The presentation rate across EMS conditions was designed to be approximately linear (based on Shannon information), and *post-hoc* analysis confirmed that EMS intensity levels were also approximately linear (see Supplementary Figure S1). In addition to analyzing all combinations of EMS intensity (including presentation rate) and error condition, we defined two targeted trial subsets to separately assess the contributions of error and stimulus-driven attention (Figure 2B).

### 3.3 Data acquisition

Visual stimulus presentation, input processing, EMS, and synchronization trigger generation were controlled using PsychoPy (Version 2024.2.1). Trigger signals generated by PsychoPy, positional data from infrared sensors, and EEG/electrooculogram (EOG) recordings were synchronized using LabStreamingLayer (library version 116).

#### 3.3.1 EMS protocol

EMS was delivered via a function generator (SEN-8203MG, Miyuki Giken, Tokyo, Japan) and an isolator (SS203JMG, Miyuki Giken, Tokyo, Japan) connected to disposable electrodes (F-150M, Nihon Kohden Corporation, Tokyo, Japan). Electrodes were placed over the right extensor carpi radialis longus muscle. A trigger signal was sent to the EXT TRIG input of the function generator via an NI-DAQ device (USB-6009, National Instruments Corporation, Austin, TX, USA). EMS intensity was controlled by adjusting the voltage input to the modulation (MODU) port of the function generator. The isolator was operated using a voltage-controlled method. For four participants, the EMS intensity was adjusted only at the beginning of the first passive session. However, owing to decreased movement caused by fatigue, amplitude adjustment was also performed for 12 participants before the second passive session. To enable comparison between sessions, the maximum current delivered in each session was recorded for these 12 participants.

The maximum current delivered to the muscle (*I*_max_) was calculated using a 10 μF capacitor *C* connected in series, based on the following formula:


$$\begin{array}{l} I_{\text{max}}=C\cdot V_{\text{peak}}/t_{\text{r}} \end{array}$$ (1)

*V*_peak_ was measured as the peak voltage across the capacitor using a digital oscilloscope (TDS2012, Tektronix, Inc., Beaverton, OR, USA). The rise time *t*_r_ was defined as the duration required for the voltage to increase from 10 to 90% of *V*_peak_. The resulting maximum current *I*_max_, calculated using the above formula, ranged from 6.2 to 19.0 mA.

#### 3.3.2 Behavioral data acquisition

An infrared marker was attached near the forearm along the dorsiflexion line formed by connecting the marker on the wrist and the one fixed near the fingertips. The positions of these three points were recorded at a sampling rate of 300 Hz, using a three-dimensional optical motion capture system (Optotrak Certus, Northern Digital Inc., Waterloo, ON, Canada). To standardize the initial position across trials, the acrylic plate attached to the hand fixture was placed against a wooden block. During the ready period (0.5–1.0 s), a calibration procedure was performed, and the average coordinates of the wrist and forearm markers were calculated and saved for each sample. To minimize the effect of skin deformation during wrist dorsiflexion, the wrist and forearm marker positions were treated as fixed within each trial. In real-time recording, only the marker on the fingertip was used. In passive sessions, the participants rated their experience following wrist flexion by entering a number from 1 to 7 using the keyboard.

#### 3.3.3 EEG and EOG acquisition

EEG and EOG signals were recorded using the Biosemi ActiveTwo system (ActiveTwo system, BioSemi B.V., Amsterdam, Netherlands) at a sampling rate of 512 Hz. EEG was measured with 64 active electrodes positioned according to the extended international 10–20 system. During acquisition, the common mode sense and driven right leg electrodes located within the EEG cap were used. For offline referencing, additional electrodes were placed on both earlobes. EOG signals were recorded using the external input (EXT) connections with additional electrodes. Horizontal EOG was measured using electrodes placed at the outer canthi of both eyes, while vertical EOG was recorded with electrodes positioned above the right eyebrow and below the right cheekbone.

### 3.4 Data analysis

The aim of this study was to examine how EEG signatures are modulated by three factors: (1) motor error (stepwise deviations between the target and EMS-induced movement), (2) stimulus-driven attention (manipulated via presentation rate and EMS intensity), and (3) motor imagery (imagery vs. waiting, reflecting the presence or absence of motor intention and internal simulation). To disentangle the effects of motor error and stimulus-driven attention, we analyzed the ERPs and EEG oscillations that were time-locked to the onset of EMS (StartEMS), using the All set (all combinations of error and EMS intensity and presentation rate) and two targeted subsets (Error-focused and Attention-focused subsets).

- All set: this set included all combinations of two factors, namely (1) motor error and (2) stimulus-driven attention (EMS intensity and presentation rate).
- Error-focused (LowI/HighP) subset: to minimize the influence of stimulus-driven attention by low-presentation rate and high-intensity stimuli and focus on neural responses to error-related information, EMS intensity was fixed at the lowest EMS intensity and high presentation rate (low-intensity/high-presentation). Trials were selected at three error conditions—no-Error, low-Error, and high-Error—yielding 16, 24, and 48 trials, respectively (Figure 2B).
- Attention-focused (no-Error) subset: to eliminate error-related information and focus on neural responses to the magnitude of stimulus-driven attention, we selected trials in which the passive movements induced by EMS reached the visual target (no-Error). These trials were grouped by EMS intensity and presentation rate, i.e., low-attention (low intensity/high presentation), medium-attention (medium intensity/medium presentation), and high-attention conditions (high intensity/low presentation), with 16 trials in each condition (Figure 2B).

#### 3.4.1 Behavioral data analysis

The acquired positional data were low-pass filtered using a fourth-order zero-phase Butterworth filter with a cutoff frequency of 10 Hz. Failed trial triggers were determined according to tangential velocity, calculated by numerical differentiation. In the cognitive conditions (imagery and waiting), transient artifacts were observed in some angular data. To correct for this, abrupt changes in the time series were detected and linearly interpolated. Specifically, fluctuations exceeding ±3° between consecutive samples were treated as outliers and replaced using linear interpolation between the nearest valid data points. For each EMS intensity level and participant, the time-series data of angles were averaged across trials and resampled to a unified time scale. Subsequently, the 95% confidence interval was estimated using the t-distribution based on the mean trajectory and standard error. After each task, the participants entered a rating from 1 to 7. If no response was entered within 3 s, the trial was excluded from the evaluation analysis. Each participant's ratings were averaged across trials within each of the six conditions: three error conditions (no-Error, low-Error, and high-Error) and two cognitive conditions (imagery and waiting). These averaged ratings were then compared across conditions. Trials in which the wrist failed to reach or overshot the experimenter-defined target range (±6°) during passive movement were excluded from both behavioral and EEG analyses.

#### 3.4.2 EEG analysis

EEG data analysis was performed using MNE-Python 1.8.0 (Gramfort et al., 2013) EEG and EOG signals were downsampled to 300 Hz using linear interpolation to match the sampling rate of the optical motion capture system, enabling precise temporal synchronization between EEG and the behavioral data. The 300-Hz rate was sufficient for analyzing data for frequencies below 90 Hz and improved computational efficiency. EEG signals were initially re-referenced to the average of the earlobe electrodes (A1 and A2). A band-pass filter (1–90 Hz) was applied to remove slow drifts, and a notch filter at 50 Hz was used to eliminate power line noise.

Artifacts were removed using independent component analysis (ICA) based on the InfoMax algorithm. The identified components were first classified using ICLabel (Pion-Tonachini et al., 2019; Li A. et al., 2022). The topographies, spectral profiles, and time courses of each component were then visually inspected following the ICLabel tutorial guidelines, and the recorded EOG channels were also used to assist in identifying ocular-related components. Components corresponding to ocular activity, muscle activity, power-line noise, eye blinks, and EMS-induced electrical artifacts were manually removed. EMS stimulation introduces frequency-specific-induced components (Tobimatsu et al., 1999). Here, 20-Hz stimulation produced induced activity at 20, 40, 60, and 80 Hz, which were removed during ICA cleaning. Supplementary Figures S2, S3 shows representative removed EMS-induced components, and another Supplementary material list all the rejected ICA components. Following ICA, EEG data were epoched from —750 to 1,500 ms relative to an event: the onset of EMS (StartEMS). This epoching window was chosen to minimize edge artifacts in the time-frequency analysis and epoch-containing artifacts (e.g., EEG amplitude exceeding 150 μV). Baseline correction was applied using the –500 to –100-ms interval to exclude potential motor command activity associated with movement imagery at EMS onset, given that the timing of the imagery-related command at approximately 0 ms was expected to vary across trials.

Subsequently, analyses were conducted to identify periods during which neural markers were modulated by the error condition and by the stimulus-driven attention condition, manipulated through EMS presentation rate and intensity. We tested whether these effects scaled across condition levels. The full set and its subsets were examined: (1) the All set, which included all combinations of EMS condition and error condition; (2) the Error-focused subset, in which EMS was fixed at low intensity and high presentation rate (LowI/HighP); and (3) the Attention-focused subset, where the EMS-induced movement matched the visual target (no-Error). For each set, ERP amplitudes were quantified, and time-frequency analyses were performed. Additionally, topographical maps were generated for selected time windows to confirm whether the observed neural activity originated from the representative channels defined in the subsequent statistical analysis section. The following representative channels were selected for analysis: FCz, Pz, C1, C3, and C5. FCz was included because early ERP (N180 and P300) components as well as theta, beta, and gamma activities are commonly investigated at this site (Iwane et al., 2023; Polich, 2007). Pz was analyzed since it reflects the late-positive component (Hajcak et al., 2010; Polich, 2007). In addition, contralateral motor areas (C1, C3, and C5) were examined because activity in the mu-band over these regions has been associated with motor execution and observation, particularly during wrist movements (Shibuya et al., 2018; Evans and Blanke, 2013).

For ERP amplitude quantification at FCz, the N180 component was defined as the first deflection within an 80-ms window (140–220 ms), and the P300 component as the second deflection within an 80-ms window (300–380 ms). These time windows were determined according to the data in previous studies (Iwane et al., 2023; Holeckova et al., 2006; Yasuhara et al., 2024). In an exploratory analysis, the late-positive component at Pz was strongly associated with error and consistent with context updating (Boldt and Yeung, 2015; Steinhauser and Yeung, 2010; Ridderinkhof et al., 2009; Overbeek et al., 2005; Nieuwenhuis et al., 2001; Li et Q. al., 2022; Gomez-Andres et al., 2024). Based on visual inspection, we defined a 500–900-ms window. We selected this broad range to accommodate potential latency variability across modalities [e.g., tactile studies report a late positive peak at approximately 550 ms (Zhang et al., 2016)].

To minimize bias from window selection, we averaged across cognitive conditions and focused on high-Error trials, since other error conditions in the All set could be influenced by EMS intensity. From the grand average, we identified the center latency for the late positivity (700 ms) and quantified the mean amplitude (not peak) within a 200-ms window centered on this latency (700 ± 100 ms; 600–800 ms). For the Error-focused subset, the same center latency and window derived from the All set were used. For the Attention-focused subset, the center latency obtained from the grand average (across cognitive and EMS conditions) was 608 ms; accordingly, mean amplitude was computed in a 200-ms window centered on this latency (608 ± 100 ms; 508–708 ms). Subsequently, grand averages were calculated for each condition (cognitive and error conditions), and individual ERPs were obtained from each participant. For the time-frequency analysis, data were epoched from –750 to 1,500 ms following ICA. Morlet wavelet time-frequency decomposition (Tallon-Baudry et al., 1997; Cohen, 2019) implemented in MNE-Python was applied.

Our analytical approach was similar to that used in a previous study investigating oscillatory signatures during a visually guided visuomotor rotation task with stepwise error conditions (Iwane et al., 2023). Following their method, event-related spectral perturbation analysis (Grandchamp and Delorme, 2011) was applied to each single-trial EEG epoch. Frequencies ranged from 3 to 100 Hz, and the number of cycles varied from 3 to 20 on a logarithmic scale. For each participant and condition, time–frequency power values were averaged across trials and then log-transformed (10-base logarithm). To avoid potential circularity arising from motor-imagery-related activity during the pre-stimulus period, no baseline correction was applied for this time-frequency analysis.

For StartEMS-locked analyses, time-frequency coefficients were averaged over time within the EMS period (0–1,000 ms). The frequency bands analyzed were selected based on previous studies linking them to cognitive and sensorimotor processes. We analyzed theta (4–8 Hz) (Iwane et al., 2023; Moreau et al., 2023; Cavanagh and Frank, 2014; Omedes et al., 2015), high-beta (18–30 Hz) (Jahani et al., 2020; Griffin et al., 2025), and high-gamma (60–90 Hz) (Iwane et al., 2023; Völker et al., 2018) at FCz, reflecting unpredictability and error-related processing. In addition, we examined mu (8–13 Hz) over contralateral sensorimotor sites (C1, C3, C5), related to congruence during motor observation (Shibuya et al., 2018; Evans and Blanke, 2013). The mu-power was denoted as the average spectral power across these electrodes.

For each frequency band, the average spectral power across time and frequency was used as a dependent variable. Two separate statistical analyses were conducted: within-subject factors included (i) error condition × cognitive condition and (ii) stimulus-driven attention condition × cognitive condition. Additionally, time-frequency-based statistical testing was performed to identify the time windows during which these factors significantly influenced the dependent measures.

### 3.5 Statistical analysis

We adopted the same sample size (*n* = 16) used in previous studies that examined error-related potentials and spectral power during error processing (Iwane et al., 2023). Statistical analyses were conducted using Python (v3.12.7), MNE-Python (v1.8.0), and statsmodels (v0.14.4). For behavioral data (questionnaire ratings), event-related potentials, and time-frequency analyses (average spectral power after event onset), normality was assessed using the Shapiro–Wilk test (*p*>0.05). Normality was confirmed for all behavioral ratings. Therefore, two-way repeated-measures analysis of variance (ANOVA) was applied to the behavioral data. Each neural response was analyzed at its corresponding representative channel.

The ERP component and time-frequency data violated normality. Therefore, a two-way repeated-measures ANOVA based on the aligned rank transform (ART) method was applied (Wobbrock et al., 2011). This nonparametric approach allowed detection of main effects and interactions without relying on distributional assumptions. When significant main effects or interactions were observed, *post-hoc* comparisons were performed using ART-C (Elkin et al., 2021).

For the All set analyses, we limited the hypothesis-driven tests to seven predefined EEG measures: theta (Iwane et al., 2023; Moreau et al., 2023; Cavanagh and Frank, 2014; Omedes et al., 2015), high-beta (Jahani et al., 2020; Griffin et al., 2025), and high-gamma (Iwane et al., 2023; Völker et al., 2018) at FCz; mu-band power at C1/C3/C5 (Shibuya et al., 2018; Evans and Blanke, 2013); and the N180 and P300 at FCz and the late positive ERP component at Pz (Iwane et al., 2023; Vocat et al., 2011; Boldt and Yeung, 2015; Steinhauser and Yeung, 2010; Ridderinkhof et al., 2009; Overbeek et al., 2005; Nieuwenhuis et al., 2001). Holm correction (Holm, 1979) was applied across these seven tests (*m* = 7). In the Error-focused and Attention-focused analyses, all exploratory outcomes across the two subsets were treated as a single family, and Holm correction was applied across this family (*m* = 14). For pairwise comparisons within the exploratory analyses, the Holm correction was applied across the total number of pairwise tests, with the number of comparisons defined as 14 × (number of condition pairs) (e.g., *m* = 14 × 3 = 42, or *m* = 14 × 1 = 14 when only one comparison was tested). Both uncorrected and Holm-corrected *p*-values are reported throughout.

To determine whether the trial exclusion criteria influenced the EEG results, we conducted additional sensitivity analyses using three predefined target-acquisition thresholds: (i) the standard threshold (±6°) used in the main analysis, (ii) stricter threshold (±5°), and (iii) more lenient threshold (±7°). We then repeated the primary EEG analysis—the two-way repeated-measures ANOVA—under each threshold to evaluate the robustness of the findings.

In the time–frequency analysis, spectral power was smoothed across time and frequency, requiring additional procedures to determine when condition differences emerged. For each frequency band and pairwise contrast, a cluster-based permutation test (3,000 iterations) was applied to within-subject difference maps using subject-wise sign-flip permutations to test whether the mean difference deviated from zero. This procedure inherently corrects for multiple comparisons across all time–frequency points within each contrast. However, this internal correction does not account for the number of contrasts. Therefore, in the All set analyses, which were hypothesis-driven, the Holm correction was applied across 16 contrasts (4 measures: theta, beta, gamma at FCz and mu at C1/C3/C5 × 4 condition contrasts: 3 Error contrasts + 1 Cognition contrast). In the Error- and Attention-focused subsets, which were exploratory, the Holm correction was applied across 32 contrasts (16 contrasts × 2 subsets), accounting for both the number of measures and the number of subsets.

## 4 Results

### 4.1 Behavioral data

In this study, we manipulated the magnitude of passive movements induced by EMS in response to a visually presented target. Three levels of EMS intensity were applied individually to each participant, modulating the degree of wrist flexion. As both the movement trajectories and questionnaire during the waiting session did not differ substantially from those observed in the imagery session, they are reported in Supplementary Figures S4, S5.

**Figure 3:**
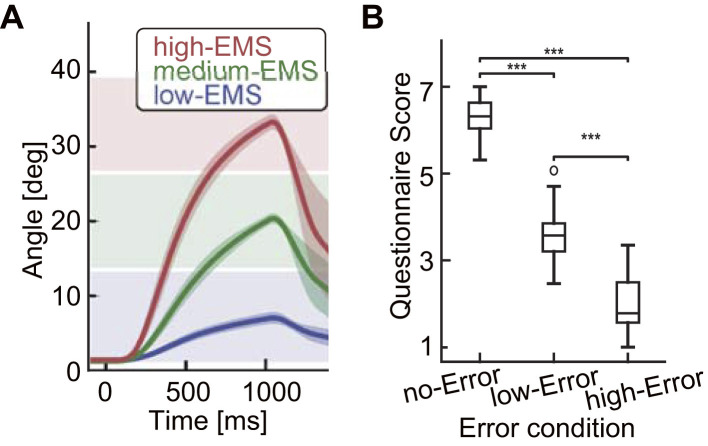
Trajectories of wrist angles across varying levels of EMS intensity. **(A)** Mean trajectories of wrist angles across varying levels of EMS intensity. Each line color represents a different intensity condition. The 95% confidence interval was estimated using the t-distribution based on the mean trajectory and standard error. Transparent colors of the three squares in the background indicate the range of the target for induced movement. **(B)** Boxplot of questionnaire responses ranging from 1 (not congruent at all) to 7 (fully congruent) for each error condition under imagery sessions. *** denotes a statistically significant difference with a *p*-value (*p* < 0.0001).

As shown in Figure 3A, during the imagery session, wrist movement began approximately 194 ms after EMS onset across all EMS intensity levels. At approximately 50 ms after the end of EMS (i.e., 1,050 ms), the wrist began returning to the neutral position. In the conditions of high EMS intensity, which induced movement toward the far-target position, the trajectory reached the medium-target zone at approximately 350 ms and the far-target zone between 600 and 700 ms. In the conditions of medium EMS intensity, the trajectory reached the medium-target at approximately 600 ms. In the condition of low EMS intensity, wrist movement remained within the near-target zone from the beginning. The participants rated the degree of congruence between intended and induced movements on a 7-point scale. The questionnaire was administered for each error condition (no-Error, low-Error, high-Error). A two-way repeated-measures ANOVA with factors of error condition and cognitive condition (imagery, waiting) revealed a significant main effect of error condition [*F*_(2, 30)_ = 249.67, *p* < 0.0001], indicating that higher error conditions were associated with lower subjective ratings of movement congruence (Figure 3B). No main effect of cognitive condition was observed. The detailed statistical results for the main effects of the error and cognitive conditions as well as their interactions are reported in Table 1. Pairwise comparisons confirmed significant differences between all error conditions (*p* < 0.001), with large effects of *r* = 0.957 (no- vs. low-Error), *r* = 0.978 (no- vs. high-Error), and *r* = 0.960 (low- vs. high-Error), suggesting that participants reliably distinguished the magnitude of errors between induced and ideal movements.

**Table 1:** Detailed results of the two-way repeated-measures analysis of variance examining the effects of error condition, cognitive condition, and interaction.

**Factor**	**Comparison**	***df*_1_**	***df*_2_**	***p*-value**	***p*-value (corrected)**	***F***	**Generalized η^2^**	***r* effect**	**ϵ**
Error	–	2	30	< 0.0001	< 0.0001	249.67	0.882	0.945	0.714
	No- < low-error	–	–	< 0.0001	< 0.0001	–	–	0.957	–
	No- < high-error	–	–	< 0.0001	< 0.0001	–	–	0.978	–
	Low- < high-error	–	–	< 0.0001	< 0.0001	–	–	0.960	–
Cognition	–	1	15	0.2162	0.2162	1.67	0.007	0.317	1.000
Error × cognition	–	2	30	0.1332	0.1512	2.16	0.012	0.259	0.703

Normality was verified using the Shapiro–Wilk test (*p*>0.05). Greenhouse–Geisser correction was applied when necessary, based on ϵ values.

### 4.2 EEG data

#### 4.2.1 ERPs

**Figure 4:**
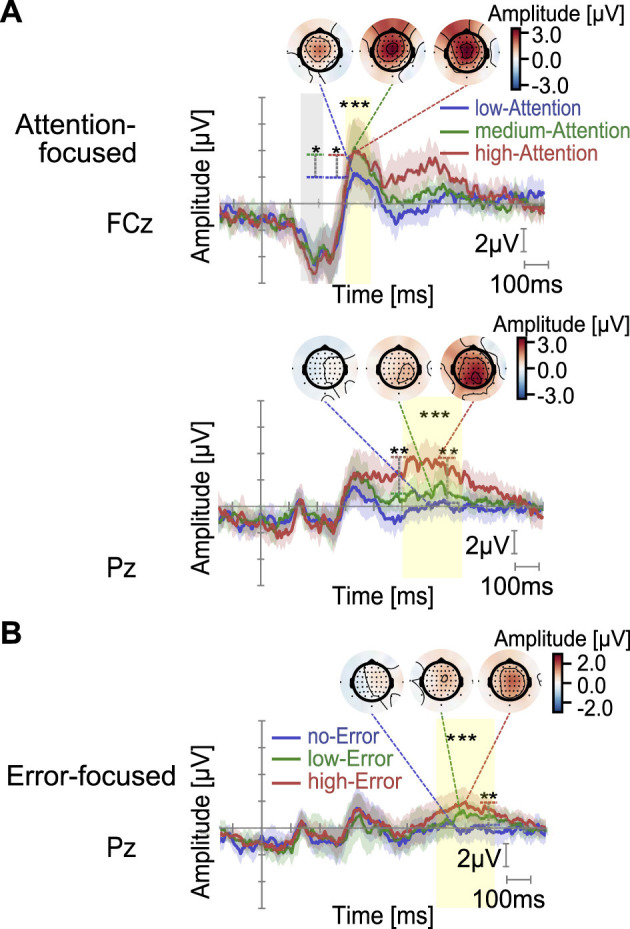
ERP waveforms, topographical maps, and statistical results are shown for the error and stimulus-driven attention conditions (* : *p* < 0.05, ** : *p* < 0.01, and *** : *p* < 0.001). **(A, B)** ERP waveforms and topographies are presented for each condition: **(A)** Attention-focused subset and **(B)** Error-focused subset. EEG amplitude (2 μV/div) and time (100 ms/div) are shown on the vertical and horizontal axes, respectively. Shaded areas represent 95% confidence intervals based on the t-distribution. As imagery and waiting conditions showed no significant main effect on ERP peaks or means, the plots present averages across both conditions. Colored rectangles denote the two-way repeated-measures ANOVA time windows: gray for non-significant and yellow for main effects of stimulus-driven attention **(A)** or error level **(B)**. In both Attention-focused and Error-focused subsets, ERP waveforms and scalp topographies are shown only for components that reached statistical significance in the respective analyses.

Our objective was to investigate whether ERP components at StartEMS were modulated by the error condition, cognitive condition, and stimulus-driven attention. We analyzed two representative channels: FCz and Pz, where error-related potentials are typically observed. ERP components were identified (see Figure 4), including a negative component at approximately 180 ms and positive components at approximately 300 ms and after 450 ms in the EEG time series.

Furthermore, the topography shown in Figures 4A, B (corresponding to the Attention-focused subset and Error-focused subset, respectively) revealed spatially consistent patterns for the negative ERP component (at approximately 180 ms), the 300 ms component, and the later component (after 450 ms), both across the three overall conditions and across error or EMS intensity conditions within each subset. The negative ERP appeared to originate from the left prefrontal area, the 300-ms component, from the sensorimotor region along the midline, and the late component, from both the sensorimotor region and parietal regions. In the All set, however, no significant effects of error or cognitive condition were observed at FCz (N180, P300) or at Pz (late positive component). The ERP waveforms for each cognitive condition and the corresponding topographies at the investigated time windows are reported in Supplementary Figures S6, S7. In the Attention-focused subset (Figure 4A), where no error was present, we investigated whether ERP amplitudes reflected the effects of stimulus-driven attention via the intensity and frequency of EMS. As shown in the topographies of Figure 4A, components at approximately 300 ms and 600 ms exhibited activity over the midline central regions (FCz and Pz, respectively). A two-way repeated measures ANOVA using ART revealed significant main effects of stimulus-driven attention condition for the 300-ms component at FCz [*F*_(2, 75)_ = 8.73, *p* = 0.0004] and for the 600-ms component at Pz [*F*_(2, 75)_ = 23.89, *p* < 0.0001]. *Post-hoc* pairwise comparisons showed that at FCz (300 ms), ERP amplitudes were larger in the high-Attention and medium-Attention condition than in the low-Attention condition (*p* = 0.0011, *r* = 0.366 and *p* = 0.0003, *r* = 0.402, respectively), while at Pz (600 ms), the ERP amplitude at high-Attention exceeded the amplitudes at both medium-Attention (*p* = 0.0001, *r* = 0.435) and low-Attention conditions (*p* < 0.0001, *r* = 0.621). In the Error-focused (LowI/HighP) subset (Figure 4B), where only low-EMS intensity was used, the influence of EMS intensity was minimized, allowing for a clearer assessment of error condition effects on ERP amplitudes. As shown in Figure 4B, the analyses focused on the late component at Pz (700 ms) showed a significant effect in the All set. Two-way repeated measures ANOVA using ART revealed significant main effects of the error condition on this component [*F*_(2, 75)_ = 11.81, *p* < 0.0001]. *Post-hoc* pairwise comparisons further confirmed that this component was significantly greater in the high-Error condition than in the no-Error condition (*p* < 0.0001, *r* = 0.489). The detailed statistical results for ERP and time–frequency measures are summarized in Table 2.

**Table 2:** Effects of error and cognitive condition on target frequencies at representative channels.

**Set**	**Ch**	**Measure**	**Factor**	**Comparison**	***p*-value**	***p*-value**	***F*(*df*_1_, *df*_2_) (corrected)**	**Partial η^2^**	***r* effect**
All set	C135	Mu	Error	–	0.0001	0.0007	10.42 (2, 75)	0.217	0.466
				No- < high-factor	0.0001	0.0021	–	–	0.466
	FCz	Beta	Cognition	–	< 0.0001	< 0.0001	27.07 (1, 75)	0.265	0.515
				Imagery < waiting	< 0.0001	< 0.0007	–	–	0.515
	FCz	Theta	Cognition	–	0.0015	0.0087	10.92 (1, 75)	0.127	0.357
				Imagery < waiting	0.0015	0.0105	–	–	0.357
Error-focused subset	Pz	Late Pe	error	–	< 0.0001	0.0004	11.81 (2, 75)	0.240	0.489
				No- < high-factor	< 0.0001	0.0042	–	–	0.489
	C135	Mu	Error	–	< 0.0001	< 0.0001	17.50 (2, 75)	0.318	0.564
				no- < high-factor	< 0.0001	< 0.0042	–	–	0.564
	FCz	Theta	Error	–	0.0032	0.0320	6.21 (2, 75)	0.142	0.377
	FCz	Beta	Cognition	–	0.0002	0.0032	15.02 (1, 75)	0.167	0.408
				Imagery < waiting	0.0002	0.0028	–	–	0.408
Attention-focused subset	Pz	late Pe	Attention	–	< 0.0001	< 0.0001	23.89 (2, 75)	0.389	0.624
				Low- < high-factor	< 0.0001	0.0042	–	–	0.621
				Med- < high-factor	0.0001	0.0042	–	–	0.435
	FCz	P300	Attention	–	0.0004	0.0043	8.73 (2, 75)	0.189	0.434
				Low- < med-factor	0.0011	0.0407	–	–	0.366
				Low- < high-factor	0.0003	0.0114	–	–	0.402
	FCz	Beta	Cognition	–	0.0016	0.0225	10.72 (1, 75)	0.125	0.354
				Imagery < waiting	0.0016	0.0224	–	–	0.354

A two-way repeated-measures ANOVA was conducted using the Error-focused subset, and only significant results. C135 denotes the average value across channels C1, C3, and C5. In the “Comparison” column, “factor” automatically indicates the corresponding factor in the “Factor” column (e.g., “no- < high-factor” under error means “no- < high-Error”).

#### 4.2.2 Time-frequency analysis

As in the ERP analysis, we investigated whether band power was modulated by the error condition and cognitive condition (Table 2). Specifically, we examined mu-power at the contralateral sensorimotor electrodes (C1, C3, C5) as an index of prediction-related processes, whereas theta-, beta-, and gamma-power at FCz were analyzed as neural markers of error-related processing.

Beta-, mu-, and theta-band activity across the All set and the three subsets was subjected to time-frequency analysis. Significant effects were observed in all frequency bands, and the detailed statistical values for each dataset are summarized in Table 2.

For beta-power, all datasets—including the All set, the Error-focused, and Attention-focused subset—consistently showed a significant main effect of the cognitive condition [*F*_(1, 75)_ = 27.07, *p* < 0.0001; *F*_(1, 75)_ = 15.02, *p* = 0.0002; *F*_(1, 75)_ = 10.72, *p* = 0.0016, respectively], with higher beta-power in the waiting condition than in the imagery condition.

Across both the All set and Error-focused subsets, mu-power at the contralateral sensorimotor electrodes (C1, C3, C5) showed a significant main effect of error condition [*F*_(2, 75)_ = 10.42, *p* = 0.0001; *F*_(2, 75)_ = 17.50, *p* < 0.0001, respectively]. Because these effects were replicated in both sets, they likely reflect differences in error condition rather than stimulus-driven attention. In addition, pairwise comparisons consistently revealed that mu-power was significantly lower in the no-Error condition than in the high-Error condition (*p* = 0.0001, *r* = 0.466, and *p* < 0.0001, *r* = 0.564, respectively). Theta-power showed a significant main effect of cognitive condition in both sets, but with different patterns. In the All set, a significant main effect of cognitive condition was found at FCz [*F*_(1, 75)_ = 10.92, *p* = 0.0015], and pairwise comparisons revealed that theta-power was significantly larger in the waiting condition than in the imagery condition (*p* = 0.0015, *r* = 0.357). By contrast, in the Error-focused subset, theta-power showed a significant main effect of the error condition [*F*_(2, 75)_ = 6.21, *p* = 0.0032); however, none of the pairwise comparisons reached significance. In addition, we evaluated whether trial exclusion criteria affected the EEG results by performing sensitivity analyses using stricter (±5°) and more lenient thresholds (±7°). These analyses yielded largely consistent results with the standard threshold (±6°) results.

While the main findings remained unchanged, however, ERN and FCz beta activity showed threshold-dependent variability. For ERN, the standard threshold (±6°) was non-significant after correction [*p* = 0.0425, *F*_(2, 75)_ = 3.29], whereas both the stricter (±5°) and more lenient thresholds (±7°) yielded significant effects [stricter: *p* = 0.0013, *F*_(2, 75)_ = 7.31; more lenient: *p* = 0.0014, *F*_(2, 75)_ = 7.15]. For beta activity, significant effects were observed at standard and more lenient thresholds [standard: *p* = 0.0016, *F*_(1, 75)_ = 10.72; more lenient: *p* = 0.0018, *F*_(1, 75)_ = 10.46], but the stricter threshold did not survive correction [*p* = 0.0055, *F*_(1, 75)_ = 8.18]. Two-way repeated measures ANOVA results and trial retention rates are reported in Supplementary Tables S3–S5.

**Figure 5:**
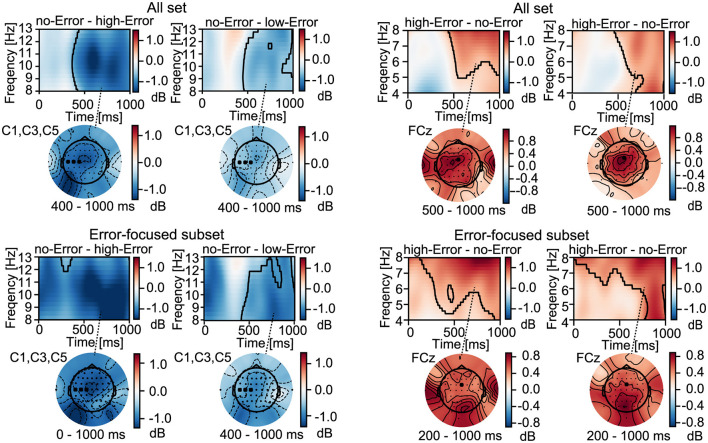
Time-frequency maps and topographies. Time-frequency maps and topographies illustrate significant differences in the Error-focused (LowI/HighP) subset, as identified by both pairwise comparisons and cluster-based permutation tests. The time-frequency map was averaged across conditions with no main effects. Significant clusters are outlined in black.

Next, we conducted cluster-based permutation tests on the time–frequency maps to identify temporal clusters showing significant differences between the conditions. These tests were corrected across bands, datasets, and condition-comparison patterns. The uncorrected and corrected *p*-values for all significant clusters are provided in Supplementary Table S1. Significant clusters were found in both the All set and the Error-focused subset, as shown in Figure 5.

For the mu-band, significant clusters were identified at the contralateral sensorimotor electrodes (C1, C3, C5) in both datasets, where mu-power in the no-Error condition was significantly more suppressed than that in the error conditions at 400–1,000 ms. Inspection of the topographical maps for this time window revealed that the maximal difference between the no-Error and error conditions was centered near Cz along the midline in both datasets. In the Error-focused subset, the contrast between the no-Error and high-Error conditions exhibited a broader temporal extent, with differences present throughout the entire analysis window. An electrode-wise inspection showed a Cz-centered peak in mu activity, while the magnitude of mu suppression appeared broadly similar across sensorimotor electrodes, suggesting that using C1, C3, and C5 as representative sites provides an appropriate summary of mu suppression (Supplementary Figure S8).

For the theta-band, significant clusters were identified in both the All set and the Error-focused subset in the comparisons between the no-Error and high-Error conditions as well as between the no-Error and low-Error conditions. In the All set, a significant cluster emerged at 500–1,000 ms, with the largest differences appearing near FCz. Such error-related increases in theta activity have commonly been reported at FCz (Iwane et al., 2023; Moreau et al., 2023; Cavanagh and Frank, 2014; Omedes et al., 2015). In the Error-focused subset, significant clusters appeared earlier and extended across a broader temporal range, spanning approximately 200–1,000 ms. Inspection of the topographical maps for this time window showed that the maximal differences between the no-Error and error conditions were located not near FCz but rather over parietal to occipital regions, with a similar spatial pattern observed for both error contrasts.

## 5 Discussion

This study examined how EEG neural markers during passive movements induced by EMS were modulated by three factors: (1) motor error (no-, low-, high-Error), (2) stimulus-driven attention (manipulated by EMS intensity and presentation rate), and (3) motor imagery (imagery vs. waiting).

### 5.1 ERPs associated with motor error and stimulus processing

On comparing ERP peak or mean values, we found that the Attention-focused (no-Error) subset showed a main effect of stimulus-driven attention at FCz for the P300 and at Pz for the late-positive component (508–708 ms). In contrast, the Error-focused (LowI/HighP) subset showed a main effect of error condition at Pz for the late-positive component (600–800 ms).

The P300 typically peaks in the fronto-central region (FCz) and reflects detection of error (Iwane et al., 2023; Vocat et al., 2011) or low-presentation rate stimuli in oddball tasks (Polich, 2007; Wessel et al., 2012). The P300 at FCz in this study was not significantly modulated by error condition; instead, it showed a main effect of stimulus-driven attention (controlled EMS intensity and presentation rate) in the Attention-focused subset. As indicated by the FCz/Cz peak in Figure 4, this positivity is most consistent with a P3a reflecting orienting to unpredicted stimuli or strong stimulus intensity (Polich, 2007). However, because its scalp distribution partially overlaps with the subsequent error-related positivity in the Error-focused subset, the possibility that it forms part of an error-processing cascade cannot be entirely excluded, and we, therefore, refrain from making a definitive claim that it corresponds to a P3a response. Clarifying whether these components arise from shared or distinct neural mechanisms will require source estimation based on artifact-reduced data, such as those obtained from mechanically guided passive movements or paradigms with minimal movement variability.

In the Attention-focused subset, a parietal positivity centered at 608 ms was maximal at Pz and larger under high-Attention than under low- and medium-Attention conditions (Figure 4). Accounting for the 194-ms delay from EMS to induced-movement onset, with the peak observed at approximately 420 ms post-movement. Given its phasic profile and parietal maximum, this late positivity is consistent with a P3b (Polich, 2007), likely reflecting context updating or controlled attention to rare, task-relevant induced-movement events. Although the 194-ms EMS-movement delay was relatively consistent across stimulation and cognitive conditions and is therefore unlikely to distort the relative differences observed between conditions, this fixed temporal offset nonetheless imposes a limitation on the temporal interpretation of the ERP components. In particular, ERP components after EMS onset may partially reflect processes time-locked to the onset of the induced movement rather than strictly stimulus-evoked responses, making it difficult to fully dissociate these contributions. Future work should use paradigms that minimize the stimulation-movement onset delay—such as mechanically guided movements or short-duration button-press tasks—to determine whether comparable ERPs arise when this temporal offset is removed.

In the Error-focused subset, the scalp map shows a broad centro-parietal positivity with a Cz maximum. With a stimulus-locked peak at 700 ms (500-ms post-movement after the 194 ms delay), the pattern is more consistent with an early- or late-Pe account (detection or evaluation) (Boldt and Yeung, 2015; Steinhauser and Yeung, 2010; Ridderinkhof et al., 2009; Overbeek et al., 2005; Nieuwenhuis et al., 2001). Although a P3b-like context-updating account cannot be excluded—far-target trials received three EMS intensities, near-target trials received only one—in the Attention-focused (no-Error) subset, the P3b was Pz-maximal, whereas the present effect was Cz-centered. We, therefore, interpret it primarily as a motor-error-related component. Future work should test whether this late-positive component predicts subsequent post-error voluntary motor performance. Furthermore, our results suggest that error-related information may emerge at approximately 700 ms after the Pe, but whether participants consciously recognized the error at that time is unclear. Inter-participant variability in error awareness is also possible, and additional behavioral or subjective measures are needed to quantify this perceptual delay. Finally, the unequal number of trials across subsets may have affected the stability of the averaged data, underscoring the need to balance trial numbers in future studies.

In summary, during the passive motor error presentation task, we suppose that an early stimulus-driven attentional response resembling a P3a component occurs, followed by either reallocation of attention and context updating (P3b) or conscious error recognition and evaluation (late Pe), depending on the stimulus/error condition. Whether these two processes operate independently or stem from a common mechanism remains unclear, and future experiments should be designed to clarify this point.

### 5.2 Theta, mu, and beta band activity across cognitive and error conditions

To complement the ERP findings, we analyzed theta- and mu-band activity across cognitive and error conditions. As demonstrated in prior studies (Iwane et al., 2023; Moreau et al., 2023; Cavanagh and Frank, 2014; Omedes et al., 2015), theta-power increases in response to unpredictable contexts. Our results showed that FCz theta increased whenever internal predictions were absent or violated, suggesting a common mechanism whereby theta activity tracks outcomes that are incongruent with predictions. In the All set, theta activity was larger during the waiting condition than during the imagery condition, reflecting greater unpredictability without internal predictions. In the Error-focused subset, theta scaled with error level, indicating sensitivity to externally induced Incongruence. Although ANOVA and permutation tests emphasized different factors, both showed that FCz theta tracked incongruence with predicted states, and a true Error × Cognition interaction may have gone undetected due to limited power.

A posterior–parietal theta peak also appeared in the Error-focused subset. Posterior theta activity can reflect sensory conflict or incongruence with predicted visual input in other paradigms (Haciahmet et al., 2023), although these arise in different contexts. Thus, while a posterior contribution to incongruent processing is possible, the present effect remains uncertain and may instead reflect task-specific visual factors. The mu-band power suppression in the motor cortex may primarily reflect the match between predicted and actual sensory inputs during motor observation. Although this task involves passive movement, the EMS provides motor-related sensory feedback during imagery, suggesting that it has characteristics of both motor execution and motor observation. Consistent with findings from motor execution and motor observation studies (Shibuya et al., 2018; Evans and Blanke, 2013), significant mu-band suppression in the contralateral sensorimotor cortex (C1, C3, and C5) was observed when predicted and actual sensory input were matched. Furthermore, the topographic map between 400 and 1,000 ms, which was significant in the cluster-based permutation test, revealed a clear difference between the high-Error and no-Error conditions in the motor cortex.

Beta-band activity has been proposed to decrease in situations where the stability of the current sensorimotor or cognitive state is not expected to be maintained (Engel and Fries, 2010). During motor imagery, participants generate an internal forward prediction of the upcoming internally sensory state. Because EMS provides sensory input that does not match this internally sensory state, the imagery condition likely places the system in a mode where a deviation from internal state is anticipated once stimulation occurs. Such anticipation that the incoming sensory input may diverge from the internally predicted state provides a parsimonious explanation for the reliable beta suppression observed in the imagery condition, whereas the absence of any internally predicted sensory state in the waiting condition is consistent with a relatively higher beta power.

### 5.3 Error processing during motor imagery

Initially, we hypothesized that the cognitive condition would interact with the error condition to modulate error-related neural activity. However, error-related neural activity was not significantly influenced by the cognitive condition. One possible reason is that repeated EMS may disrupt motor imagery, reducing its consistency or realism over time. Therefore, experimental designs that better align imagery with realistic motor execution are needed. However, this explanation remains speculative, as the present dataset lacks direct measures. Including trial-by-trial imagery vividness in future studies would allow this possibility to be tested.

The next question is where differences between cognitive conditions might emerge. Previous studies have shown that individuals with motor experience exhibit lower and more stable mu-band power during motor planning and imagery (Wolf et al., 2014; Zabielska-Mendyk et al., 2018; Percio et al., 2010). In addition, during motor imagery, forward predictions of sensory inputs are internally generated (Kilteni et al., 2018) and compared with actual outcomes, elicited by EMS-induced sensations. Based on these data, we hypothesized that motor imagery enhances the stability of error-related neural markers, and this stabilization is modulated by individual differences in sensorimotor representations reflected in mu-band oscillations.

**Figure 6:**
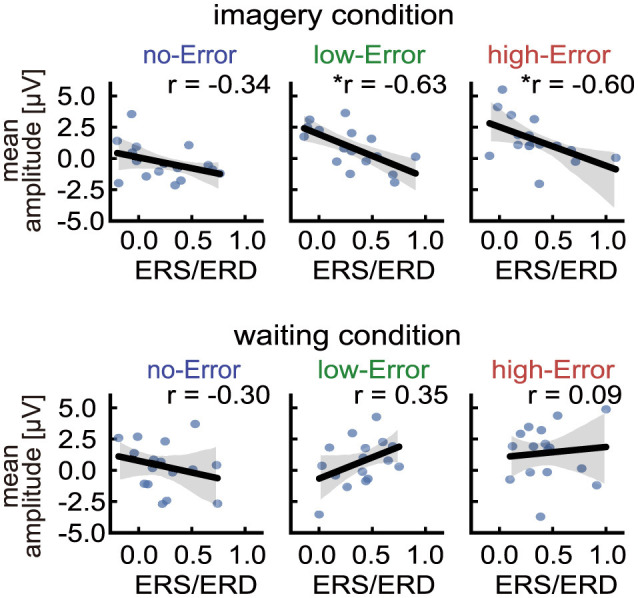
Between-participant correlations between late-positive component mean amplitude (600–800 ms) and mu-band ERD/ERS (Cz, C1, and C3). Between-participant correlations were examined across participants in the Error-focused subset using Spearman's correlation. Scatter plots are shown separately for no-, low-, and high-Error trials under imagery and waiting conditions. Significant negative correlations were found in the imagery condition for low-Error (*p* = 0.0094) and high-Error (*p* = 0.0140). The Benjamini–Hochberg correction was applied across six comparisons (2 conditions × 3 Error levels), and both correlations remained significant after correction (BH-adjusted *p* = 0.042 for each).

Importantly, significant negative correlations between mu-band power in the contralateral motor cortex (Cz, C1, and C3) and the late-positive component were observed only in the imagery condition for low- and high-error trials (Figure 6), whereas no such relationship was found in the waiting condition. This pattern suggests that the influence of motor imagery ability on error processing emerges when internally generated motor predictions are actively compared with external sensory feedback, consistent with forward-model accounts of predictive control (Kilteni et al., 2018; Friston, 2010). Although this finding aligns with the forward-model interpretation, alternative explanations cannot be excluded. Individual differences in imagery ability may also reflect variability in predictive sensory gating or efficient reallocation of attentional resources between internal simulation and external feedback (Blakemore et al., 2000; Wessel, 2018; Polich, 2007).

Overall, these results indicate the possibility that error processing during motor imagery may be influenced by both the precision of internal sensory predictions and attentional modulation, with individual differences potentially arising from how precisely and flexibly these processes are engaged. To directly examine these possibilities, future studies should include trial-by-trial measures of imagery vividness, enabling a more precise evaluation of how imagery quality shapes error-related neural responses. Such measures would also allow single-trial modeling approaches to clarify how imagery proficiency and prediction accuracy contribute to individual variability in error sensitivity.

### 5.4 Methodological limitations

One of the methodological limitations concerns the reproducibility of the stimulation protocol. In this study, EMS intensity and the resulting movement amplitude were manually adjusted for each participant and session to compensate for individual differences in muscle responsiveness and fatigue. This approach ensured consistent movements among the participants but inherently limits the standardization of stimulation parameters across studies. In future iterations of this paradigm, we aim to incorporate automated or semi-automated calibration procedures and to align our documentation with emerging community frameworks such as the BEST toolbox (Hassan et al., 2022) to facilitate standardized stimulation reporting and the COBIDAS guidelines (Nichols et al., 2017) to facilitate transparent neurophysiological data documentation—as these developments are expected to improve methodological transparency and facilitate cross-study comparability. A further limitation concerns the absence of an explicit measure of sense of agency, such as intentional binding. Although the imagery instructions were designed to engage internal forward predictions, incorporating agency-sensitive measures in future work would allow stronger inferences about prediction-based processing during EMS-induced movements.

In addition to these procedural considerations, we determined whether the current sample size provided sufficient statistical sensitivity for the main effects of interest. Because our EEG analyses relied on the non-parametric ART framework, conventional power analyses were not applicable. We, therefore, conducted *post-hoc* power analyses in G*Power (Faul et al., 2007, 2009) using an approximated two-way repeated-measures ANOVA design that reflected the structure of the Error-focused subset. Although the three key measures (late Pe, theta, and mu) showed robust main effects of error magnitude and/or cognitive condition, theoretical accounts suggest that these processes may also interact. Because the expected two-way interactions were not significant, we specifically evaluated the power to detect these interaction terms. In the Error-focused subset, the estimated power was low (late Pe: 1−β = 0.28; theta: 0.17; mu: 0.08), suggesting that the absence of interaction effects may reflect limited sensitivity rather than a true lack of interaction. These null findings should therefore be interpreted cautiously and viewed as preliminary data. Importantly, these power limitations do not compromise the main conclusions of the study, which are based on robust and statistically reliable main effects. Taken together, our results support conclusions about the main effects, whereas inferences regarding the absence of interactions should be considered tentative. Further details of the power analysis procedures are provided in the Supplementary material.

To determine whether our preprocessing criteria and the uneven trial structure in the Error-focused subset affected the EEG results, we conducted two sensitivity analyses. First, we repeated all analyses using stricter (5°) and more lenient (7°) exclusion thresholds. Most effects were preserved, but ERN amplitude and beta-band activity at FCz varied across thresholds. Second, to correct trial-count imbalances in the Error-focused subset, we equated trial numbers within participants and performed 20 subsampling iterations, applying the two-way repeated measures ANOVA and same multiple comparison corrections as in the main analysis. Late Pe amplitude was reproduced in 19/20 iterations (95%), and mu-band suppression in 100%, whereas beta-, theta-, and ERN effects reappeared in 50%, 5%, and 5% of iterations, respectively. Detailed statistics for each iteration are provided in Supplementary material. Together, these results indicate that late Pe and mu-band oscillations in the Error-focused subset were the most robust neural response, consistently replicated across thresholds and subsampling, whereas theta-, beta-band, and ERN effects are less stable, likely due to lower signal-to-noise ratios and sensitivity to trial-count variability. These findings underscore the need for increased trial numbers, more uniform sampling of error levels, and the use of mechanically controlled passive-movement systems to reduce stimulation-related variability. Such refinements will support clearer dissociation of attention, agency, and internal prediction and improve the interpretability of oscillatory responses with weak effect stability.

## 6 Conclusion

This study investigated how neural responses are shaped by motor error, stimulus-driven attention, and cognitive condition during EMS-induced passive movements combined with motor imagery. By manipulating EMS intensity and presentation rate, we controlled both stepwise motor error magnitude and stimulus-driven attention. The results showed that mismatches between predicted and actual sensory inputs—despite the absence of voluntary movement—evoked distinct neural responses. Behaviorally, participants evaluated error levels in a stepwise manner, and this sensitivity corresponded to graded neural responses. A late positivity at 700 ms at Pz and mu-band suppression over the motor cortex reflected the stepwise levels of match between predicted and actual sensory inputs. In contrast, the P300 amplitude at FCz was driven by EMS intensity and presentation rate, consistent with attention to unpredicted sensory input. Theta-band enhancement without motor imagery suggested greater sensory unpredictability due to the absence of internal predictions, whereas beta-band activity may reflect fluctuations in state prediction. Additional analyses showed that motor imagery may reduce variability in error-related neural response, with this stabilization linked to individual differences in sensorimotor representations. Overall, the findings indicate that EEG signatures such as late positive ERP and mu activity are shaped not only by error and attention but also by internal predictive states. Future research should clarify how attention, error, and internal prediction contribute to sensorimotor incongruence, while addressing limitations such as low interaction power and uneven trial counts. Incorporating mechanically controlled passive movements may further separate sensory prediction from stimulation-specific variability and provide a more stable and controllable platform. These improvements will deepen our understanding of error monitoring and support applications in motor learning and rehabilitation.

## Data Availability

The raw data supporting the conclusions of this article will be made available by the authors, without undue reservation.
