# Supplementary material for: Electroencephalography signatures of motor error and stimulus-driven attention in electrical muscle stimulation-induced wrist movements under motor imagery
Source: Front Hum Neurosci. 2026 Jan 27;19:1713908. doi: 10.3389/fnhum.2025.1713908 (PMC12887888; doi:10.3389/fnhum.2025.1713908)
Supplement: Supplementary file 1 [file Data_Sheet_1.pdf]

Supplementary Material

1 SCREENING QUESTIONNAIRE

In this study, screening items were developed based on a Japanese textbook by Koji Shomoto (2017), Evidence-Based Physical Therapy, 1st Edition (YODOSHA CO., LTD.), to confirm the absence of skin abnormalities prior to the application of electrical stimulation. The screening form is presented below.

Q & A ____

If you cannot answer with 'No' or 'Yes' please provide additional details.

1. Q: Do you have any metal or electronic devices in your body? (e.g., cardiac pacemaker, implanted pumps, metal residues)
   A: ☐ No ☐ Yes
2. Q:Have you ever been diagnosed with any of the following conditions: deep vein thrombosis (DVT), infection, osteomyelitis, or tuberculosis?
   A: ☐ No ☐ Yes
3. Q: Have you experienced any skin problems (e.g., dermatitis, eczema)? If yes, please describe the affected areas.
   A: ☐ No ☐ Yes
4. Q: Is your skin sensitive to stimulation (including allergic reactions)?
   A: ☐ No ☐ Yes
5. Q: Do you currently have any bleeding areas on your upper limbs? If yes, please specify the location(s).
   A: ☐ No ☐ Yes

2 DESIGN OF EXPERIMENT AND STEPWISE PARAMETERS

This section describes the design of the experiment and the stepwise parameters used to separate the effects of motor error and stimulus-driven attention. Motor error was manipulated independently, whereas stimulus-driven attention was considered to be influenced by two factors, EMS intensity and stimulus presentation rate. The present analysis was not intended to determine which factor exerted a greater effect, but rather to parameterize both in a controlled, stepwise manner. All parameters were designed to vary stepwise in approximately linear increments, and values were normalized to a 0–1 scale for visualization. In addition, normalized values of motor error, EMS intensity, and presentation rate (Shannon entropy) were plotted for the All set, the Error-focused subset, and the Attention-focused subset (Figure S1), demonstrating that these indices exhibited approximately linear stepwise changes across All set and two subsets.

2.1 Motor Error

Motor error was manipulated by adjusting EMS intensity so that the induced wrist dorsiflexion deviated from the cued target within predefined angular ranges. Three targets were defined: near (7±6°), middle (20±6°), and far (33±6°) (Figure 2B). Error levels (no-, low-, high-Error) were assigned according to whether the induced movement fell into the cued or a non-cued target range. For quantification, motor error was defined by the central angles of the three ranges (7°,20°, and 33°) rather than trial-by-trial deviations. These values were normalized by the maximum (33°), yielding stepwise values within a 0–1 range, consistent across subjects.

2.2 EMS Intensity

EMS intensity was varied at three levels (low-, medium-, high-EMS). The applied current was first computed, then averaged within each subject across the three intensity levels, and subsequently normalized by the maximum value. These normalized values were then averaged across subjects to provide a between-subject measure of relative stimulus strength.

2.3 Stimulus presentation rate

Stimulus frequency was parameterized using Shannon information, which indicates that events with lower probability convey greater information. The Shannon information I for an event with probability p was defined as:

$$I = -\log_2 p$$

In the present experiment, the probability of EMS stimulation was set to three levels (high, medium, and low). Assuming a total number of trials

$$p_i = \frac{n_i}{N}$$

and the corresponding information content by is given by

$$I_i = -\log_2 \left(\frac{n_i}{N} \right)$$

As expected, lower-frequency conditions yielded larger information content. Information content was normalized by the maximum across the three conditions, yielding a range of 0–1 (Figure S1).

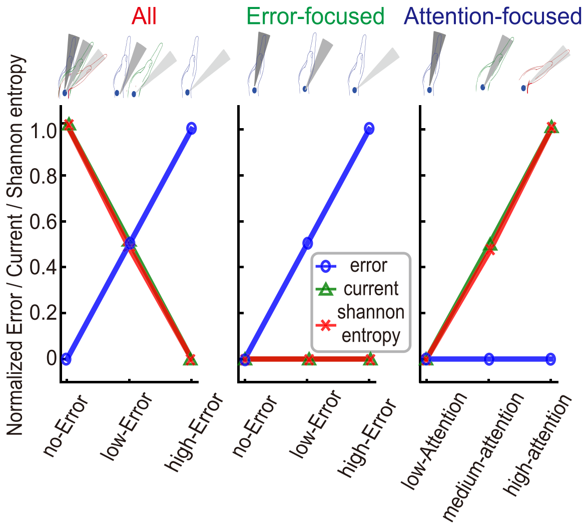

Figure S1: Normalized motor error, EMS intensity, and stimulus presentation rate for the All set, Error-focused subset, and Attention-focused subset. Normalized values of motor error, EMS intensity, and Shannon entropy (stimulus presentation rate) are shown for the All set, Error-focused subset, and Attention-focused subset. Each index was normalized to a 0–1 range and exhibited approximately linear stepwise changes.

3 VERIFICATION OF SESSION-TO-SESSION STABILITY OF EMS STIMULATION

In this study, passive movements induced by electrical muscle stimulation (EMS) were delivered across two sessions (imagery and waiting). To verify that the stimulation strength remained stable across these sessions, we conducted an additional analysis. Because EMS intensity directly affects the magnitude of the evoked muscle contraction and the associated somatosensory input, substantial variation in stimulation strength across sessions could alter the amplitude or direction of the induced movement and, consequently, introduce systematic differences in EEG responses. Therefore, confirming that stimulation parameters remained consistent across sessions is essential for ensuring the validity of the neural comparisons. To compare session-to-session variability independently of individual differences in absolute current amplitude, we applied min–max normalization to the stimulation current within each participant. The normalized value was computed as:

$$I_{\text{norm}} = \frac{I - I_{\text{min}}}{I_{\text{max}} - I_{\text{min}}},$$

where I_{min} and I_{max} denote the minimum and maximum stimulation current for that participant across all conditions (all levels × sessions × days). For each Subject × Day × Level, the session difference was computed as:

$$\Delta_{\text{session}} = I_{\text{norm}}^{(\text{Second})} - I_{\text{norm}}^{(\text{First})}.$$

These values were computed for all levels, averaged within each Subject × Days × Levels. Next, to assess how these normalized differences translate into the scale of evoked movement angles, we mapped the normalized current differences onto the range of EMS-induced movement magnitudes observed in the experiment. The induced movement angles ranged approximately from 1° to 39°,

providing an effective usable range of about 38°. Thus, a normalized session difference corresponds to the following angular change:

$$\Delta_\theta = \Delta_{\text{session}} \times 38^\circ$$

The task included three target locations (7°,20°, and 33°) , spaced by approximately 13° between target centers. This 13° interval defines the boundary for transitioning across movement categories (near-, medium-, and far-target). The min–max–normalized session differences were:

$$\Delta_{\text{session}} = -0.045 \pm 0.126$$

When converted to the behavioral scale, this corresponds to:

$$\Delta_\theta \approx -1.71^\circ, \quad SD \approx 4.79^\circ$$

Relative to the 13° spacing between the target centers, the magnitude of session-to-session variability was small. The mean change of approximately 1.71° corresponded to only about 13% of the target separation, and even the standard deviation of 4.79° amounted to roughly 37% of that spacing. Importantly, the maximal fluctuations observed in the data did not approach the 13° boundary required to shift an evoked movement from one target category to another. Thus, the observed variability in EMS stimulation strength was small relative to the induced movement range and insufficient to alter target-category assignments. Accordingly, it is unlikely that the session-wise differences in stimulation strength produced changes large enough to meaningfully affect the evoked movement or somatosensory input.

4 ICA-BASED ARTIFACT REMOVAL

ICA was performed for each participant by concatenating two consecutive blocks within the same session, rather than combining all blocks. This approach was chosen to avoid introducing excessive nonstationary activity while maintaining efficient manual inspection of ICA components. In addition to ocular and muscle artifacts, EMS-induced frequency-specific components were consistently identified and removed. Figure S2 shows the standard component properties generated by $mne.ica.plot_properties$, including the component topography, the epochs image with the corresponding event-related potential (ERP), and the component activation time course, the power spectrum, and the epoch variance. To illustrate that EMS-induced artifacts were successfully isolated by ICA, Figure S2 presents representative EMS-related components from a selected participant. These visualizations highlight the characteristic structure of EMS artifact components. Figure S3 presents the time-series activation of an EMS-related component, illustrating the burst-like patterns aligned with EMS pulses. For transparency, Supplementary Datasheet 2 provides ICA component information. Owing to file size constraints, only a subset of ICA component maps, spectra, and time courses is included as images. However, CSV files are provided for all components, indicating which components were rejected in each block, and an additional CSV file documents the ICA component rejection rate for each block.

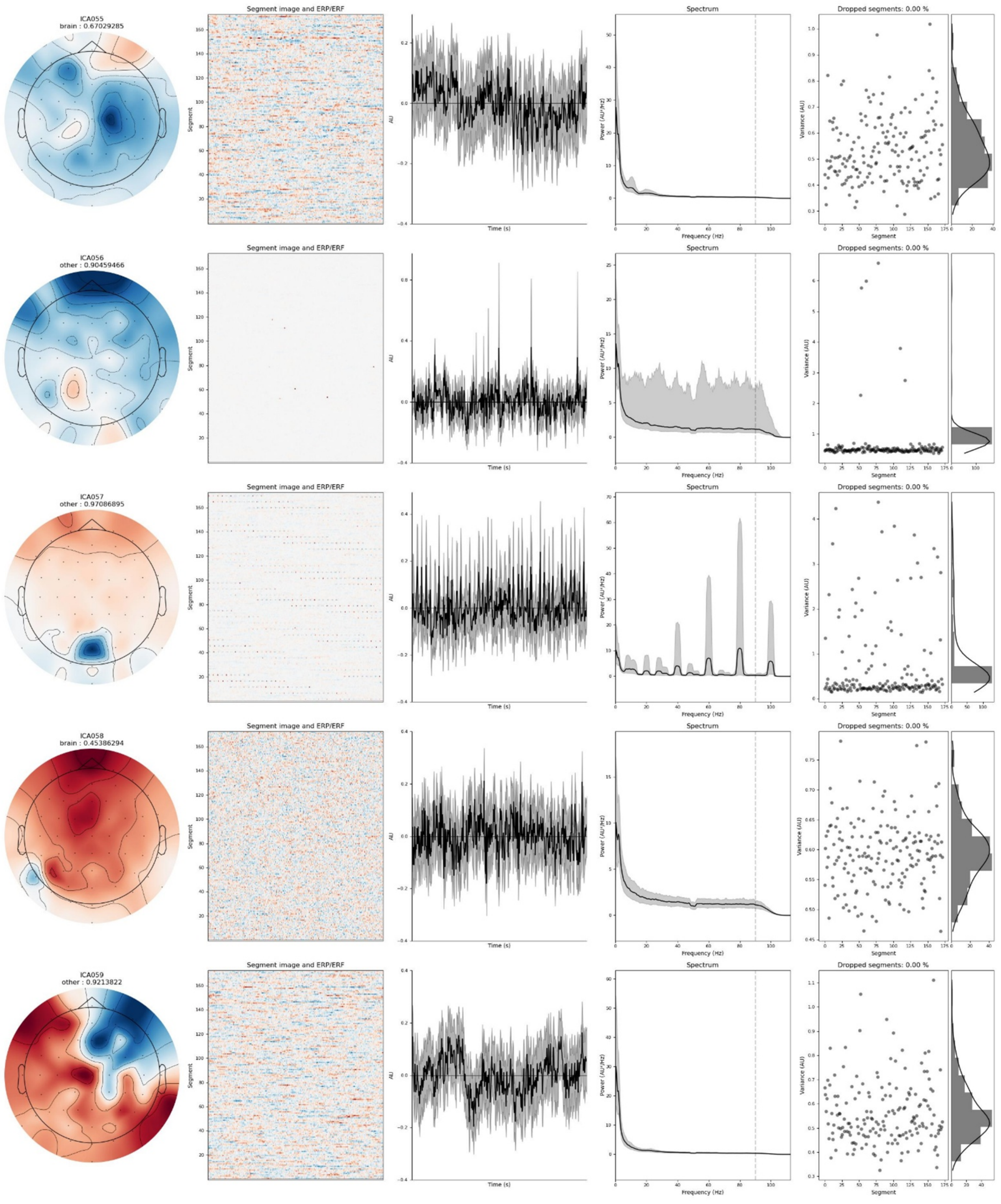

Figure S2: Representative ICA component properties generated by $mne.ica.plot_properties$ for subject 16. From top to bottom, the components are classified as kept, removed, removed, kept, and kept. For each component, the topography, the epoch image with the corresponding ERP, the component activation time course, the power spectrum, and the epoch variance are shown. The ICA57 component illustrates that EMS-induced artifacts were successfully isolated by the ICA decomposition.

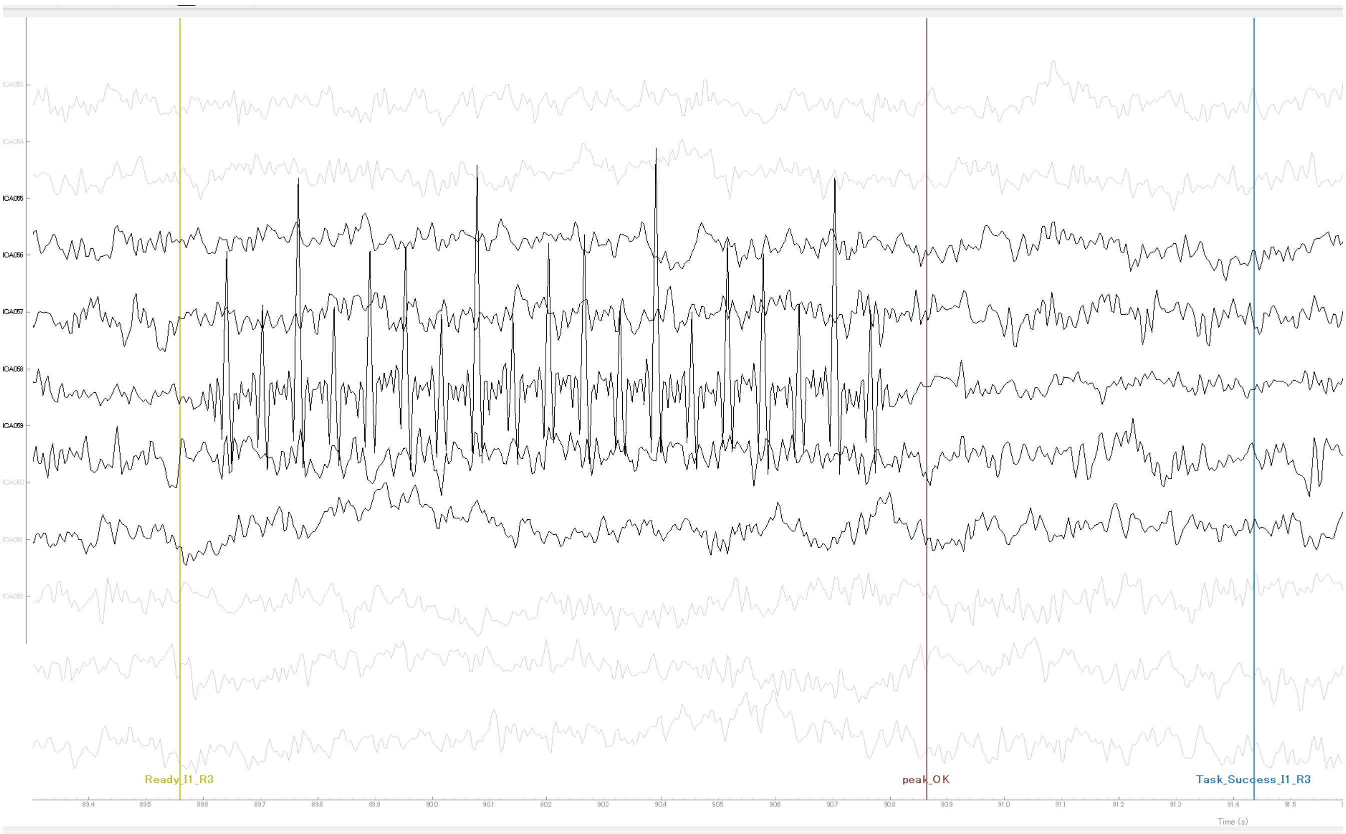

Figure S3: Time-series activation of ICA components in subject 16. The light-green markers indicate the onset of each electrical stimulation pulse. The third trace from the top (ICA57) corresponds to the EMS artifacts identified in Figure S2, exhibiting time-locked artifacts during passive, EMS-induced movements.

5 SUPPLEMENTARY RESULTS

Behavioral data

Additional behavioral data from the imagery and waiting conditions are presented in this section. Figure S4 illustrates the trajectories of wrist flexion movements induced by EMS across different target positions. Stimulation intensity was individually calibrated to produce varying levels of deviation. Figure S5 summarizes the subjective ratings of participants on the perceived incongruence between their intended and EMS-induced movements. After the wrist flexion movement, participants rated the degree of mismatch on a 7-point scale (1 = complete mismatch, 7 = complete match) via keyboard input. In the imagery session, the ratings reflected the incongruence between their imagined kinesthetic sensation and the sensation evoked by EMS. In the waiting session, participants evaluated either the kinesthetic mismatch or the physical discrepancy between the visual target and the bar. A two-way repeated-measures Analysis of Variance (ANOVA) revealed a significant main effect of error level ($F(2, 30) = 249.67, p < 0.0001$), indicating that higher error levels were associated with lower subjective ratings of movement congruence, regardless of cognitive condition. Pairwise comparisons further confirmed significant differences between all error levels ($p < 0.0001$).

Together, these results provide additional confirmation that participants were able to distinguish the magnitude of movement deviation induced by EMS.

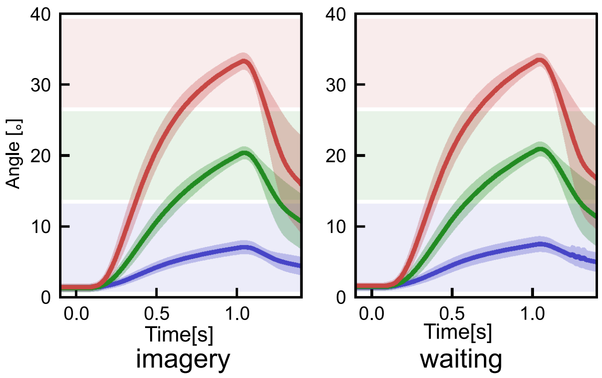

Figure S4: Mean trajectories of wrist flexion angles across two cognitive conditions (imagery and waiting). Each colored line represents one of the cognitive conditions. The shaded areas indicate the 95% confidence intervals, estimated using the t-distribution based on the mean trajectory and standard error. The three transparent squares in the background represent the target ranges for the induced movements.

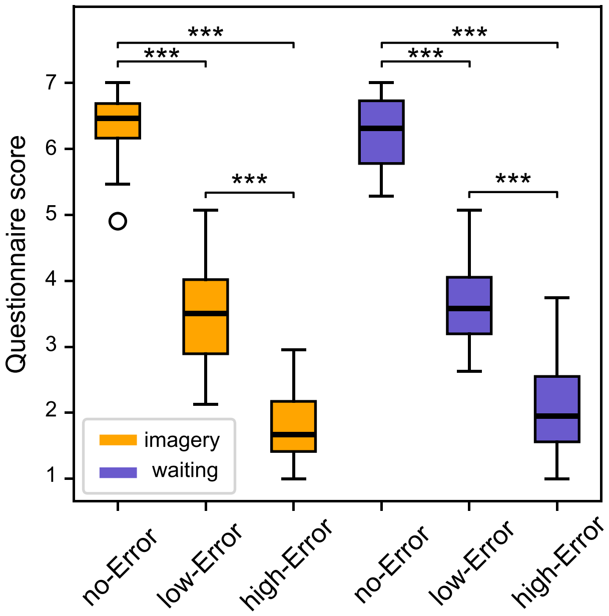

Figure S5: Box plot of subjective ratings of perceived movement congruence under the imagery session, with ratings ranging from 1 (not congruent at all) to 7 (fully congruent) for each error level (no-, low-, high-Error). A two-way repeated-measures ANOVA revealed a significant main effect of error level ($F(2, 30) = 249.67, p < 0.0001$), indicating that higher error levels were associated with lower subjective ratings of movement congruence. $***$ indicate statistically significant differences with adjusted p-values ($p < 0.0001$) based on pairwise comparisons.

EEG data

This supplementary section presents event-related potential (ERP) and time–frequency results that were investigated but not fully reported in the main text. First, Figure S6 shows grand-averaged ERP waveforms time-locked to the onset of EMS, separately for imagery and waiting conditions at channels FCz and Pz. These results were not included in the main text because no significant effects

of cognitive condition were observed. Second, Figure S7 presents ERP waveforms and corresponding scalp topographies for the All set. Although these data did not show significant effects in the statistical analyses, they are included here for completeness. The topographies revealed spatially consistent patterns for the negative ERP component (around 180 ms), the 300 ms component, and the late positive component (after 450 ms) across error conditions. The negative ERP appeared to originate from the left prefrontal area, the 300 ms component from the sensorimotor region along the midline, and the late component from both the sensorimotor and parietal regions. Third, Supplementary Table S1 summarizes the results of the cluster-based permutation tests on the time–frequency maps, including the uncorrected and corrected p-values for all significant clusters. These statistical details are provided here for completeness.

Finally, Figure S6 shows the time–frequency analysis of the all-set, revealing significant effects in both the mu- and theta-bands. Because these effects were also observed in the error-focused subset, they likely reflect error-related processes rather than stimulus-driven attention and were therefore excluded from the main text.

For the mu-band, contralateral sensorimotor electrodes (C1, C3, C5) showed a main effect of error condition ($F(2, 90) = 3.74, p = 0.027$), with lower power in the no-Error than in the high-Error condition ($p = 0.013, r = 0.26$). For the theta-band, FCz showed a main effect of cognitive condition in the all-set ($F(1, 90) = 5.22, p = 0.025$), with lower power in imagery than waiting ($p = 0.025, r = 0.23$).

Time–frequency analysis of the all-set revealed significant effects in the mu-band, confirmed by cluster-based permutation tests. The clusters emerged in time windows and topographies over the contralateral sensorimotor area that closely matched those observed in the error-focused subset, indicating that the mu-band activity reflects components differing across error conditions.

Table S1. Results of the cluster-based permutation tests.

Set	Band	Comparison	p-value	*p-value
All set	mu	high-Error − no-Error	0.0003	0.0048
	mu	low-Error − no-Error	0.002	0.028
	theta	low-Error − no-error	0.001	0.015
	theta	high-Error − no-error	0.0027	0.0351
Error-focused subset	theta	high-error − no-Error	0.0003	0.0096
	theta	low-Error − no-Error	0.0007	0.021
	mu	high-Error − no-Error	0.0003	0.0096
	mu	low-Error − no-Error	0.0013	0.0377

This table lists the significant clusters identified in the time–frequency analyses, along with the corresponding uncorrected and corrected p-values.

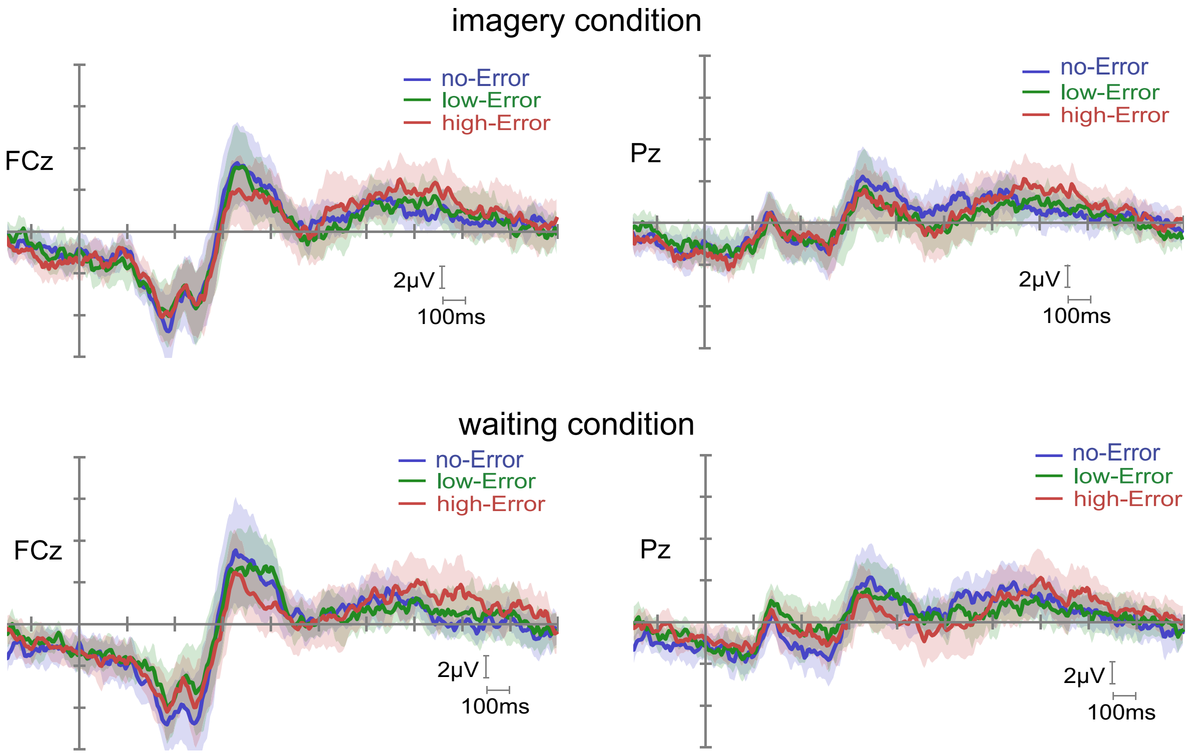

Figure S6: Grand-averaged ERP waveforms time-locked to the onset of EMS at FCz and Pz, shown separately for the imagery and waiting conditions. EEG amplitude (2 µV/div) and time (100 ms/div) are indicated on the vertical and horizontal axes, respectively. Shaded areas represent 95% confidence intervals based on the t-distribution.

6 ELECTRODE-WISE ANALYSIS OF M-BAND MODULATION

To examine the spatial characteristics of mu-band activity associated with right-hand movements in greater detail, we conducted an electrode-wise analysis on the Error-focused subset, including not only the contralateral sites (C1, C3, and C5) used in the main analysis but also medial and ipsilateral electrodes (Cz, C2, C4, and C6). As shown in Figure S8A, the spatial distribution of mu-band power exhibited a centrally peaked profile, with Cz showing the highest power and a gradual decrease toward more lateral sites. This pattern reflects the structural property that baseline mu activity is strongest over medial sensorimotor regions. In addition, Figure S8B shows the difference between no-error and high-error conditions for each electrode. Except for a relatively small difference at C6, the magnitude of error-related mu suppression was largely similar across electrodes. This pattern may suggest that error-related modulation of mu-band power is not localized to specific sites but is instead expressed broadly across the sensorimotor regions.

Taken together, these results show that although mu-band activity exhibits a sharply peaked spatial distribution centered on Cz, the suppression observed during prediction-consistent movements is broadly expressed across the sensorimotor network. This pattern may suggest that medial sensorimotor regions, including Cz, make a strong contribution to the representation of motor states that align with internal predictions, whereas error-related changes in mu power are implemented in a more spatially distributed manner.

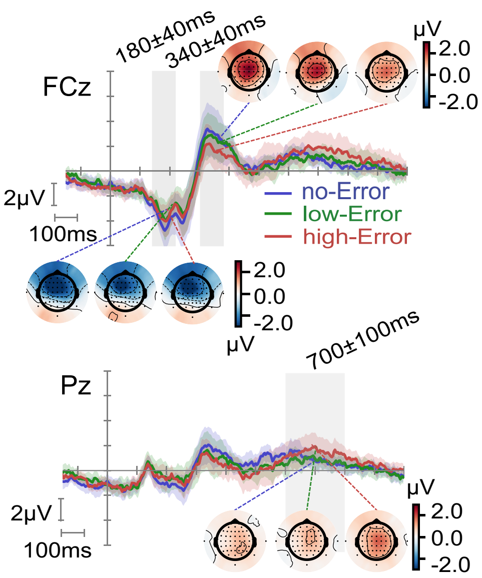

Figure S7: ERP waveforms and topographical maps for the investigated time windows are shown for each error level (All set). EEG amplitude (2 μV/div) and time (100 ms/div) are shown on the vertical and horizontal axes, respectively. Shaded areas indicate 95% confidence intervals based on the t-distribution. The plots show averages across cognitive conditions. Colored rectangles indicate the ANOVA time windows, with gray marking non-significant error-level effects.

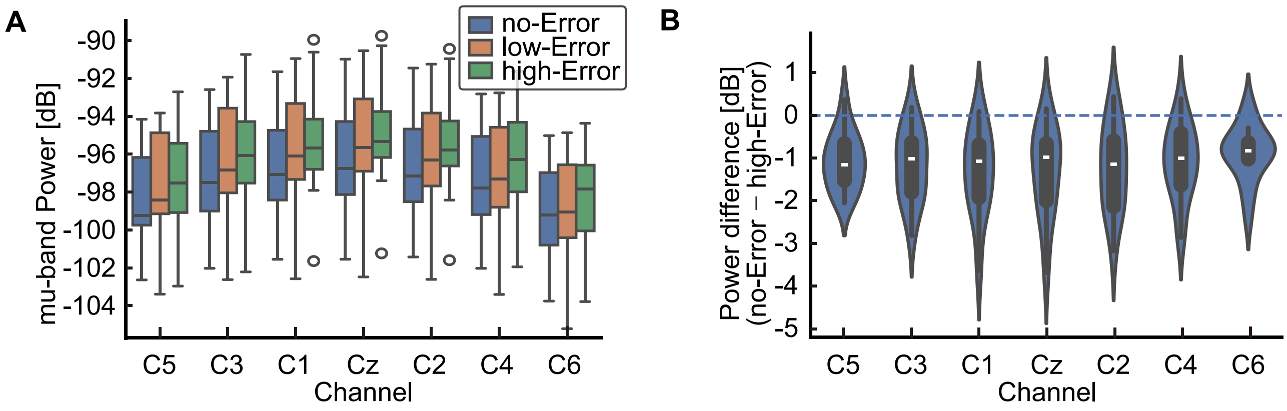

Figure S8: Electrode-wise mu-band power and error condition differences. (A) Boxplots showing mu-band power at each electrode (C1, C3, C5, Cz, C2, C4, and C6) for the no-Error, low-Error, and high-Error conditions in the Error-focused subset. (B) Violin plots illustrating the difference in mu-band power between the no-Error and high-Error conditions (no-Error – high-Error) for each electrode.

7 POST-HOC POWER ANALYSIS USING G*POWER

To assess whether the current sample size (N = 16) provided sufficient sensitivity to detect the theoretically relevant two-way interaction between error magnitude and cognitive condition,

we conducted post-hoc power analyses using G*Power. Because the main EEG analyses were performed using an ART-based non-parametric framework, conventional power analysis was not applicable. We focused this analysis on three EEG signatures—late Pe, theta power, and mu-band suppression—because these measures showed robust main effects in the main analyses and are theoretically the most relevant to the expected Error × Cognition interaction. Moreover, for the purpose of estimating statistical sensitivity, we applied a two-way repeated-measures ANOVA model to the Error-focused subset, in which stimulus-driven attention was minimized and error-related neural responses were isolated. The analysis used = 0.05, a total sample size of 16, six repeated measurements, a correlation among repeated measures of 0.50, and a nonsphericity correction of = 1. Effect sizes were calculated from the observed F-values of the two-way interaction terms, and the corresponding post-hoc power estimates are summarized in Table S2. These results indicate that the power to detect the expected interaction effects was low ($1 - \beta = 0.08$–0.28). Therefore, the absence of significant interactions in the main analyses should be interpreted cautiously, as it may reflect limited sensitivity rather than genuine null effects.

Table S2. Effect sizes and power for interaction effects.

Measure	$F(2, 75)$ (interaction)	Cohen's f	$1 - \beta$
latePe	0.81827	0.1477	0.28
theta	0.47585	0.1126	0.17
mu	0.11730	0.0559	0.08

8 SENSITIVITY ANALYSIS OF TRIAL-REJECTION THRESHOLDS

To assess whether the trial-rejection criteria influenced the EEG findings, we conducted a comprehensive sensitivity analysis across the All set, the Error-focused subset, and the Attention-focused subset. In this study, trials were considered valid only when the EMS-induced wrist movement fell within a predefined angular range around the experimenter-specified target, such that movements ending within ±6° of the intended target angle were classified as successfully reaching the target. This angular range was introduced to ensure that trials included in the analysis reflected the intended motor outcome. For each set, we repeated the two-way repeated-measures ANOVA under three target-acquisition thresholds: the standard threshold (±6°), a stricter threshold (±5°), and a more lenient threshold (±7°), allowing us to evaluate whether borderline trial selections affected the results. Table S3 lists all results with uncorrected p-values below 0.05 across all thresholds and sets. The principal findings of the study remained stable across thresholds. In the table, EEG signatures discussed as key results in the main manuscript are highlighted in yellow to indicate their relevance to the main conclusions, whereas entries showing changes in statistical significance relative to the standard ±6° threshold are marked in bold. This notation delineates the measures that formed the central results of the study and those that exhibited threshold-dependent variability. In particular, mu-band suppression over the contralateral sensorimotor cortex, theta-band enhancement at FCz, and the late positive component at Pz showed consistent effects across all thresholds. By contrast, beta-band activity and the ERN displayed threshold-dependent shifts in statistical significance. To confirm how changes in the target-acquisition threshold affected the number of retained trials, we compiled Table S4, which lists the number of valid trials obtained under each threshold (±5°, ±6°, ±7°) across all sets, and Table S5, which presents the corresponding retention rates, where the values for the standard ±6° threshold are reported as raw retention rates and the values for the

stricter (±5°) and more lenient (±7°) thresholds are expressed relative to the ±6° baseline. Together, Tables S4 and S5 indicate that, although changes in trial retention were modest for most conditions, some participants and conditions showed substantial shifts as a function of threshold choice. Overall, this sensitivity analysis suggests that the main conclusions of the study are robust to variations in the trial-exclusion threshold; however, threshold-dependent fluctuations in measures such as the ERN and FCz beta-band activity should be interpreted with caution.

Table S3. Results of the multi-angle analysis for each measure and factor.

Set	Measure	Factor	p 5°	p 6°	p 7°	$*p$ 5°	$*p$ 6°	$*p$ 7°	F 5°	F 6°	F 7°	η^2 5°	η^2 6°	η^2 7°	r 5°	r 6°	r 7°
All set	mu	error	.0003	.0001	.0001	.0022	.0007	.0007	9.02	1.42	10.45	.194	.217	.218	.440	.466	.467
	ERN	error	.0423	–	–	.2535	–	–	3.30	–	–	.081	–	–	.284	–	–
	Pe	error	–	.0434	.0305	–	.2351	.1748	–	3.27	3.66	–	.080	.089	–	.283	.298
	theta	error	–	.0392	.0291	–	.2351	.1748	–	3.38	3.71	–	.083	.090	–	.288	.300
	latePe	error	–	–	.0482	–	–	.1926	–	–	3.16	–	–	.078	–	–	.279
	beta	cognition	<.0001	<.0001	<.0001	.0003	<.0001	<.0001	19.09	27.07	25.64	.203	.265	.255	.450	.515	.505
	gamma	cognition	.0218	.0183	.0118	.1092	.0917	.0592	5.49	5.81	6.66	.068	.072	.082	.261	.268	.285
	theta	cognition	.0041	.0015	.0007	.0245	.0087	.0041	8.78	1.92	12.54	.105	.127	.143	.324	.357	.378
Error -focused subset	mu	error	<.0001	<.0001	<.0001	<.0001	<.0001	<.0001	16.25	17.50	18.60	.302	.318	.332	.550	.564	.576
	ERN	error	.0013	.0425	.0014	**.0113**	**.3401**	**.0130**	7.31	3.29	7.15	.163	.081	.160	.404	.284	.400
	beta	error	.0198	.0090	.0115	.1582	.0810	.0921	4.14	5.02	4.74	.099	.118	.112	.315	.344	.335
	theta	error	.0005	.0032	.0010	.0060	.0320	.0099	8.33	6.21	7.59	.182	.142	.168	.426	.377	.410
	latePe	error	<.0001	<.0001	<.0001	.0006	.0004	.0004	11.34	11.81	11.91	.232	.240	.241	.482	.489	.491
	ERN	cognition	.0361	–	–	–	–	–	4.56	–	–	.057	–	–	.239	–	–
	beta	cognition	.0014	.0002	.0004	.0192	.0032	.0052	11.05	15.02	13.92	.128	.167	.157	.358	.408	.396
	gamma	cognition	.0191	.0394	.0222	.2104	.4731	.2659	5.73	4.39	5.46	.071	.055	.068	.267	.235	.260
Attention -focused subset	ERN	error	.0376	–	–	.2629	–	–	3.43	–	–	.084	–	–	.289	–	–
	Pe	error	.0006	.0004	.0006	.0060	.0043	.0070	8.21	8.73	8.13	.180	.189	.178	.424	.434	.422
	gamma	error	–	–	.0484	–	–	.3388	–	–	3.15	–	–	.078	–	–	.279
	latePe	error	<.0001	<.0001	<.0001	<.0001	<.0001	<.0001	22.75	23.89	25.77	.378	.389	.407	.614	.624	.638
	ERN	cognition	–	–	.0388	–	–	.4270	–	–	4.42	–	–	.056	–	–	.236
	beta	cognition	.0055	.0016	.0018	**.0713**	**.0209**	**.0236**	8.18	1.72	10.46	.098	.125	.122	.314	.354	.350

This table lists all effects with uncorrected p-values below 0.05 across the three target-acquisition thresholds (±5°, ±6°, and ±7°) for the All set, Error-focused subset, and Attention-focused subset. EEG signatures highlighted in yellow correspond to the key measures emphasized in the main text, whereas boldface entries indicate results that changed in statistical significance relative to the standard ±6° threshold.

9 SENSITIVITY ANALYSIS USING BALANCED-TRIAL SUBSAMPLES IN ERROR-FOCUSED SUBSET

In the Error-focused subset, an unbalanced number of trials may introduce differences in signal-to-noise ratio (S/N), which may in turn bias ERP or time–frequency estimates. To ensure that the main findings were not influenced by such potential S/N discrepancies, we conducted a sensitivity analysis using balanced-trial subsampling. For each participant, error level, and cognitive condition, we identified the minimum number of valid trials available across conditions. Using this minimum as an upper bound, we randomly subsampled trials for each condition to create balanced subsets. This procedure was repeated 20 times, each iteration using a different random seed. For every subsample, ERPs (e.g., late Pe) and time–frequency measures (mu and theta power) were recomputed following the same preprocessing and analysis pipeline as in the Error-focused analysis. Within each subsample, statistical testing followed the same multiple-comparison procedure (Holm correction) used in the main analysis, and thus employed the same family size (m = 14 comparisons). However, the p-values from the corresponding Attention-focused measures were included only to maintain the original comparison family and are not analyzed or reported here. Detailed statistics for each iteration are provided in Supplementary Datasheet 3 (CSV file). For each measure, we counted the number of subsamples (out of 20) that yielded significant results after Holm correction. This

Table S4. Number of trials for each subject, session, condition, and target angle.

		All set																	
								Attention-focused subset											
		Error-focused subset																	
ID	session	highErr lowAtt			lowErr lowAtt			noErr lowAtt			noErr medAtt			noErr highAtt			lowErr medAtt		
		5°	6°	7°	5°	6°	7°	5°	6°	7°	5°	6°	7°	5°	6°	7°	5°	6°	7°
01	imagery	36	39	42	19	20	20	12	14	15	12	13	13	8	8	10	12	12	12
01	waiting	38	40	40	18	18	18	12	14	14	11	13	13	10	11	13	15	18	19
02	imagery	47	48	48	24	24	24	16	16	16	9	12	12	11	11	11	15	18	18
02	waiting	46	47	47	24	24	24	15	15	16	12	13	14	9	12	12	18	20	20
03	imagery	32	42	44	16	20	21	12	14	14	10	13	14	6	7	10	12	13	14
03	waiting	37	44	46	19	23	23	16	16	16	7	9	9	7	8	10	4	5	5
04	imagery	43	46	48	19	23	24	15	15	16	13	15	15	10	12	13	19	20	21
04	waiting	47	47	47	24	24	24	14	16	16	11	11	13	14	14	14	17	17	17
05	imagery	24	25	26	16	16	17	12	12	13	13	14	15	12	13	13	13	14	14
05	waiting	23	30	32	12	12	14	10	10	10	11	12	12	13	14	14	15	15	16
06	imagery	39	40	40	18	18	18	10	10	12	9	11	12	4	5	7	15	16	16
06	waiting	30	36	39	14	17	17	11	11	11	9	9	11	2	2	2	11	12	15
07	imagery	39	40	41	18	20	22	15	15	15	5	8	9	8	11	14	7	11	11
07	waiting	38	39	40	22	22	22	16	16	16	8	9	11	4	7	8	9	11	14
08	imagery	43	43	43	21	21	21	15	15	15	14	15	15	11	13	14	10	14	16
08	waiting	43	43	44	20	21	21	14	14	14	12	13	13	9	11	12	14	15	18
09	imagery	45	48	48	19	22	23	15	15	15	16	16	16	15	16	16	17	19	20
09	waiting	30	31	32	17	20	21	12	13	14	13	15	16	12	16	16	16	18	20
10	imagery	41	42	42	14	14	14	14	14	14	12	12	12	10	10	10	11	13	13
10	waiting	37	39	41	19	19	19	15	15	15	9	12	13	6	6	9	9	11	11
11	imagery	39	42	43	17	18	20	15	16	16	13	13	13	9	12	13	11	12	13
11	waiting	38	39	43	18	19	21	14	14	15	9	10	11	4	6	7	10	10	12
12	imagery	45	45	46	23	23	23	16	16	16	9	11	11	15	16	16	14	15	15
12	waiting	29	35	36	15	18	19	11	13	14	6	7	10	16	16	16	12	13	15
13	imagery	41	44	44	22	22	22	14	15	16	15	16	16	15	15	16	21	23	23
13	waiting	40	43	44	21	23	24	13	14	15	15	16	16	12	12	14	16	19	22
14	imagery	45	46	46	20	23	24	15	15	15	16	16	16	16	16	16	21	23	23
14	waiting	33	42	43	16	19	22	10	15	16	13	14	16	15	16	16	16	22	24
15	imagery	22	28	34	18	21	21	13	15	15	13	14	16	13	14	15	21	23	24
15	waiting	35	39	41	17	19	20	11	14	15	9	12	16	15	16	16	16	19	23
16	imagery	43	47	47	20	22	23	16	16	16	14	16	16	13	15	16	19	20	22
16	waiting	37	40	44	16	17	21	9	13	15	13	14	16	16	16	16	21	21	23

This table reports the number of valid trials obtained under the stricter (±5°), standard (±6°,), and more lenient (±7°,) thresholds for the All set, Error-focused subset, and Attention-focused subset.

provided a direct evaluation of the stability of the main Error-focused findings under balanced-trial conditions. As a result, five measures showed at least one significant effect (after correction) across the subsamples: late Pe, mu-band, beta-band, theta-band, and ERN. Late Pe amplitude was reproduced in 19 of the 20 iterations (95%), and mu-band suppression was reproduced in all iterations (100%). In contrast, beta-band, theta-band, and ERN effects reappeared in 50%, 5%, and 5% of the subsamples, respectively. These results indicate that the main effects in the Error-focused subset—particularly late Pe and mu-band modulation—stay robust under balanced-trial conditions and are not attributable to the initial unbalance in trial counts. In contrast, theta-, beta-band, and ERN effects showed substantially lower stability across subsampling iterations, suggesting that these measures are more sensitive to variability in signal-to-noise ratio and trial counts. Together, this analysis demonstrates that late Pe and mu-band oscillations are the most reliable neural responses

Table S5. Percentage performance for each subject, session, condition, and target angle.

		All set																	
								Attention-focused subset											
		Error-focused subset																	
ID	session	highErr lowAtt			lowErr lowAtt			noErr lowAtt			noErr medAtt			noErr highAtt			lowErr medAtt		
		5°	6°	7°	5°	6°	7°	5°	6°	7°	5°	6°	7°	5°	6°	7°	5°	6°	7°
01	imagery	-6.3	81.3	6.3	-4.2	83.3	0.0	-12.5	87.5	6.3	-6.3	81.3	0.0	0.0	50.0	12.5	0.0	50.0	0.0
01	waiting	-4.2	83.3	0.0	0.0	75.0	0.0	-12.5	87.5	0.0	-12.5	81.3	0.0	-6.3	68.8	12.5	-12.5	75.0	4.2
02	imagery	-2.1	100	0.0	0.0	100	0.0	0.0	100	0.0	-18.8	75.0	0.0	0.0	68.8	0.0	-12.5	75.0	0.0
02	waiting	-2.1	97.9	0.0	0.0	100	0.0	0.0	93.8	6.3	-6.3	81.3	6.3	-18.8	75.0	0.0	-8.3	83.3	0.0
03	imagery	-20.8	87.5	4.2	-16.7	83.3	4.2	-12.5	87.5	0.0	-18.8	81.3	6.3	-6.3	43.8	18.8	-4.2	54.2	4.2
03	waiting	-14.6	91.7	4.2	-16.7	95.8	0.0	0.0	100	0.0	-12.5	56.3	0.0	-6.3	50.0	12.5	-4.2	20.8	0.0
04	imagery	-6.3	95.8	4.2	-16.7	95.8	4.2	0.0	93.8	6.3	-12.5	93.8	0.0	-12.5	75.0	6.3	-4.2	83.3	4.2
04	waiting	0.0	97.9	0.0	0.0	100	0.0	-12.5	100	0.0	0.0	68.8	12.5	0.0	87.5	0.0	0.0	70.8	0.0
05	imagery	-2.1	52.1	2.1	0.0	66.7	4.2	0.0	75.0	6.3	-6.3	87.5	6.3	-6.3	81.3	0.0	-4.2	58.3	0.0
05	waiting	-14.6	62.5	4.2	0.0	50.0	8.3	0.0	62.5	0.0	-6.3	75.0	0.0	-6.3	87.5	0.0	0.0	62.5	4.2
06	imagery	-2.1	83.3	0.0	0.0	75.0	0.0	0.0	62.5	12.5	-12.5	68.8	6.3	-6.3	31.3	12.5	-4.2	66.7	0.0
06	waiting	-12.5	75.0	6.3	-12.5	70.8	0.0	0.0	68.8	0.0	0.0	56.3	12.5	0.0	12.5	0.0	-4.2	50.0	12.5
07	imagery	-2.1	83.3	2.1	-8.3	83.3	8.3	0.0	93.8	0.0	-18.8	50.0	6.3	-18.8	68.8	18.8	-16.7	45.8	0.0
07	waiting	-2.1	81.3	2.1	0.0	91.7	0.0	0.0	100	0.0	-6.3	56.3	12.5	-18.8	43.8	6.3	-8.3	45.8	12.5
08	imagery	0.0	89.6	0.0	0.0	87.5	0.0	0.0	93.8	0.0	-6.3	93.8	0.0	-12.5	81.3	6.3	-16.7	58.3	8.3
08	waiting	0.0	89.6	2.1	-4.2	87.5	0.0	0.0	87.5	0.0	-6.3	81.3	0.0	-12.5	68.8	6.3	-4.2	62.5	12.5
09	imagery	-6.3	100	0.0	-12.5	91.7	4.2	0.0	93.8	0.0	0.0	100	0.0	-6.3	100	0.0	-8.3	79.2	4.2
09	waiting	-2.1	64.6	2.1	-12.5	83.3	4.2	-6.3	81.3	6.3	-12.5	93.8	6.3	-25.0	100	0.0	-8.3	75.0	8.3
10	imagery	-2.1	87.5	0.0	0.0	58.3	0.0	0.0	87.5	0.0	0.0	75.0	0.0	0.0	62.5	0.0	-8.3	54.2	0.0
10	waiting	-4.2	81.3	4.2	0.0	79.2	0.0	0.0	93.8	0.0	-18.8	75.0	6.3	0.0	37.5	18.8	-8.3	45.8	0.0
11	imagery	-6.3	87.5	2.1	-4.2	75.0	8.3	-6.3	100	0.0	0.0	81.3	0.0	-18.8	75.0	6.3	-4.2	50.0	4.2
11	waiting	-2.1	81.3	8.3	-4.2	79.2	8.3	0.0	87.5	6.3	-6.3	62.5	6.3	-12.5	37.5	6.3	0.0	41.7	8.3
12	imagery	0.0	93.8	2.1	0.0	95.8	0.0	0.0	100	0.0	-12.5	68.8	0.0	-6.3	100	0.0	-4.2	62.5	0.0
12	waiting	-12.5	72.9	2.1	-12.5	75.0	4.2	-12.5	81.3	6.3	-6.3	43.8	18.8	0.0	100	0.0	-4.2	54.2	8.3
13	imagery	-6.3	91.7	0.0	0.0	91.7	0.0	-6.3	93.8	6.3	-6.3	100	0.0	0.0	93.8	6.3	-8.3	95.8	0.0
13	waiting	-6.3	89.6	2.1	-8.3	95.8	4.2	-6.3	87.5	6.3	-6.3	100	0.0	0.0	75.0	12.5	-12.5	79.2	12.5
14	imagery	-2.1	95.8	0.0	-12.5	95.8	4.2	0.0	93.8	0.0	0.0	100	0.0	0.0	100	0.0	-8.3	95.8	0.0
14	waiting	-18.8	87.5	2.1	-12.5	79.2	12.5	-31.3	93.8	6.3	-6.3	87.5	12.5	-6.3	100	0.0	-25.0	91.7	8.3
15	imagery	-12.5	58.3	12.5	-12.5	87.5	0.0	-12.5	93.8	0.0	-6.3	87.5	12.5	-6.3	87.5	6.3	-8.3	95.8	4.2
15	waiting	-8.3	81.3	4.2	-8.3	79.2	4.2	-18.8	87.5	6.3	-18.8	75.0	25.0	-6.3	100	0.0	-12.5	79.2	16.7
16	imagery	-8.3	97.9	0.0	-8.3	91.7	4.2	0.0	100	0.0	-12.5	100	0.0	-12.5	93.8	6.3	-4.2	83.3	8.3
16	waiting	-6.3	83.3	8.3	-4.2	70.8	16.7	-25.0	81.3	12.5	-6.3	87.5	12.5	0.0	100	0.0	0.0	87.5	8.3

This table summarizes the retention rates under each target-acquisition threshold. Values for the standard ±6°) threshold are presented as raw retention rates, whereas values for the ±5°) and ±7°) thresholds are expressed relative to the ±6°) baseline to illustrate threshold-dependent increases or decreases in retained trials.

in the Error-focused subset, whereas theta-, beta-band, and ERN effects should be interpreted with greater caution due to their reduced reproducibility.

10 ADDITIONAL ANALYSIS

Previous studies have demonstrated that decreases in /-band activity (ERD) during the preparation and imagery of right-hand movements reflect both individual motor expertise and task complexity. In particular, experts exhibit more stably suppressed activity in the primary motor and premotor cortices, while showing greater sensitivity of /-band ERD to task complexity compared to nonexperts (Wolf et al., 2014; Zabielska-Mendyk et al., 2018; Percio et al., 2010). Kilteni et al. further demonstrated that motor imagery continuously generates forward sensory predictions through the forward model (Kilteni et al., 2018). Within the framework of predictive coding, such predictions are

constantly compared with actual sensory inputs, and any discrepancies are encoded as prediction errors (Friston, 2005, 2010; Rao and Ballard, 1999). Accordingly, in the present task, we predicted that the more clearly an internal prediction is formed through motor imagery prior to passive movement, the more likely any mismatch with the actual input would manifest as a neural response and be reflected in error-related neural markers such as the late positive component. Based on this rationale, we formulated the following hypotheses: (1) motor imagery enhances the stability of error-related neural markers, and (2) the expression of these markers is modulated by inter-individual differences in sensorimotor representations as reflected in mu-band activity.

10.1 Late Positive Component Variability

To test our first hypothesis (1), that motor imagery enhances the stability of error-related neural markers, we examined whether variability in the late positive component differed between cognitive conditions (imagery vs. waiting). To account for the possibility that variability estimates may be reduced when using mean amplitudes of the late positive component, we extracted trial-by-trial mean amplitude values from each participant. These mean amplitudes were identified at the Pz electrode using the same 200-ms window (600–800 ms) employed in the main text. The variability of these mean amplitudes was quantified as the within-subject standard deviation across trials for each error condition level and cognitive condition. These variability measures were then submitted to a two-way repeated-measures ANOVA using the aligned rank transform (ART) procedure, with error condition (no-, low-, high-Error) and cognitive condition (imagery, waiting) as within-subject factors. A significant main effect of cognitive condition was observed, $F(1, 75) = 5.78, p = 0.019$, with lower variability in the imagery condition compared to the waiting condition (Figure S9). These results suggest that motor imagery reduced the variability of late positive component amplitudes, indicating a more stable neural response to errors when participants engaged in motor imagery. This stabilization may reflect enhanced reliance on internal sensorimotor representations during imagery, which provides a more consistent frame of reference for evaluating motor errors. In contrast, under the waiting condition, the absence of active sensorimotor engagement may lead to greater variability in error-related processing. The present results support our hypothesis that motor imagery enhances the stability of error-related neural responses. Under the imagery condition, variability in the late positive component was reduced, indicating that neural responses to errors emerged more consistently. From the perspective of predictive coding, this stabilization can be interpreted as a consequence of internally generated predictions during imagery functioning as a reference frame, thereby enabling a consistent comparison with sensory inputs. In contrast, under the waiting condition, no active sensorimotor representation was formed, which may have led to more variable error processing. Thus, motor imagery appears to stabilize the neural basis of error processing and provides important insights into the mechanisms by which expertise and training enhance the efficiency of error-related neural responses. At the same time, it should be noted that the present analyses focused on peak amplitudes within a predefined latency window. While this approach captures trial-to-trial fluctuations in the canonical late positive component response, it may also be sensitive to noise inherent in single-trial data. Future studies could compare peak- and mean-based measures, or combine EEG with complementary modalities (e.g., fMRI, behavioral performance indices), to further validate the observed stabilization effect.

The present results support our hypothesis that motor imagery enhances the stability of error-related neural markers. Under the imagery condition, variability in the late positive component was reduced, indicating that neural responses to errors emerged more consistently. From the perspective

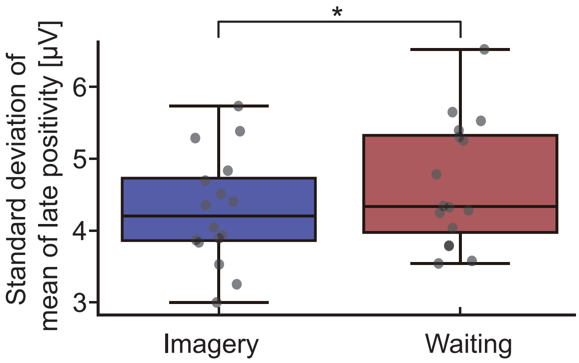

Figure S9: Boxplot of the standard deviation of late positive component peak amplitudes at Pz, shown separately for the two cognitive conditions (imagery and waiting). Values were averaged across the three error-condition levels for each subject. A significant main effect of cognitive condition was observed, $F(1, 75) = 5.78, p = 0.0189$, indicating lower variability in the imagery condition compared to the waiting condition.

of predictive coding, this stabilization can be interpreted as a consequence of internally generated predictions during imagery functioning as a reference frame, thereby enabling a consistent comparison with sensory inputs. In contrast, under the waiting condition, no active sensorimotor representation was formed, which may have led to more variable error processing. Thus, motor imagery appears to stabilize the neural basis of error processing and provides important insights into the mechanisms by which expertise and training enhance the efficiency of error-related neural responses. At the same time, it should be noted that the present analyses focused on peak amplitudes within a predefined latency window. While this approach captures trial-to-trial fluctuations in the canonical late positive component response, it may also be sensitive to noise inherent in single-trial data. Future studies could compare peak- and mean-based measures, or combine EEG with complementary modalities (e.g., fMRI, behavioral performance indices), to further validate the observed stabilization effect.

10.2 Correlation between Mu-Band Power and Late Positive Component Amplitude

To test our second hypothesis (2), we examined the relationship between oscillatory activity in the mu-band and late positivity amplitude. Trial-by-trial mu-band power (8–13 Hz) was computed using time–frequency decomposition with Morlet wavelets (ncycles = 8) implemented in MNE-Python. Power analyses focused on contralateral motor cortex electrodes (Cz, C1, C3), which showed prominent activity in Figure S8. Power was averaged across the motor imagery period from –3.0 to 0.0 s relative to EMS onset, with a baseline of –4.0 to –3.1 s. This analysis reflects between-subject correlations, assessed using Pearson's correlation coefficients, linking individual mu power during motor imagery to the corresponding late positive component amplitudes. Correlations were assessed separately for low- and high-Error condition levels under the motor imagery and waiting conditions. Late positivity amplitude was defined as the mean amplitude within 600–800 ms after EMS onset, measured from the ERP waveform. To specifically examine the relationship between motor error and the pre-movement state of motor imagery, analyses were restricted to the Error-focused subset. The rationale for this restriction is that, in the no-Error condition, the magnitude of motor imagery could still vary depending on the target position, even when no motor error was induced. Such variability would complicate the interpretation of the relationship between motor imagery and error. By excluding no-Error trials and focusing on the Error-focused subset,

we minimized this confound and isolated error-related effects more clearly. In the Error-focused subset, the waiting condition showed no significant correlations: no-Error ($r = -0.30$), low-Error ($r = 0.35$), and high-Error condition levels ($r = 0.09$). In contrast, the imagery condition revealed a consistent tendency toward negative correlations, with significant effects observed for both low-Error ($r = -0.65, p = 0.0094$) and high-Error levels ($r = -0.51, p = 0.0140$). no-Error level, however, did not show a significant correlation ($r = -0.37$). These supplementary results support our second hypothesis (2), demonstrating that the observed negative correlations were driven by systematic variations in mu-band ERD/ERS. Specifically, participants who exhibited mu activity closer to the desynchronization direction during motor imagery also showed larger late positive component amplitudes. This pattern suggests that motor imagery not only stabilizes error-related processing but also modulates its magnitude as a function of sensorimotor engagement. One limitation of the present analysis is related to the choice of baseline. The baseline period (–4.0 to –3.1 s) corresponded to a rest period in which participants were instructed to return their wrist to the original position. Thus, residual motor-related activity may have influenced the baseline, potentially biasing the ERSP results. In particular, activity that would normally be expressed as mu-band desynchronization (ERD) may instead have appeared as a relative synchronization (ERS) due to this baseline definition. Future studies should employ baseline periods recorded during true rest, without concurrent motor activity, to more accurately assess error-related oscillatory dynamics.

REFERENCES

Wolf S, Brölz E, Scholz D, Ramos-Murguialday A, Keune PM, Hautzinger M, et al. Winning the game: brain processes in expert, young elite and amateur table tennis players. Front Behav Neurosci. 8 (2014). doi:10.3389/fnbeh.2014.00370.

Zabielska-Mendyk E, Francuz P, Jaśkiewicz M, Augustynowicz P. The effects of motor expertise on sensorimotor rhythm desynchronization during execution and imagery of sequential movements. Neuroscience. 384 (2018) 101–110. doi:10.1016/j.neuroscience.2018.05.028.

Percio CD, Infarinato F, Iacoboni M, Marzano N, Soricelli A, Aschieri P, et al. Movement-related desynchronization of alpha rhythms is lower in athletes than non-athletes: A high-resolution EEG study. Clin Neurophysiol. 121 (2010) 482–491. doi:10.1016/j.clinph.2009.12.004.

Kilteni K, Andersson BJ, Houborg C, Ehrsson HH. Motor imagery involves predicting the sensory consequences of the imagined movement. Nat Commun. 9 (2018) 1617. doi:10.1038/s41467-018-03989-0.

Friston K. A theory of cortical responses. Philos Trans R Soc Lond B Biol Sci. 360 (2005) 815–836. doi:10.1098/rstb.2005.1622.

Friston K. The free-energy principle: a unified brain theory? Nat Rev Neurosci. 11 (2010) 127–138. doi:10.1038/nrn2787.

Rao RPN, Ballard DH. Predictive coding in the visual cortex: a functional interpretation of some extra-classical receptive-field effects. Nat Neurosci. 2 (1999) 79–87. doi:10.1038/4580.
