# Supplementary figures and images for: Electroencephalography signatures of motor error and stimulus-driven attention in electrical muscle stimulation-induced wrist movements under motor imagery
Source: Front Hum Neurosci. 2026 Jan 27;19:1713908. doi: 10.3389/fnhum.2025.1713908 (PMC12887888; doi:10.3389/fnhum.2025.1713908)

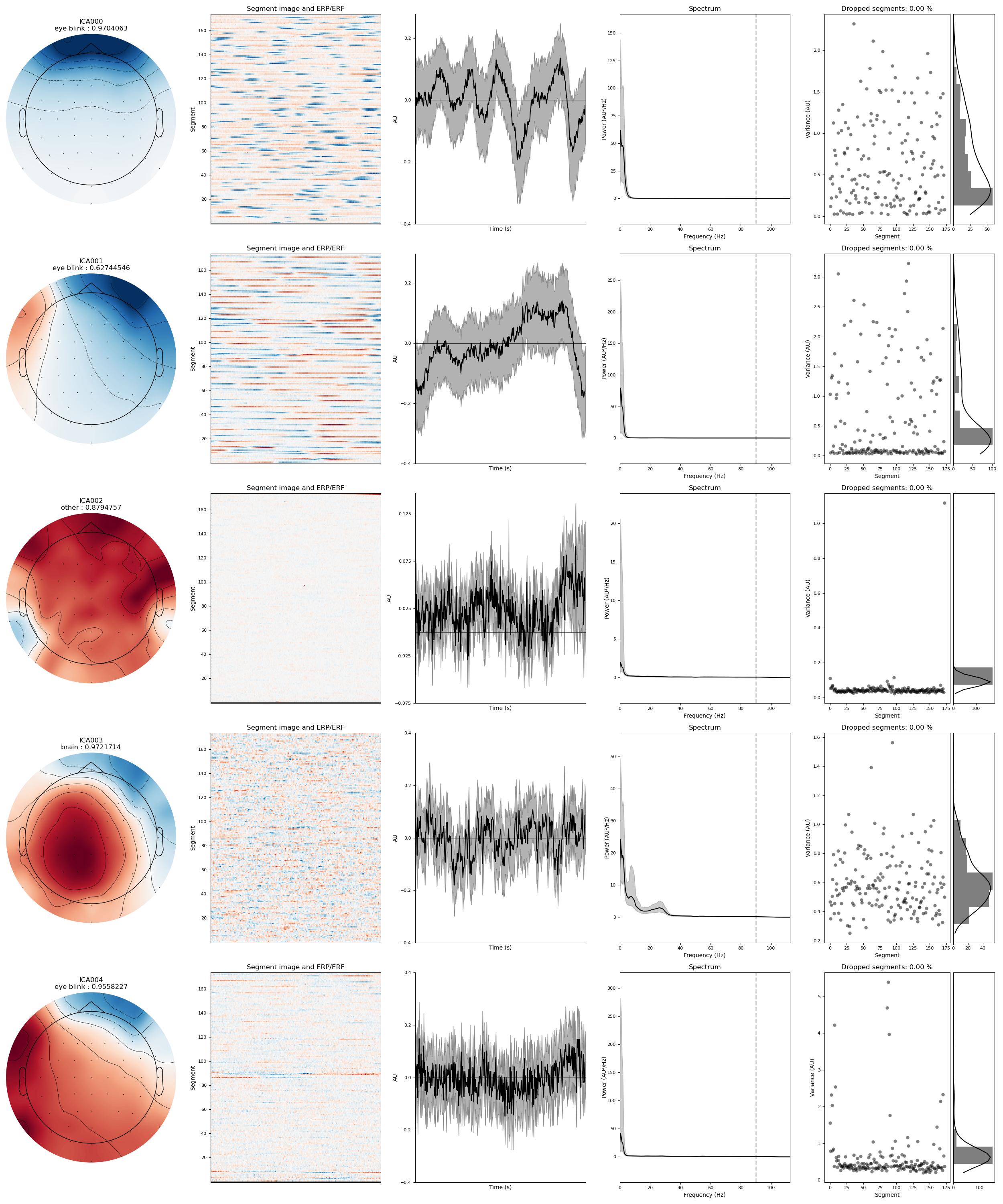

Supplement: Supplementary file 2 [file Data_Sheet_2.zip › component_image/sub02_session2_d1_block1112_0.jpg]

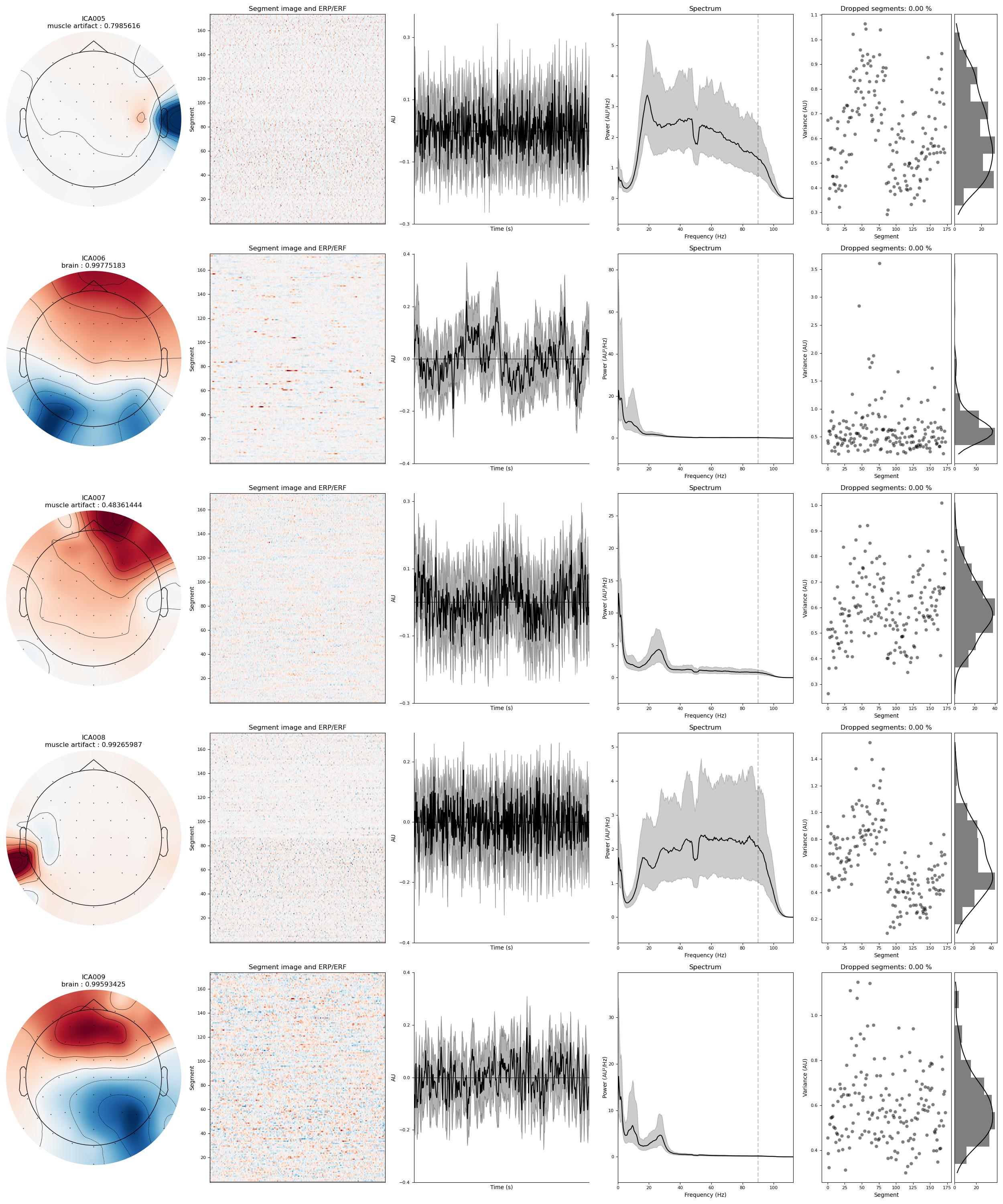

Supplement: Supplementary file 2 [file Data_Sheet_2.zip › component_image/sub02_session2_d1_block1112_1.jpg]

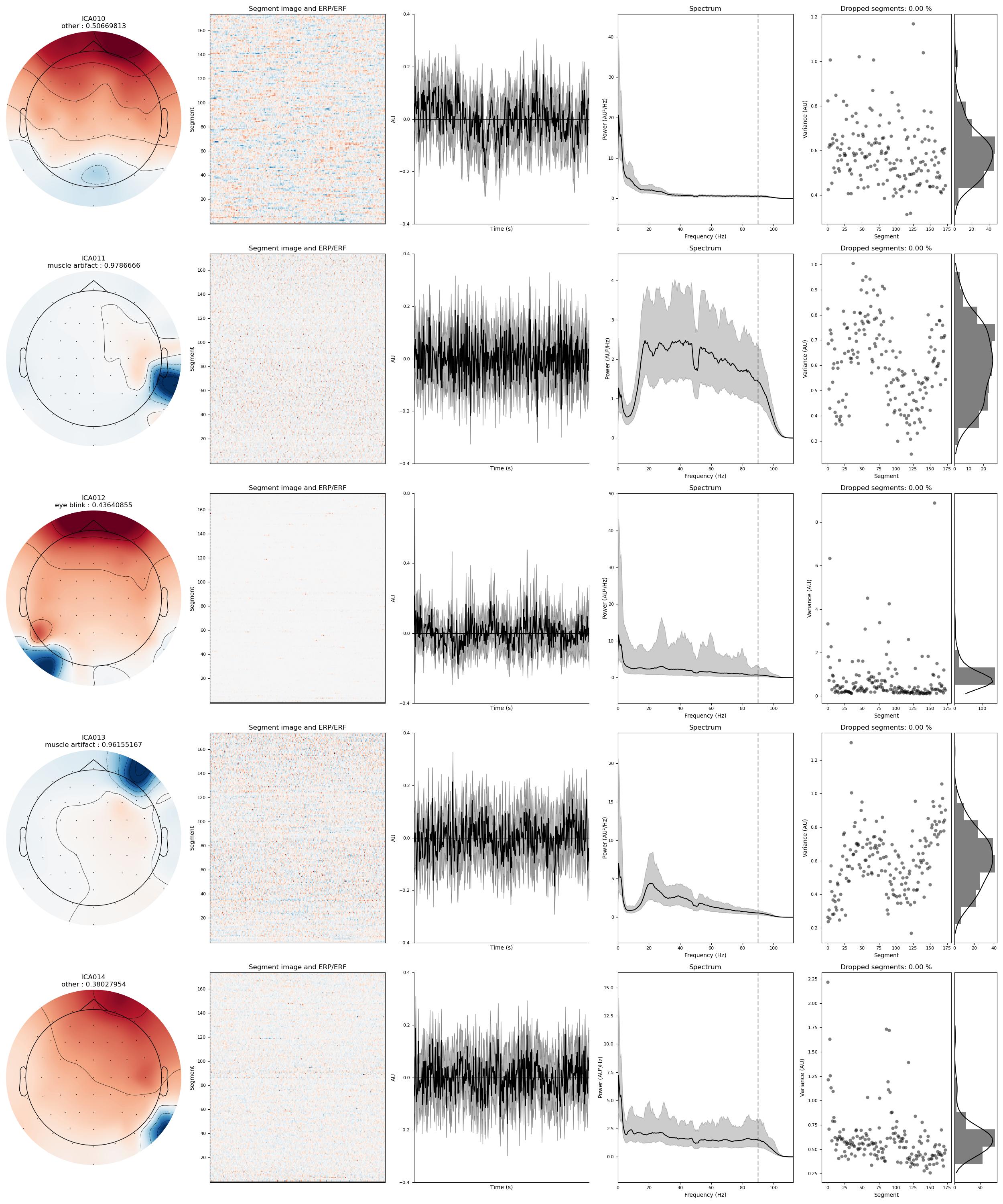

Supplement: Supplementary file 2 [file Data_Sheet_2.zip › component_image/sub02_session2_d1_block1112_2.jpg]

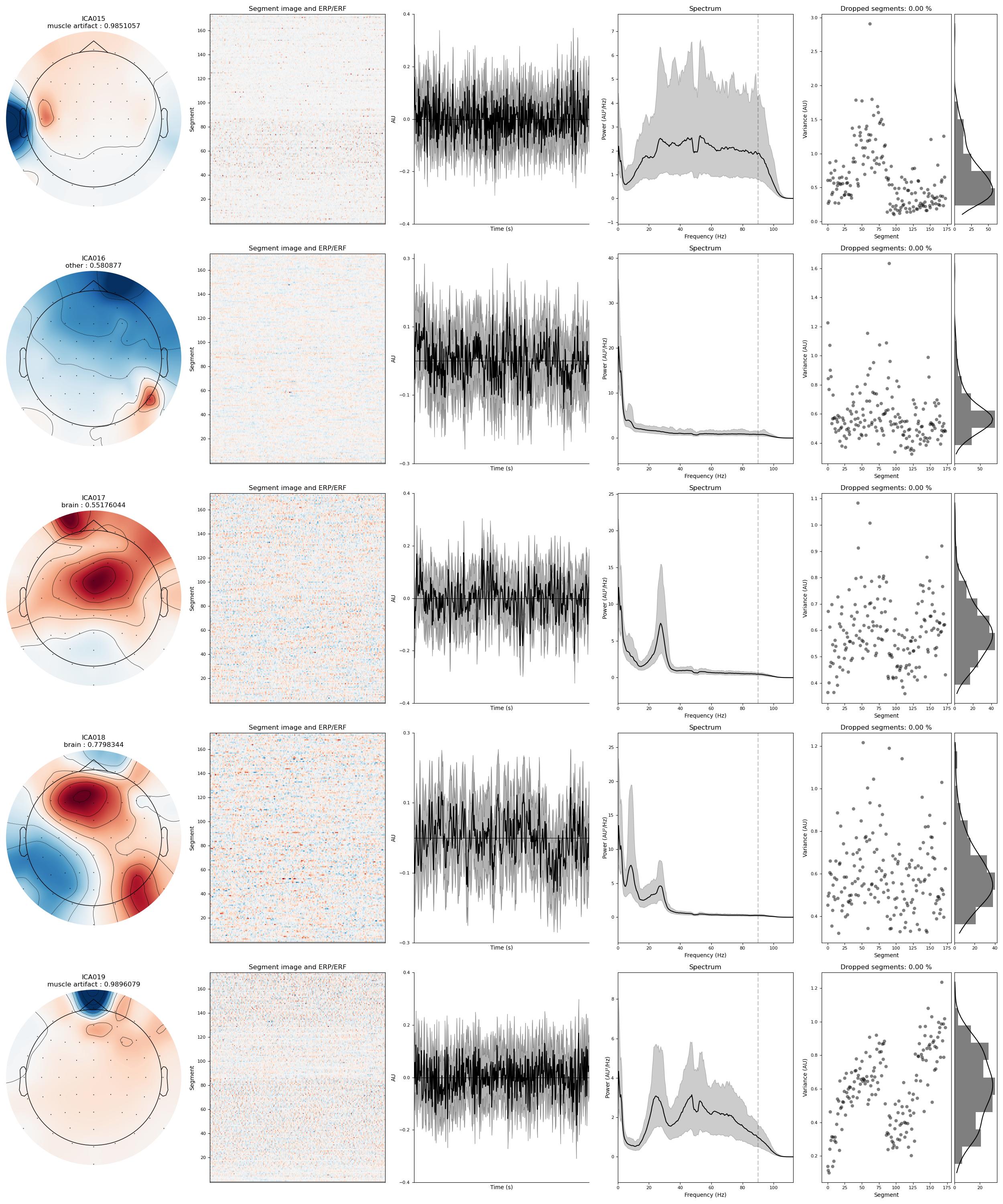

Supplement: Supplementary file 2 [file Data_Sheet_2.zip › component_image/sub02_session2_d1_block1112_3.jpg]

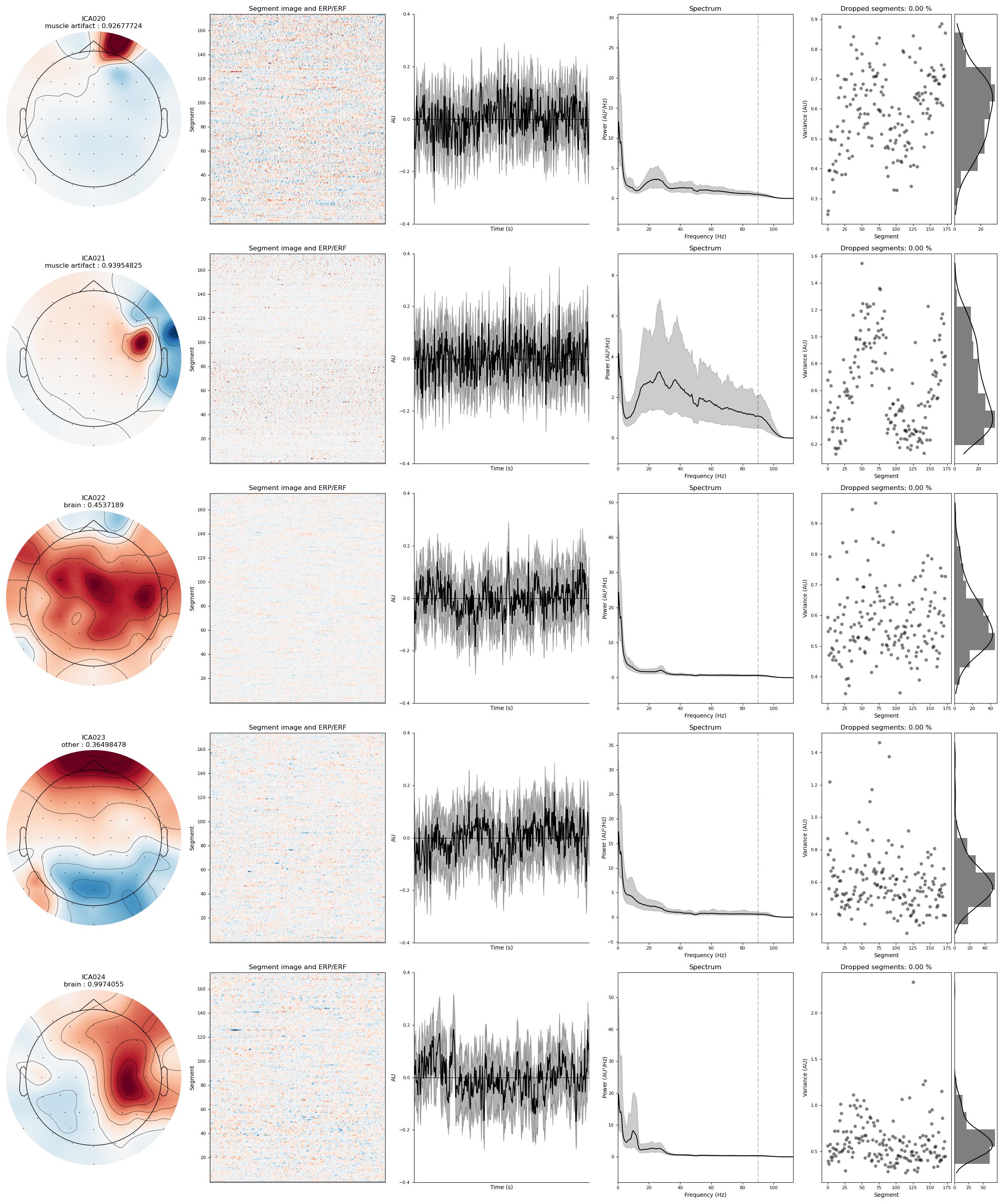

Supplement: Supplementary file 2 [file Data_Sheet_2.zip › component_image/sub02_session2_d1_block1112_4.jpg]

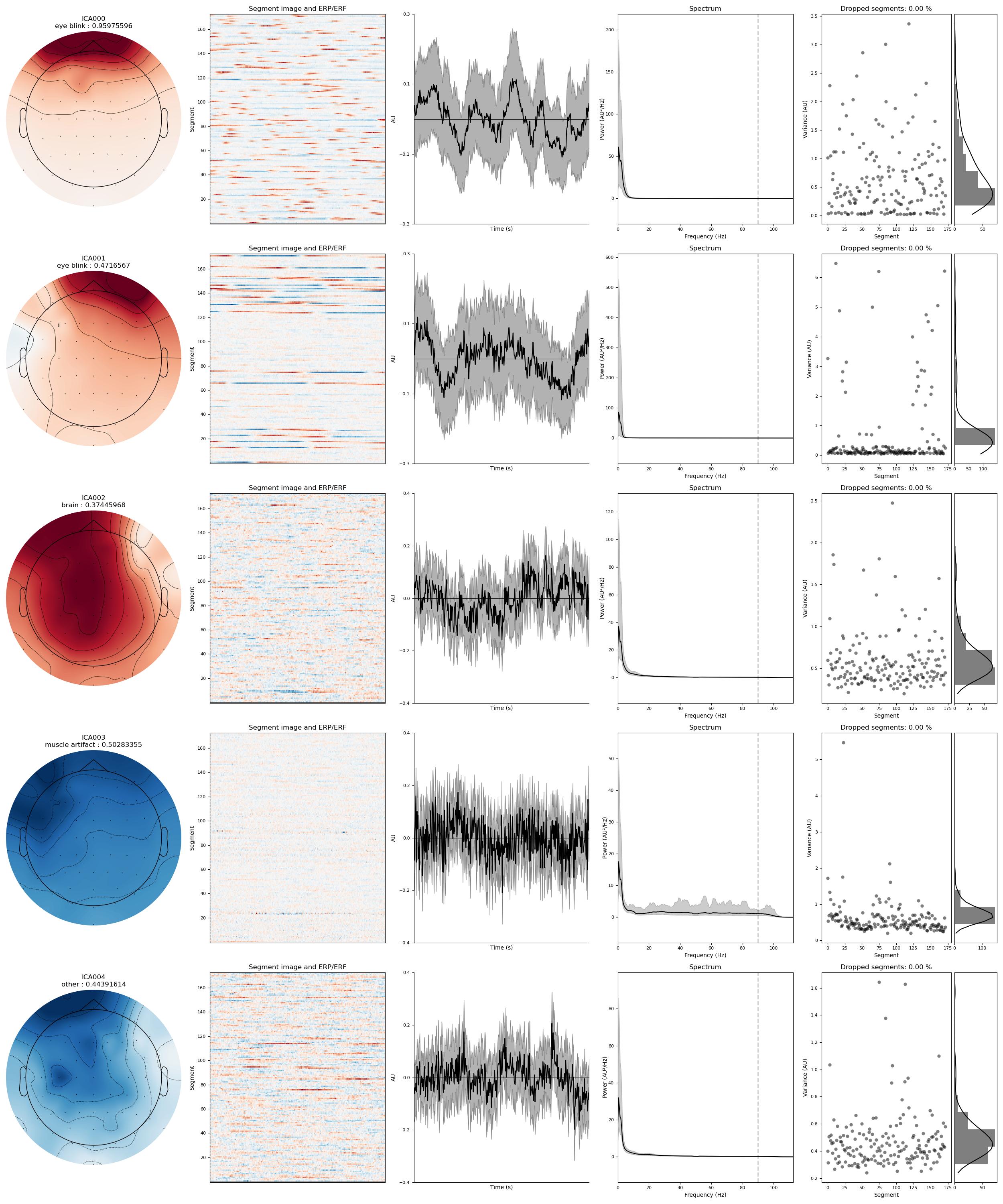

Supplement: Supplementary file 2 [file Data_Sheet_2.zip › component_image/sub04_session2_d1_block1112_0.jpg]

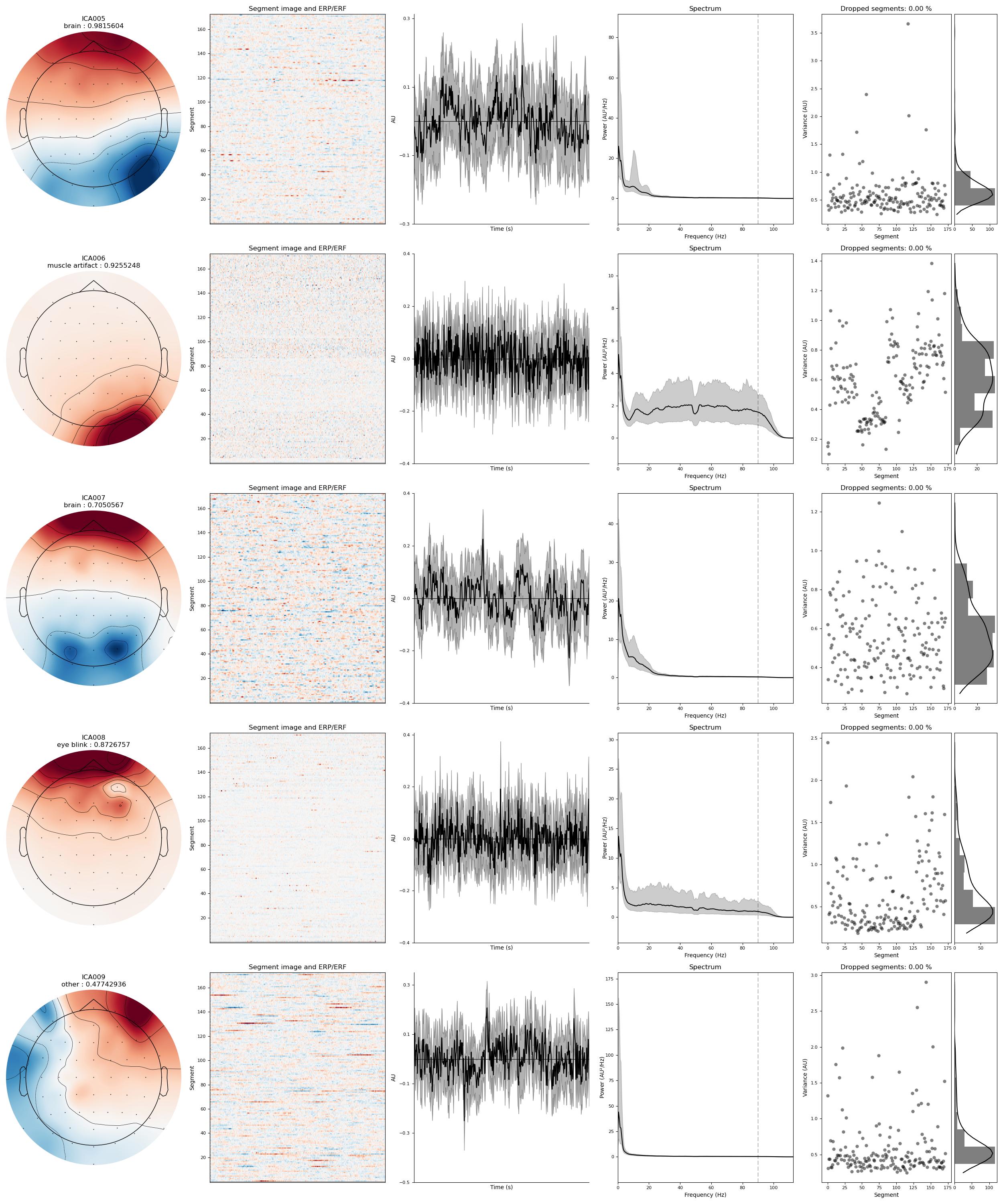

Supplement: Supplementary file 2 [file Data_Sheet_2.zip › component_image/sub04_session2_d1_block1112_1.jpg]

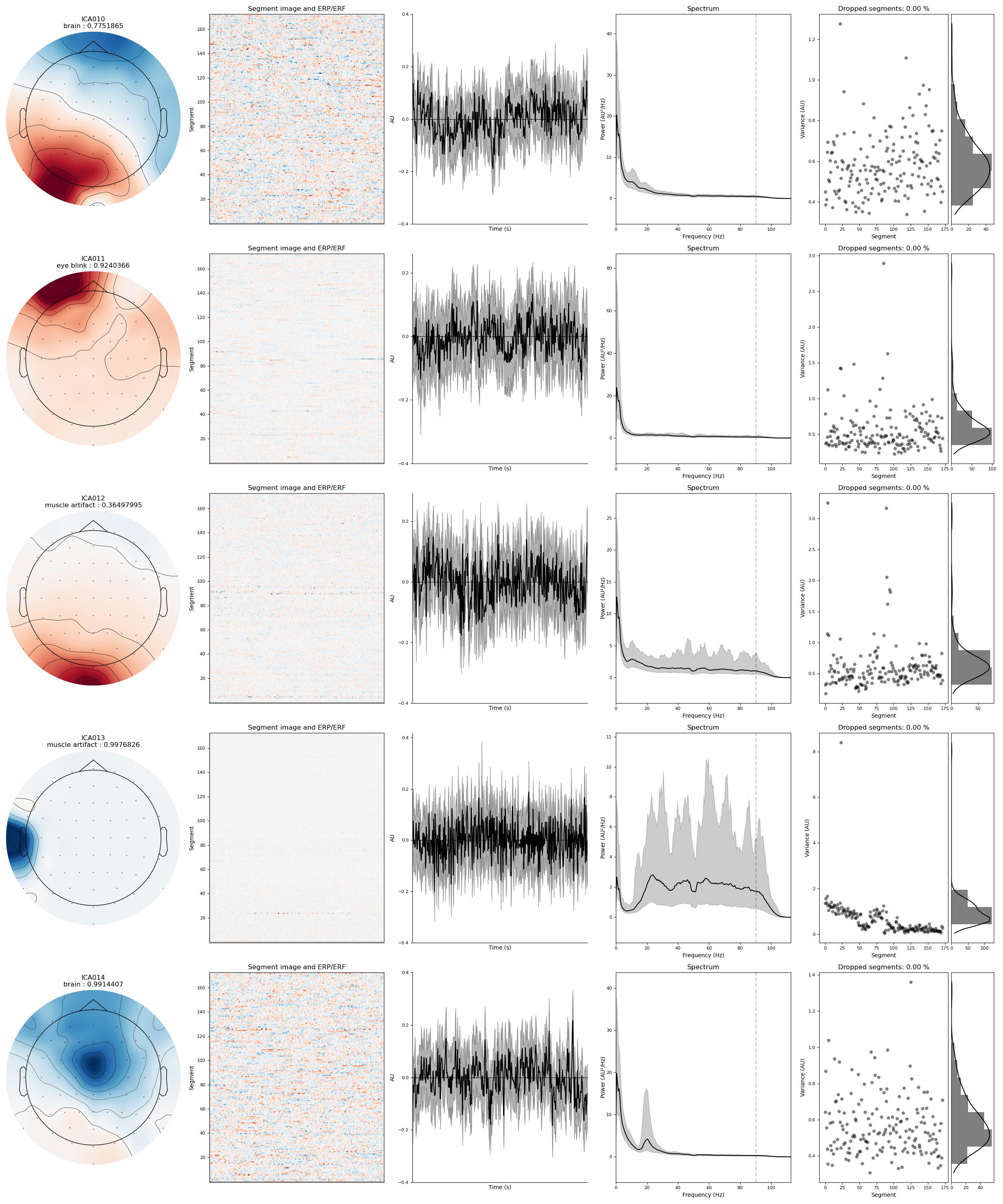

Supplement: Supplementary file 2 [file Data_Sheet_2.zip › component_image/sub04_session2_d1_block1112_2.jpg]

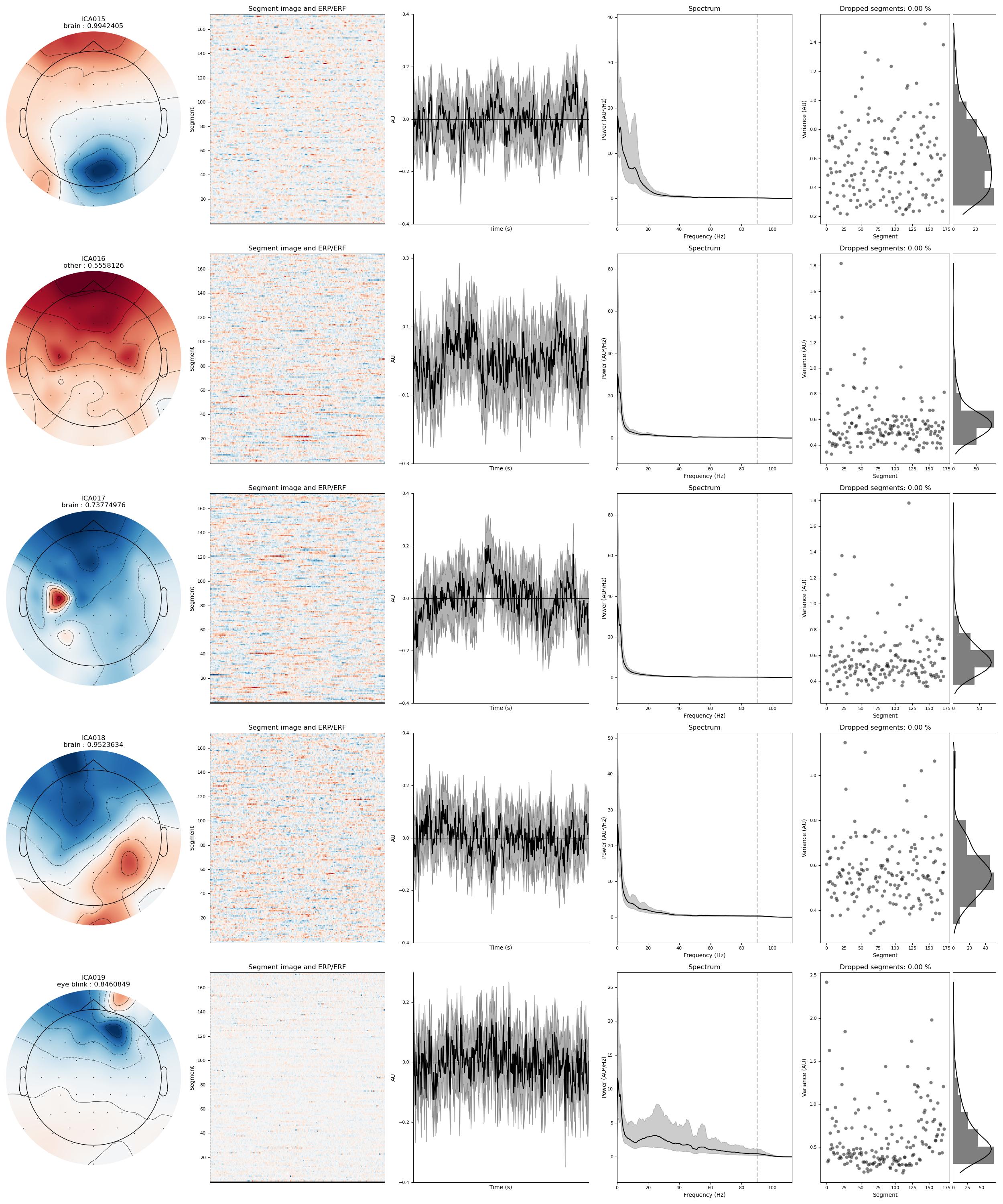

Supplement: Supplementary file 2 [file Data_Sheet_2.zip › component_image/sub04_session2_d1_block1112_3.jpg]

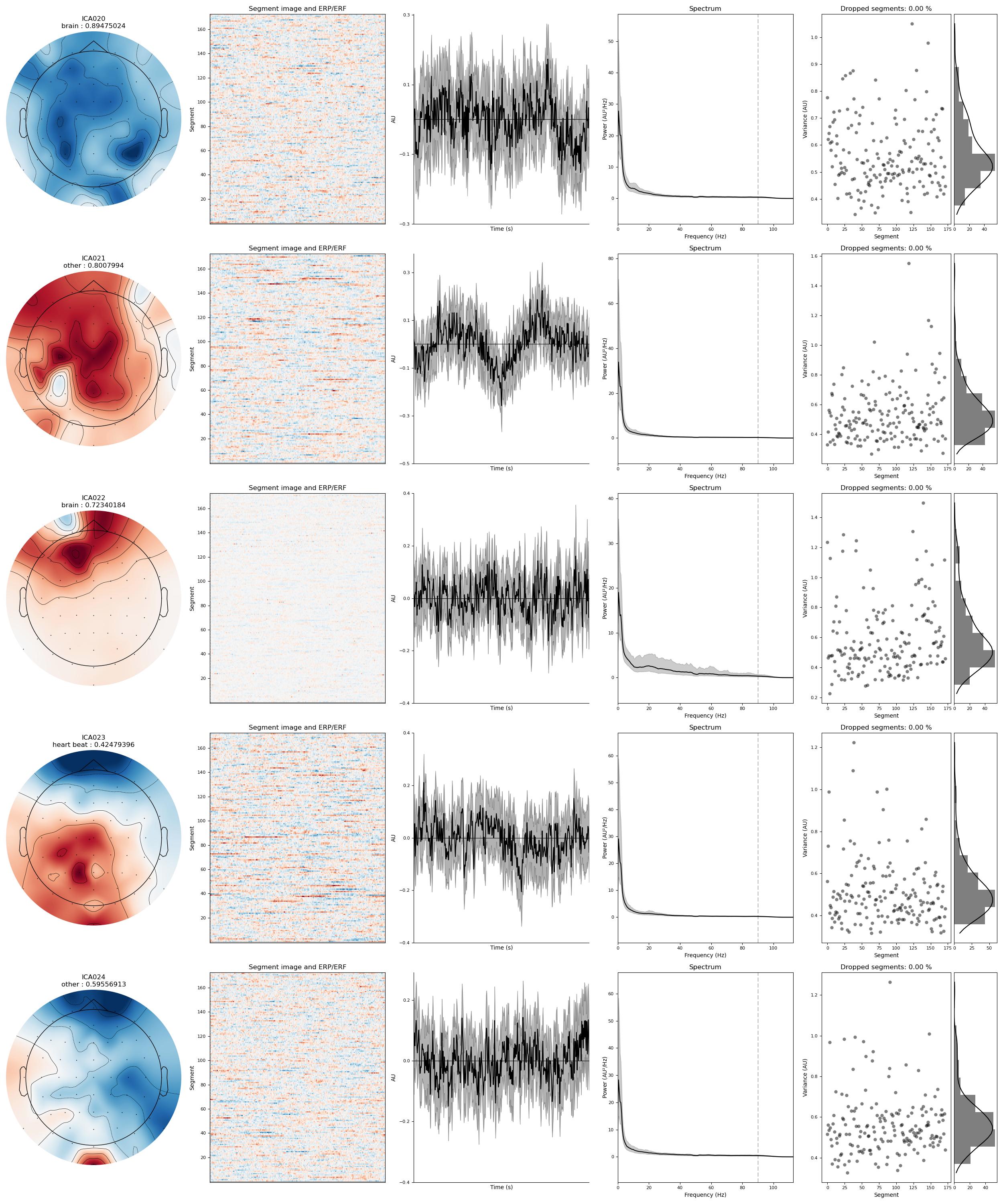

Supplement: Supplementary file 2 [file Data_Sheet_2.zip › component_image/sub04_session2_d1_block1112_4.jpg]

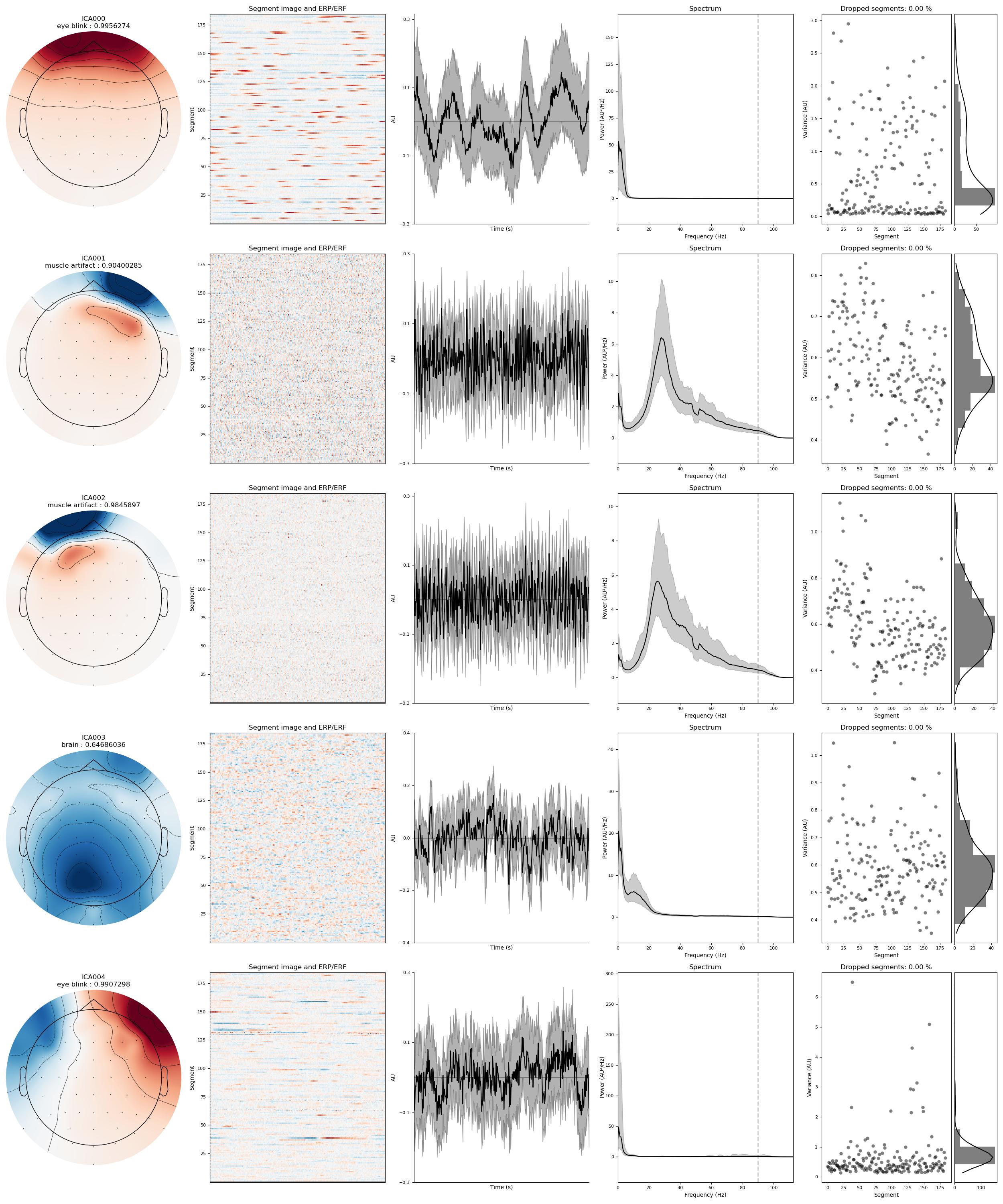

Supplement: Supplementary file 2 [file Data_Sheet_2.zip › component_image/sub07_session2_d1_block1112_0.jpg]

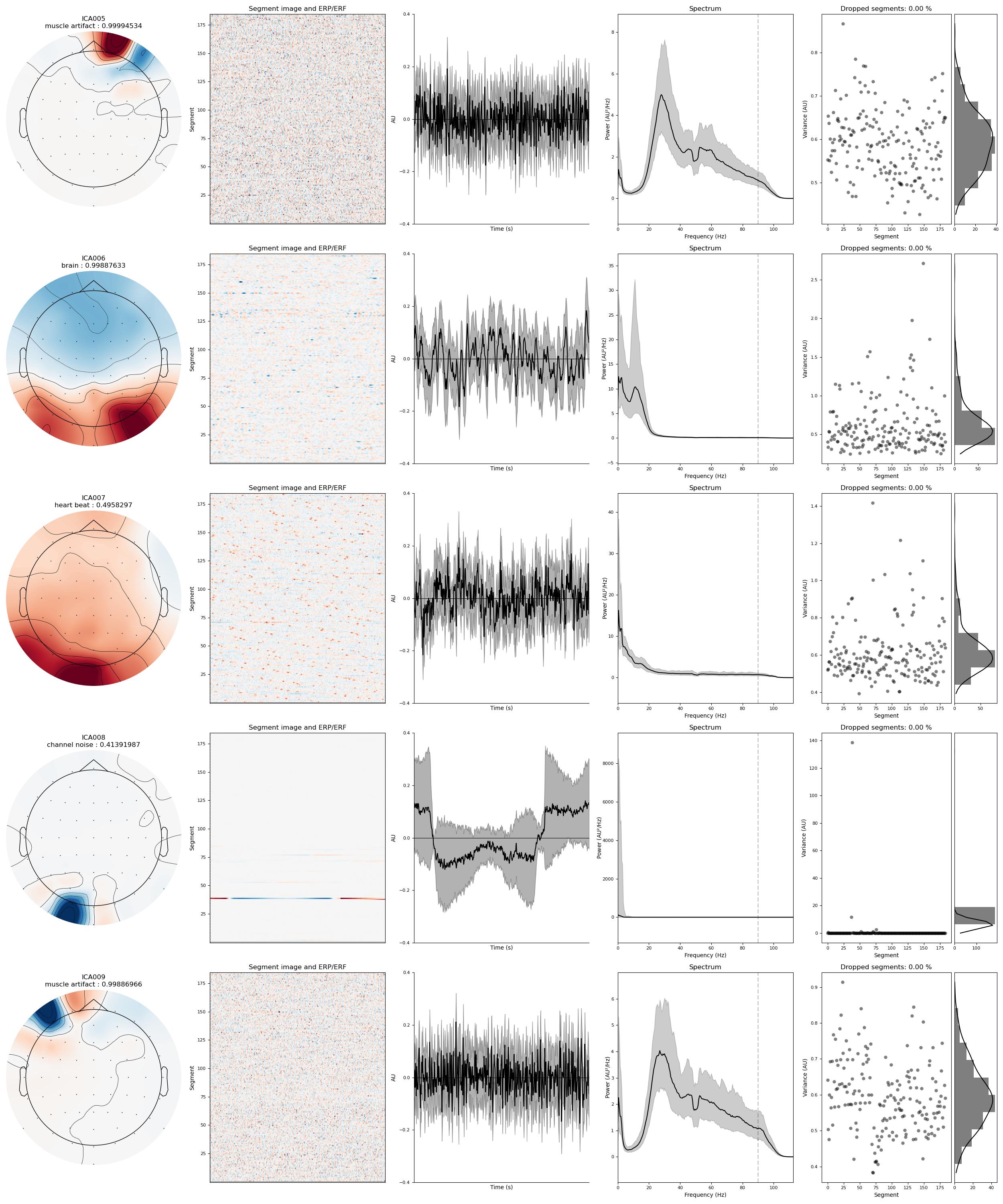

Supplement: Supplementary file 2 [file Data_Sheet_2.zip › component_image/sub07_session2_d1_block1112_1.jpg]

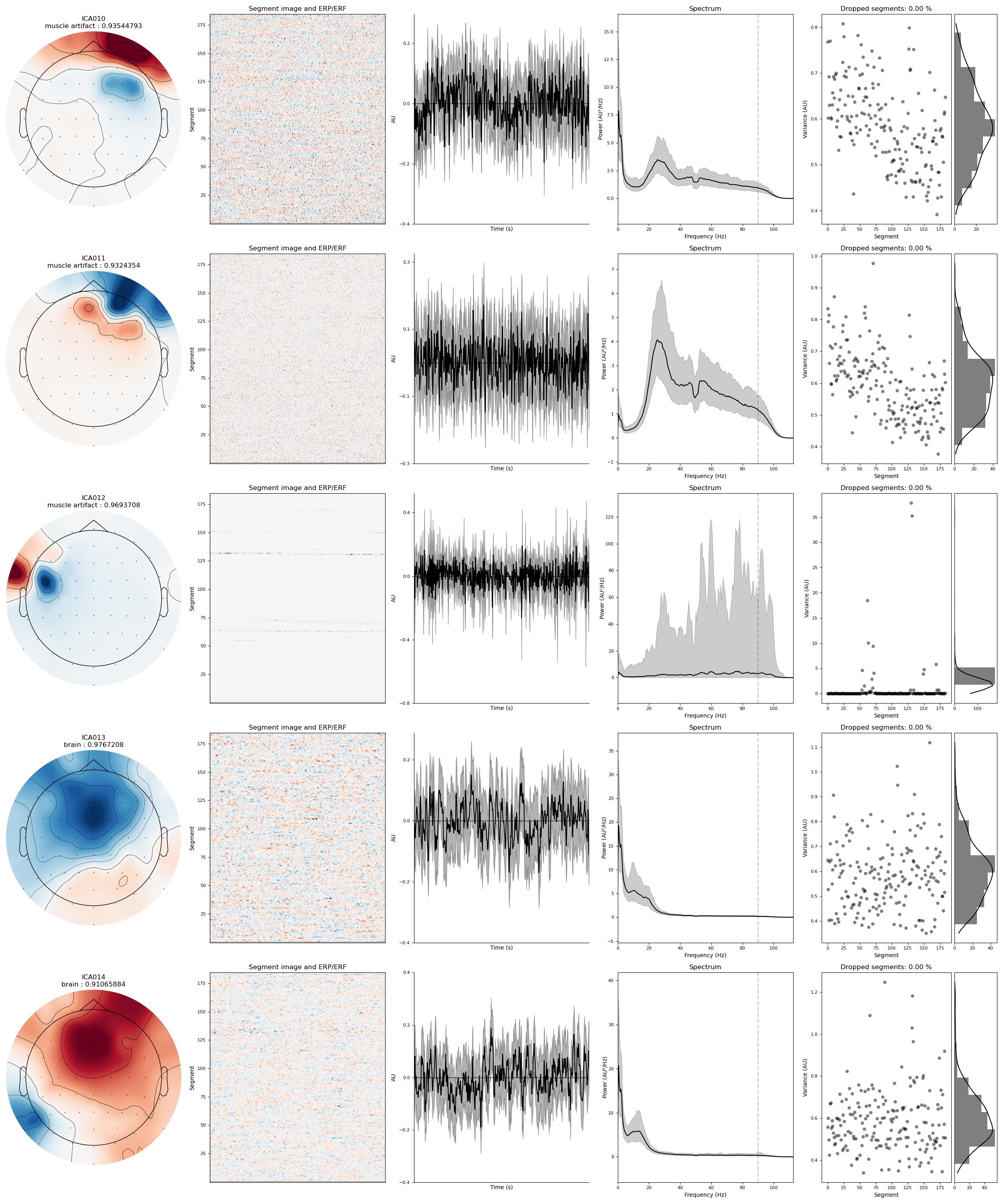

Supplement: Supplementary file 2 [file Data_Sheet_2.zip › component_image/sub07_session2_d1_block1112_2.jpg]

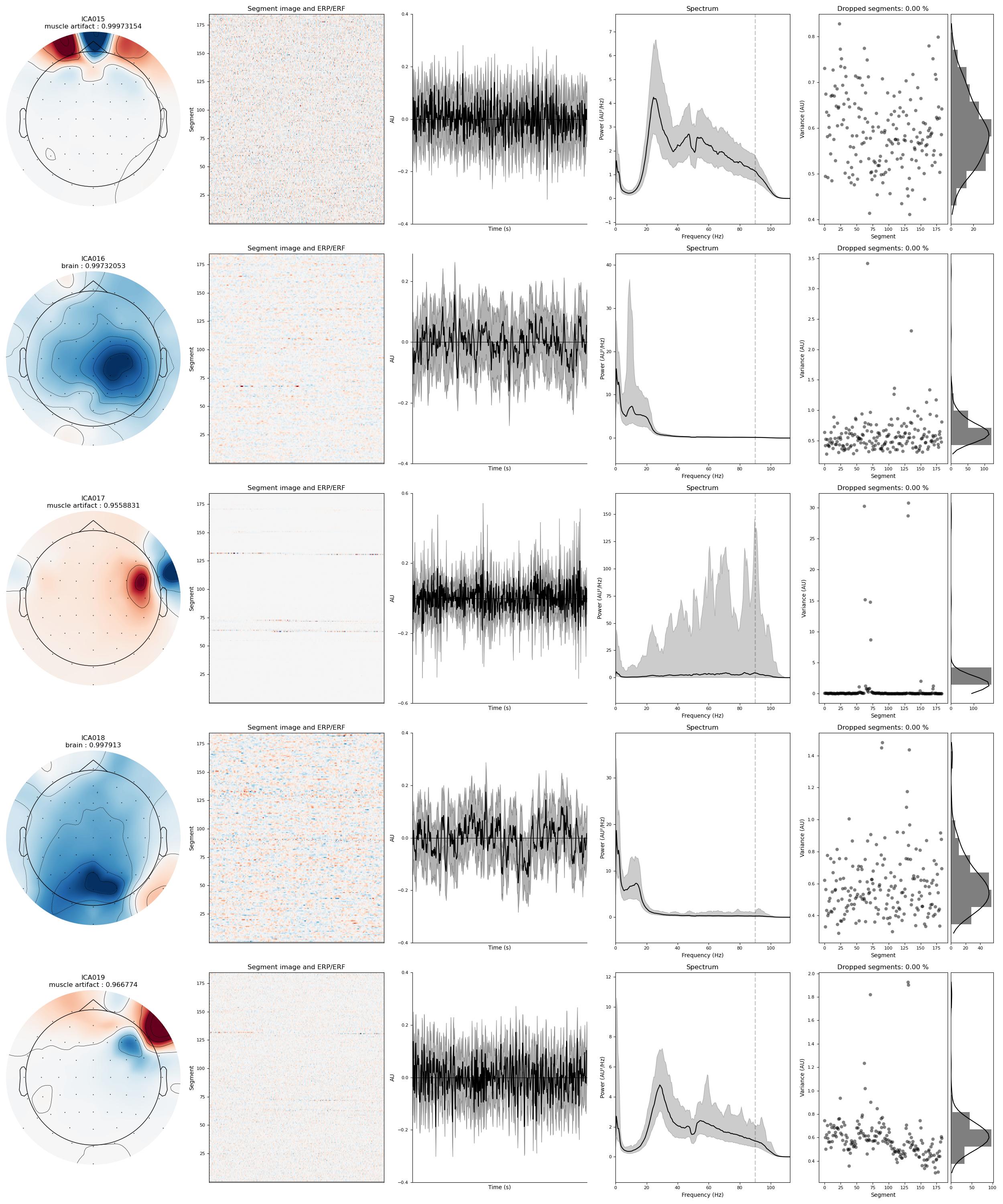

Supplement: Supplementary file 2 [file Data_Sheet_2.zip › component_image/sub07_session2_d1_block1112_3.jpg]

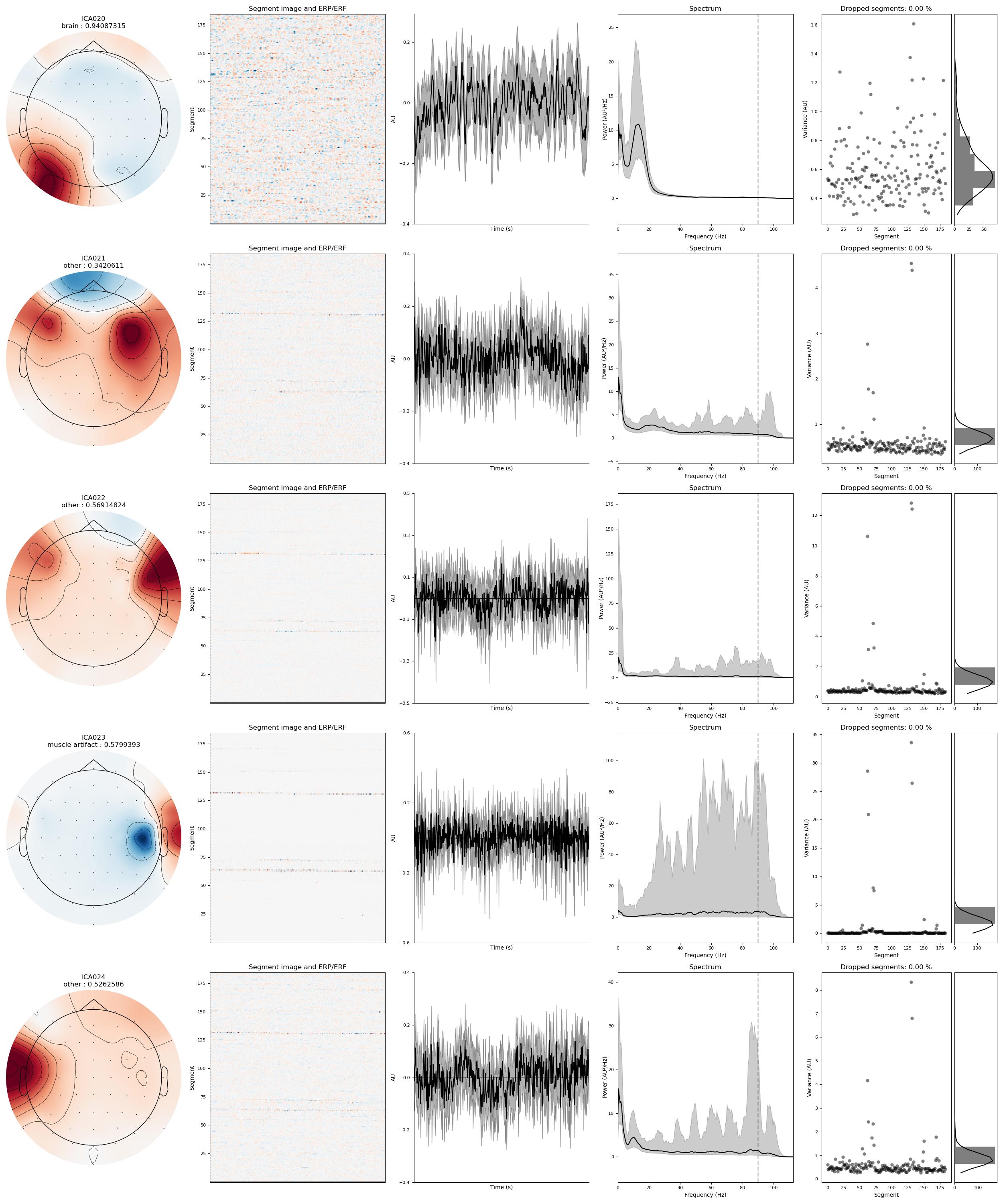

Supplement: Supplementary file 2 [file Data_Sheet_2.zip › component_image/sub07_session2_d1_block1112_4.jpg]

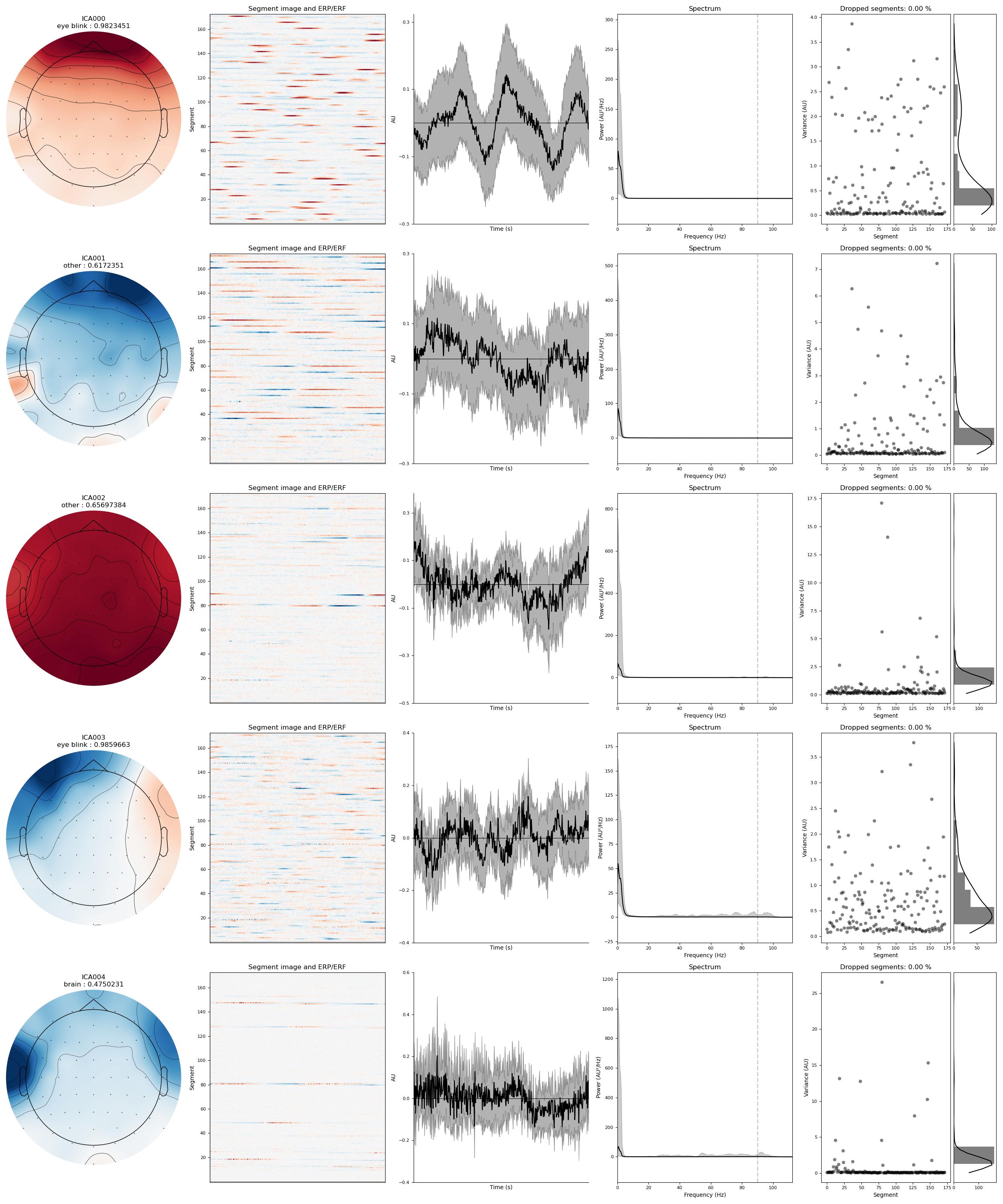

Supplement: Supplementary file 2 [file Data_Sheet_2.zip › component_image/sub09_session2_d1_block1112_0.jpg]

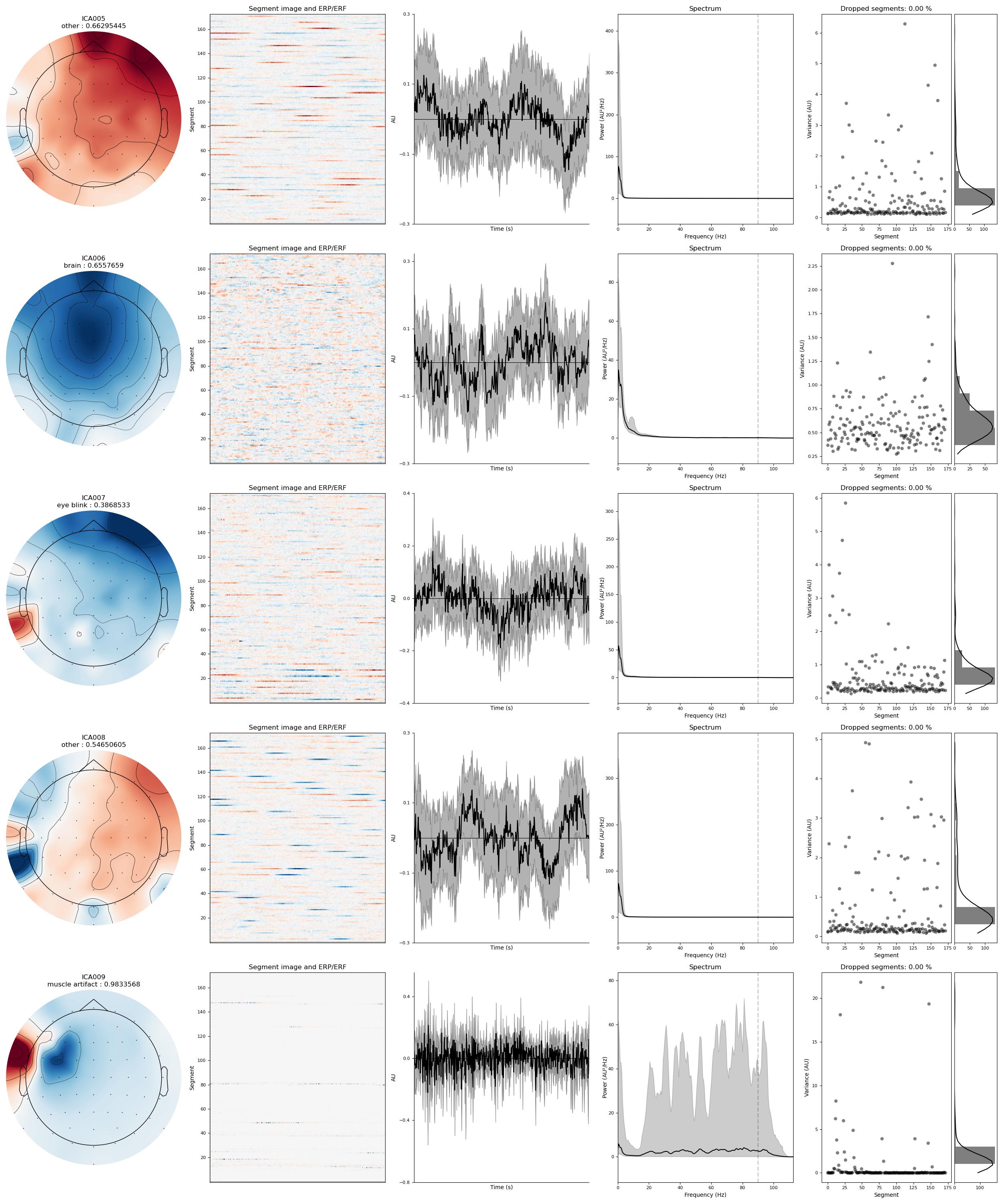

Supplement: Supplementary file 2 [file Data_Sheet_2.zip › component_image/sub09_session2_d1_block1112_1.jpg]

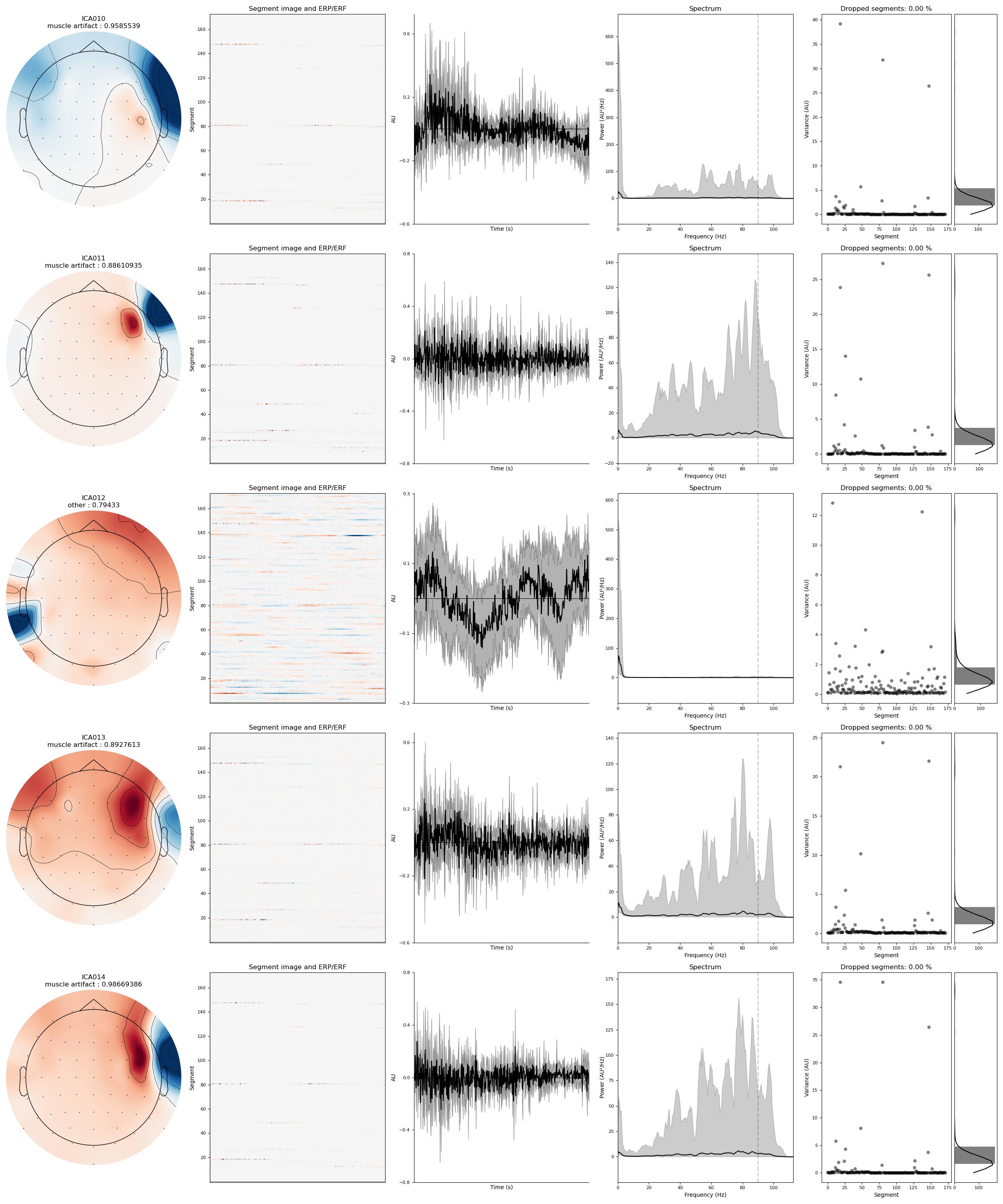

Supplement: Supplementary file 2 [file Data_Sheet_2.zip › component_image/sub09_session2_d1_block1112_2.jpg]

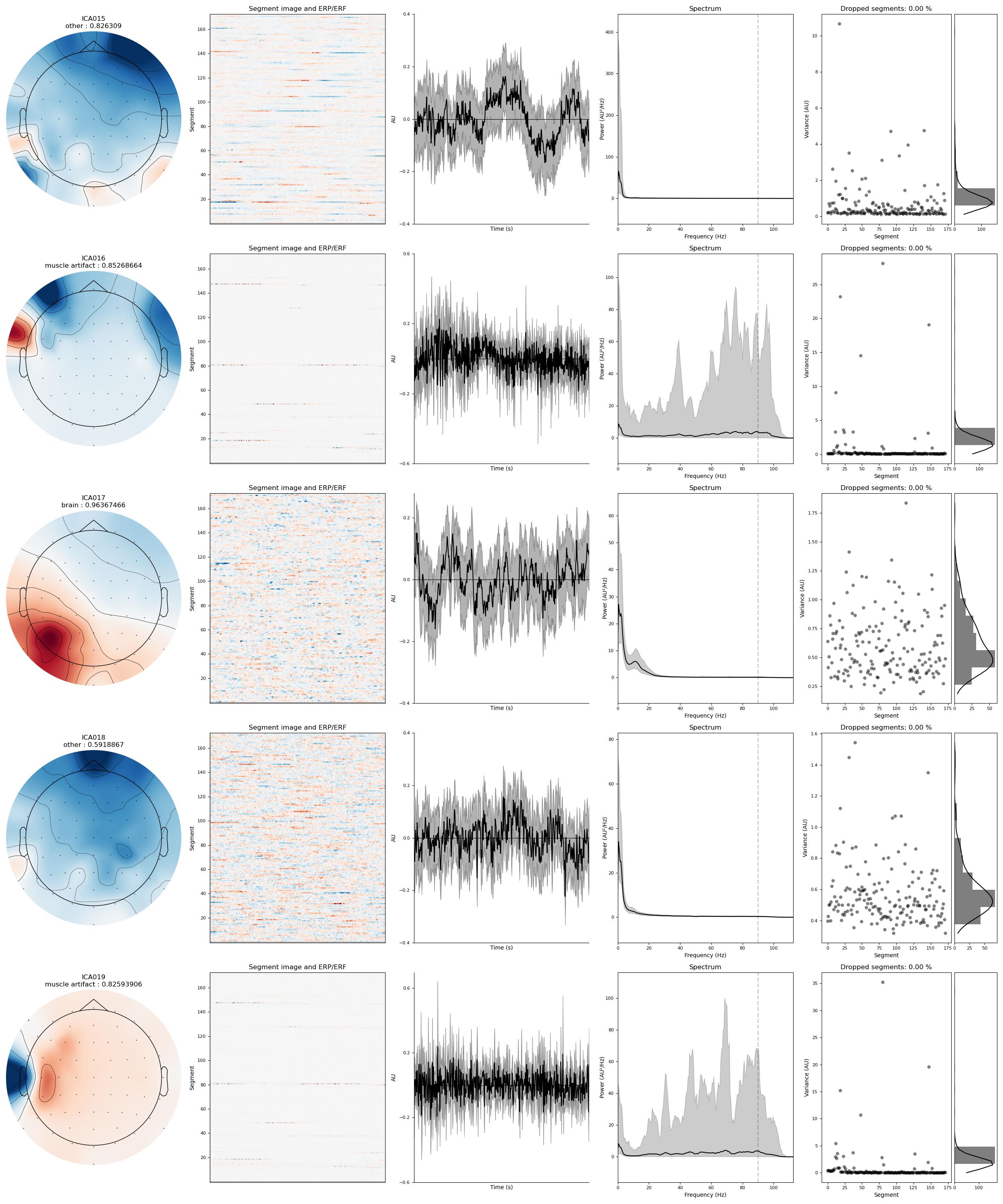

Supplement: Supplementary file 2 [file Data_Sheet_2.zip › component_image/sub09_session2_d1_block1112_3.jpg]

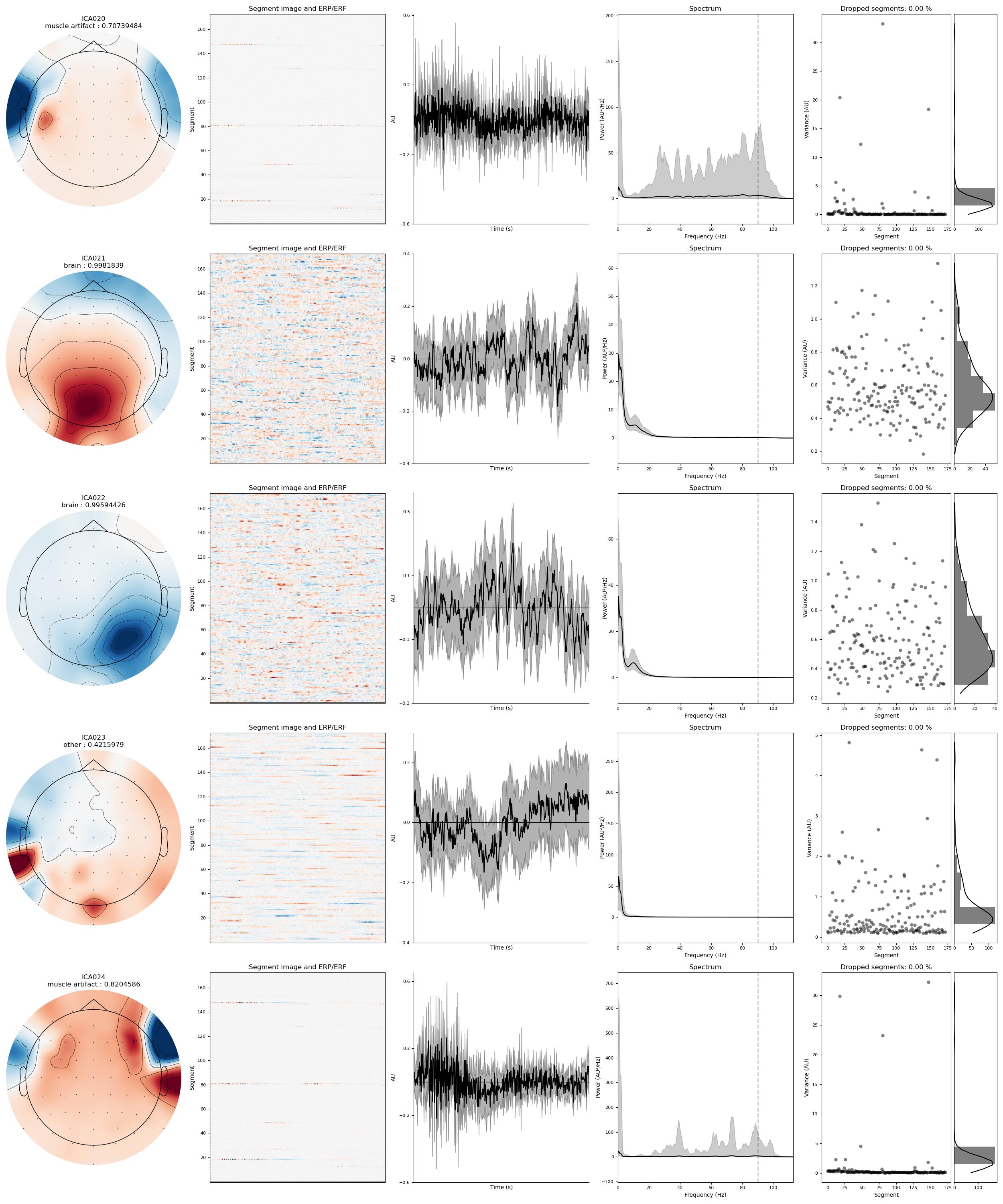

Supplement: Supplementary file 2 [file Data_Sheet_2.zip › component_image/sub09_session2_d1_block1112_4.jpg]

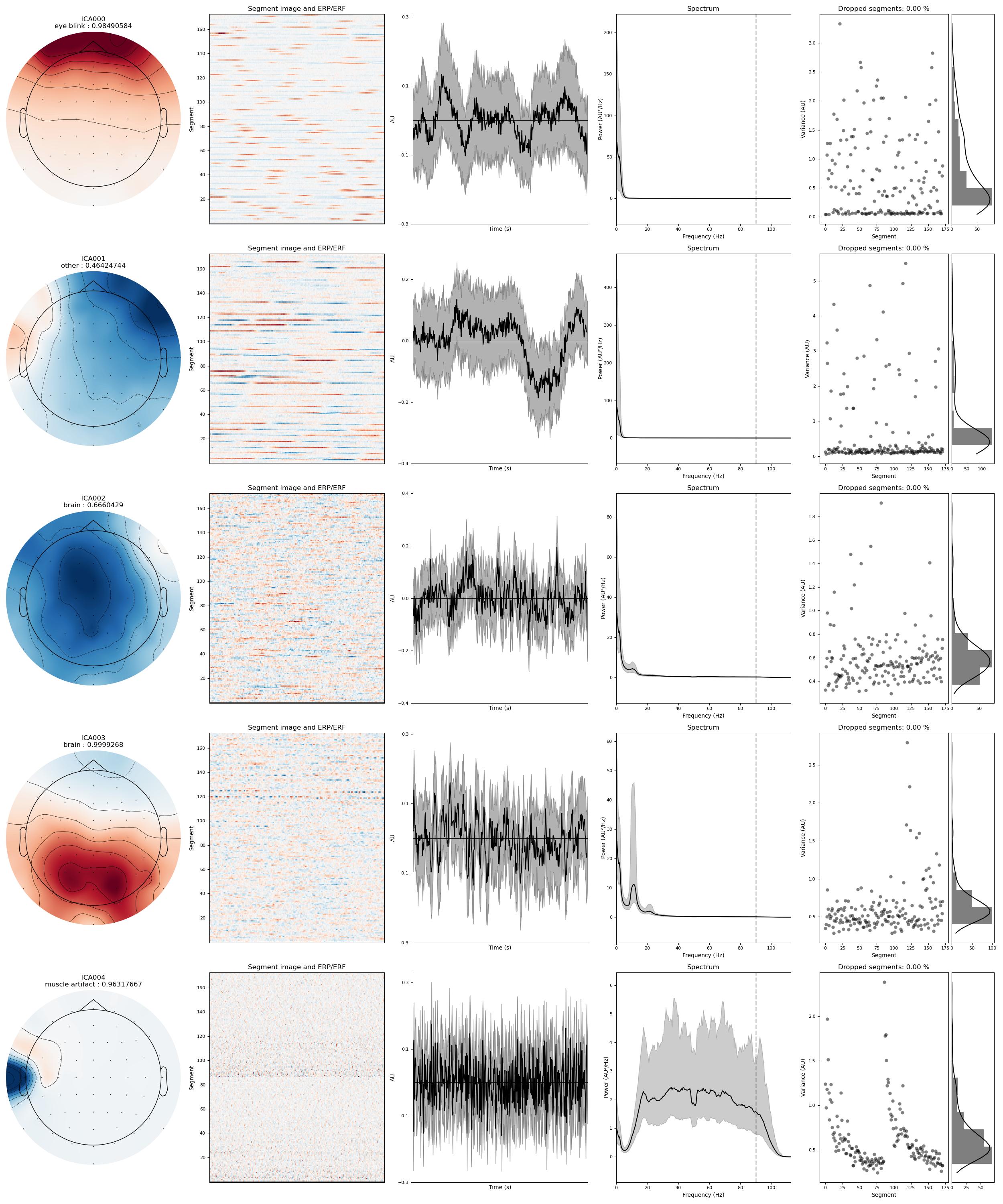

Supplement: Supplementary file 2 [file Data_Sheet_2.zip › component_image/sub10_session2_d1_block1112_0.jpg]

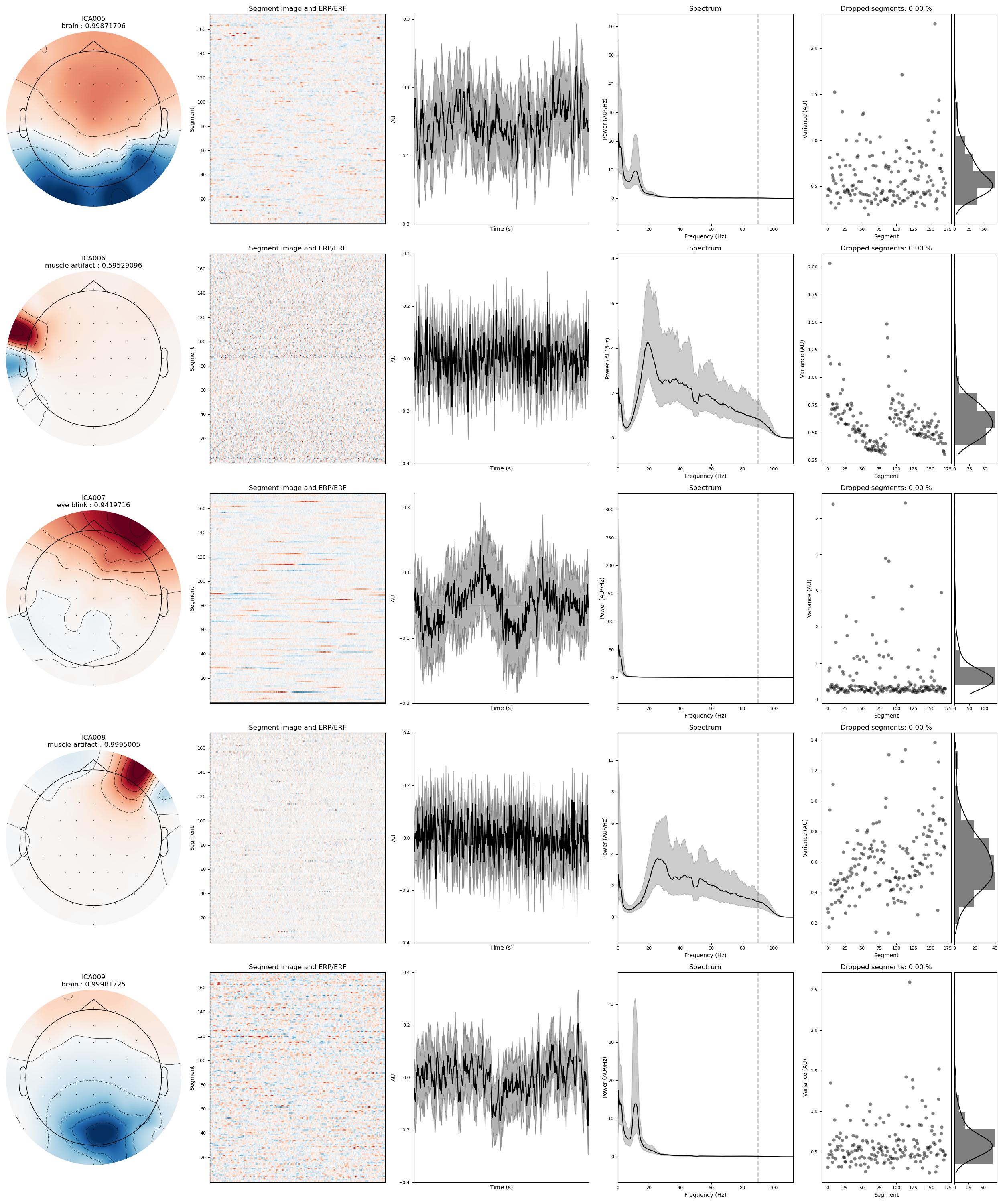

Supplement: Supplementary file 2 [file Data_Sheet_2.zip › component_image/sub10_session2_d1_block1112_1.jpg]

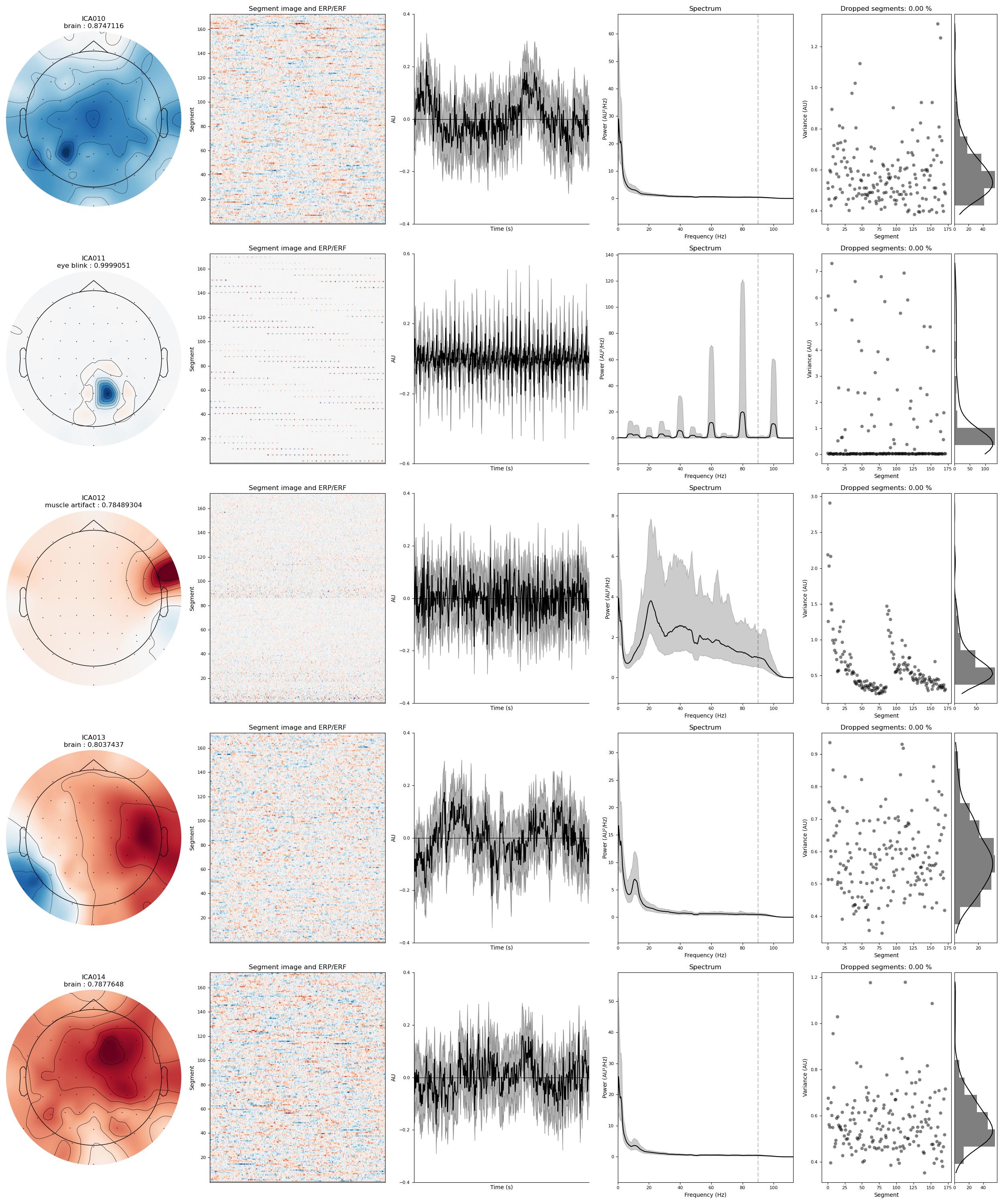

Supplement: Supplementary file 2 [file Data_Sheet_2.zip › component_image/sub10_session2_d1_block1112_2.jpg]

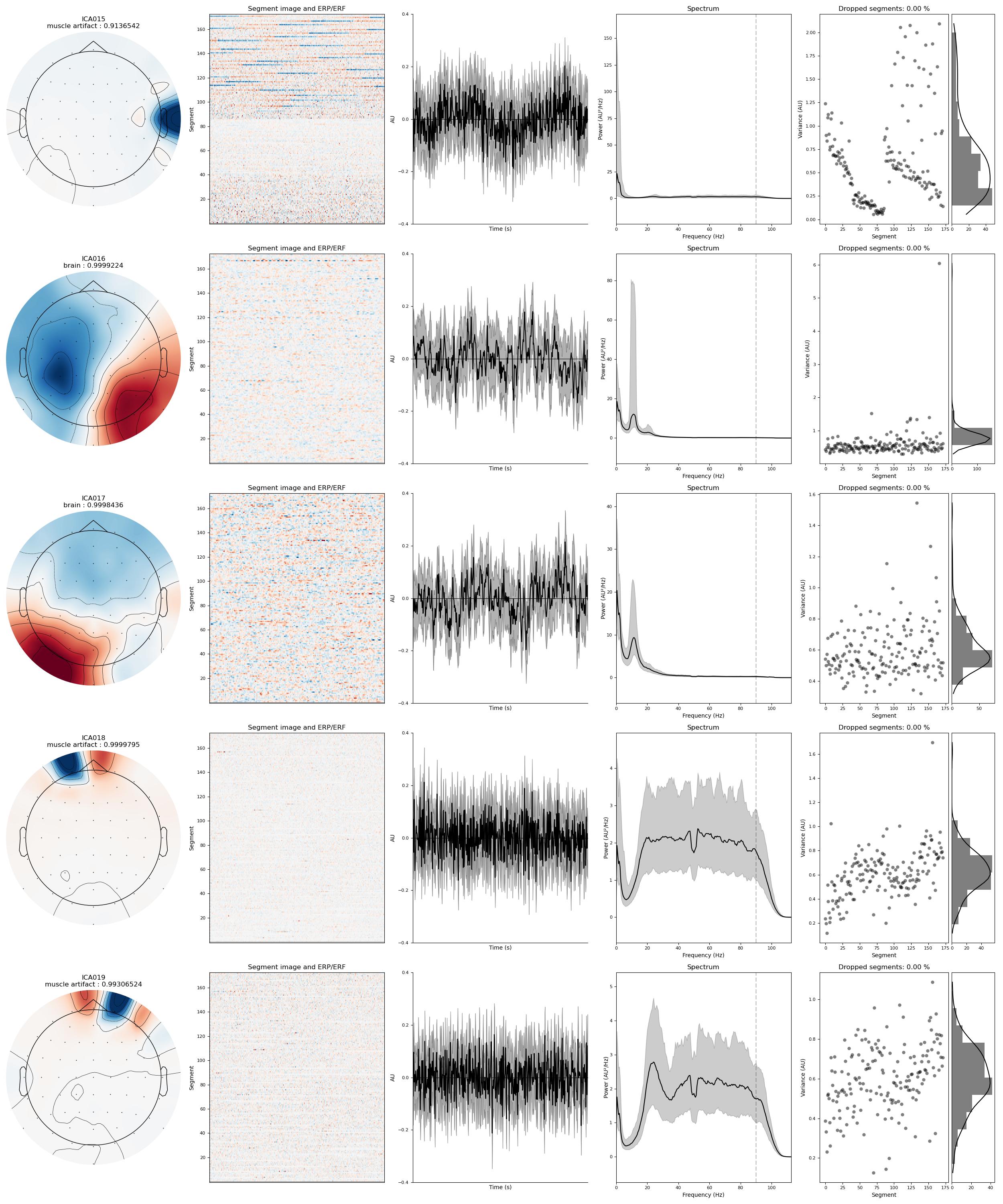

Supplement: Supplementary file 2 [file Data_Sheet_2.zip › component_image/sub10_session2_d1_block1112_3.jpg]

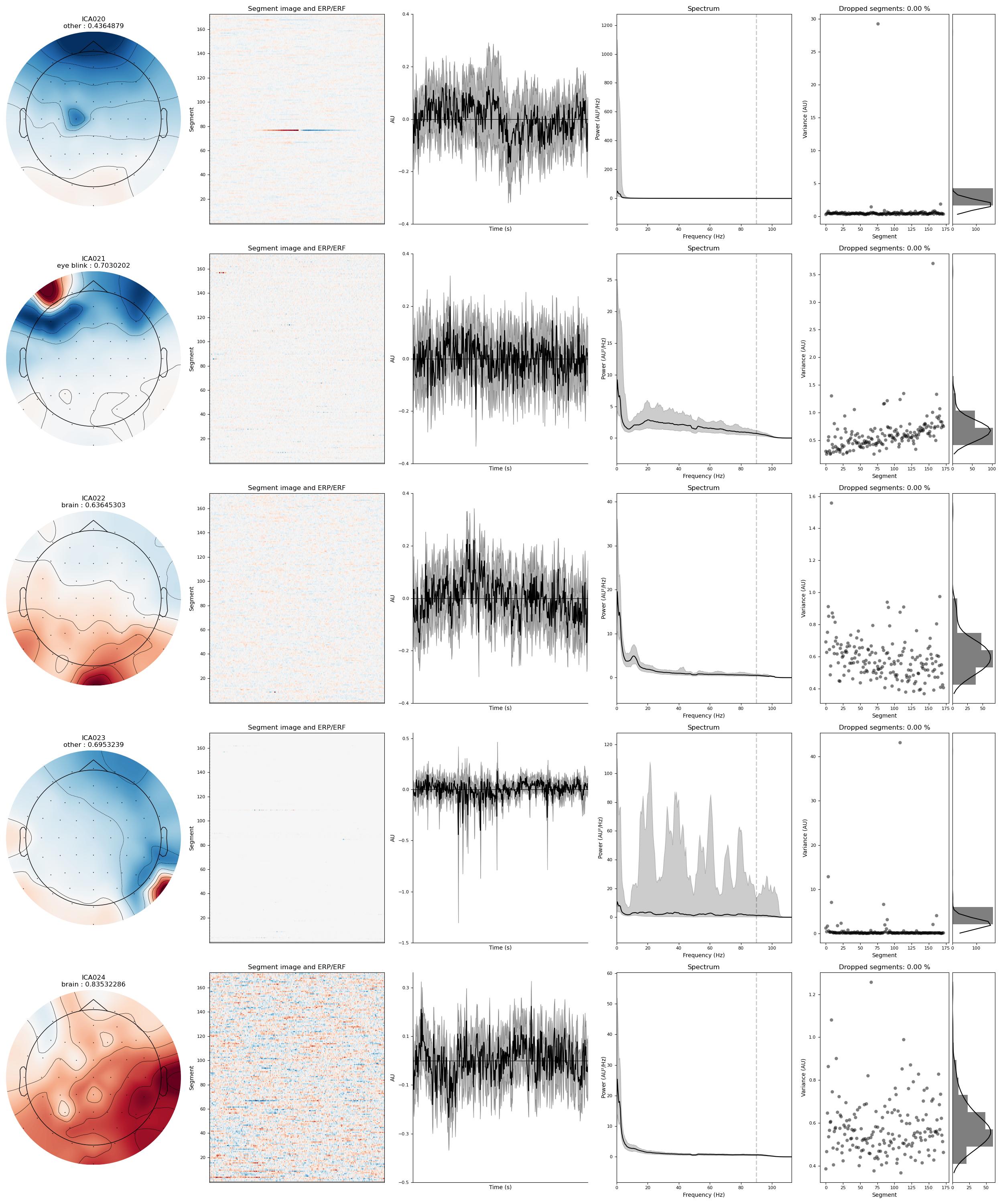

Supplement: Supplementary file 2 [file Data_Sheet_2.zip › component_image/sub10_session2_d1_block1112_4.jpg]

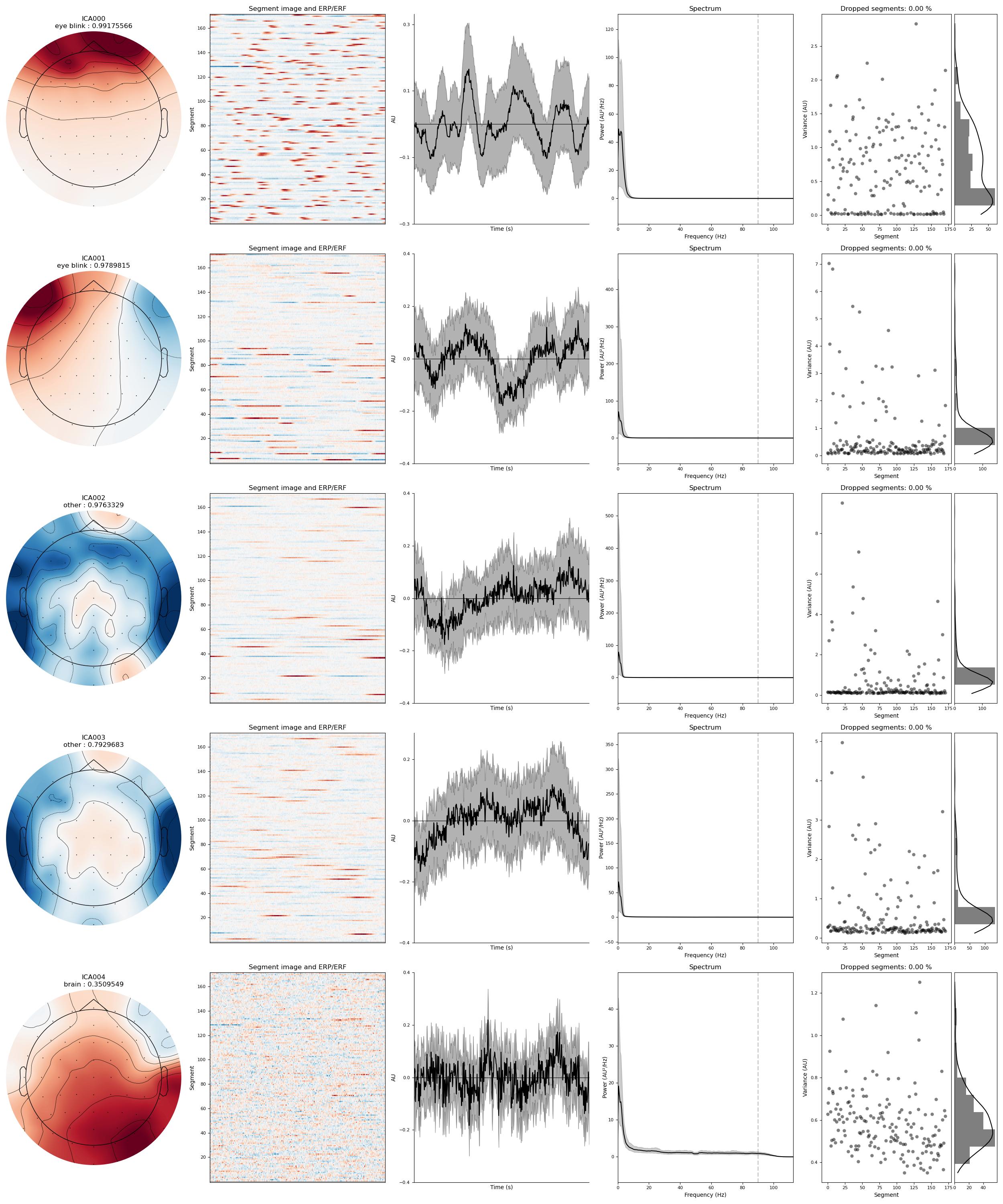

Supplement: Supplementary file 2 [file Data_Sheet_2.zip › component_image/sub12_session2_d1_block1112_0.jpg]

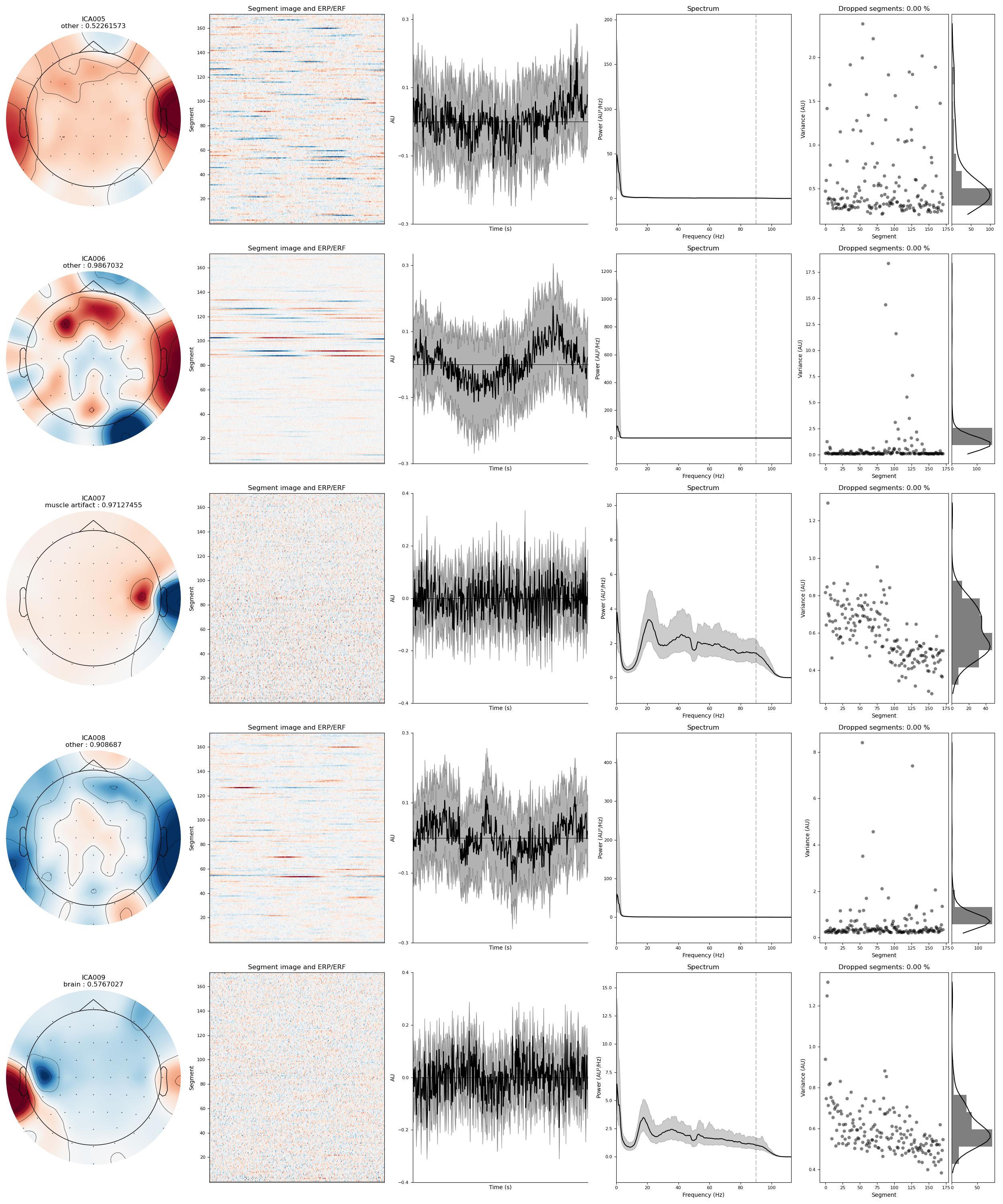

Supplement: Supplementary file 2 [file Data_Sheet_2.zip › component_image/sub12_session2_d1_block1112_1.jpg]

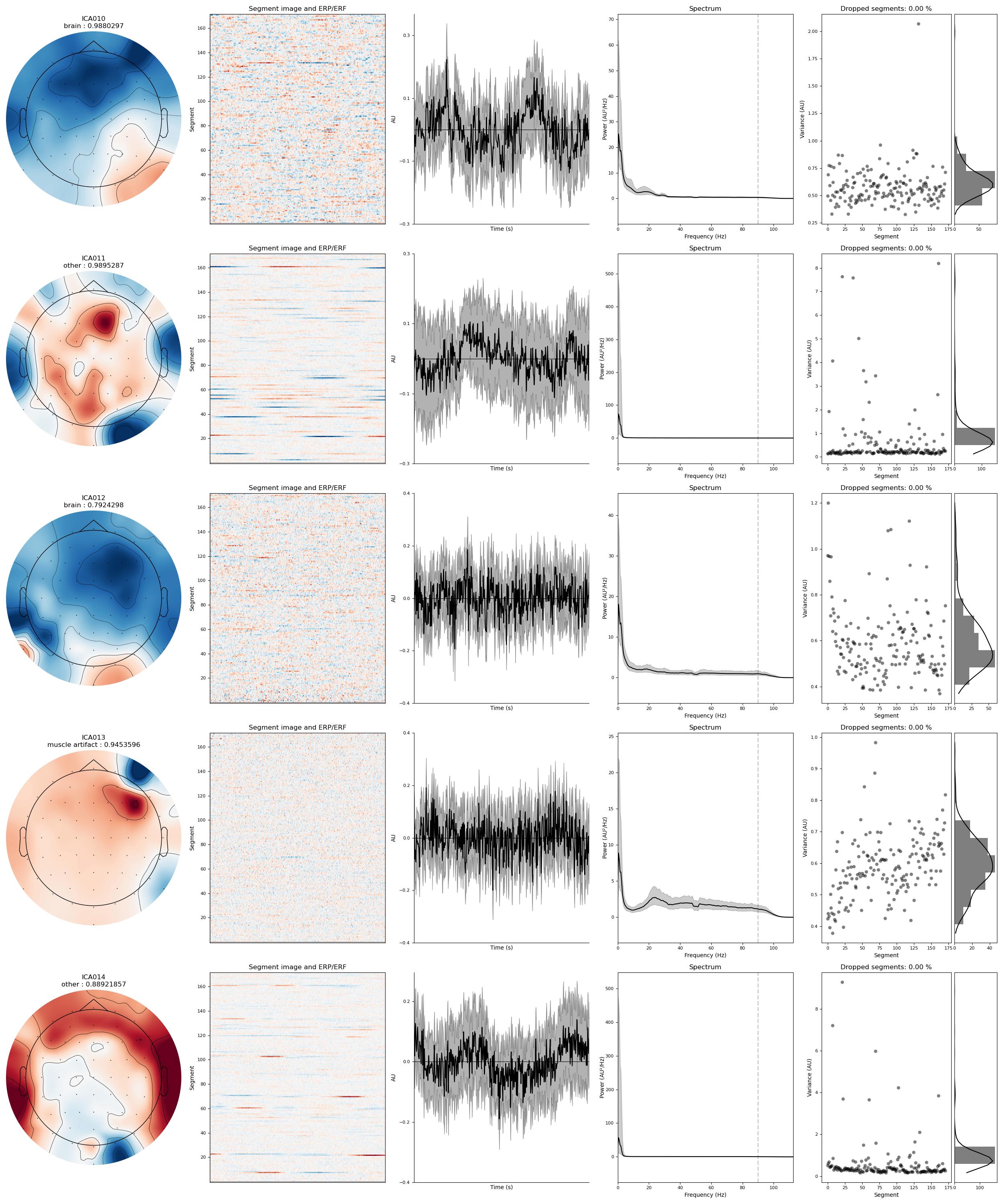

Supplement: Supplementary file 2 [file Data_Sheet_2.zip › component_image/sub12_session2_d1_block1112_2.jpg]

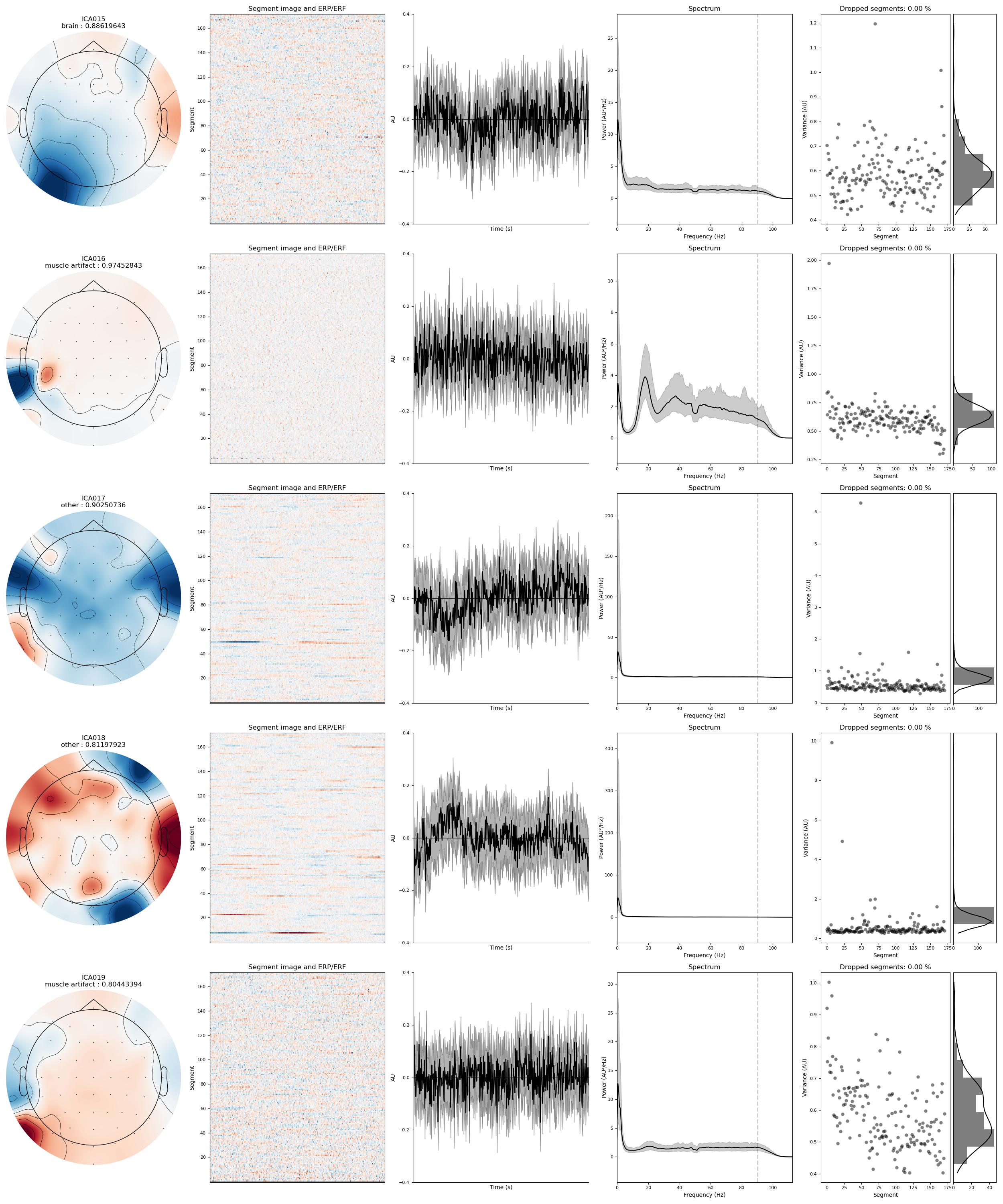

Supplement: Supplementary file 2 [file Data_Sheet_2.zip › component_image/sub12_session2_d1_block1112_3.jpg]

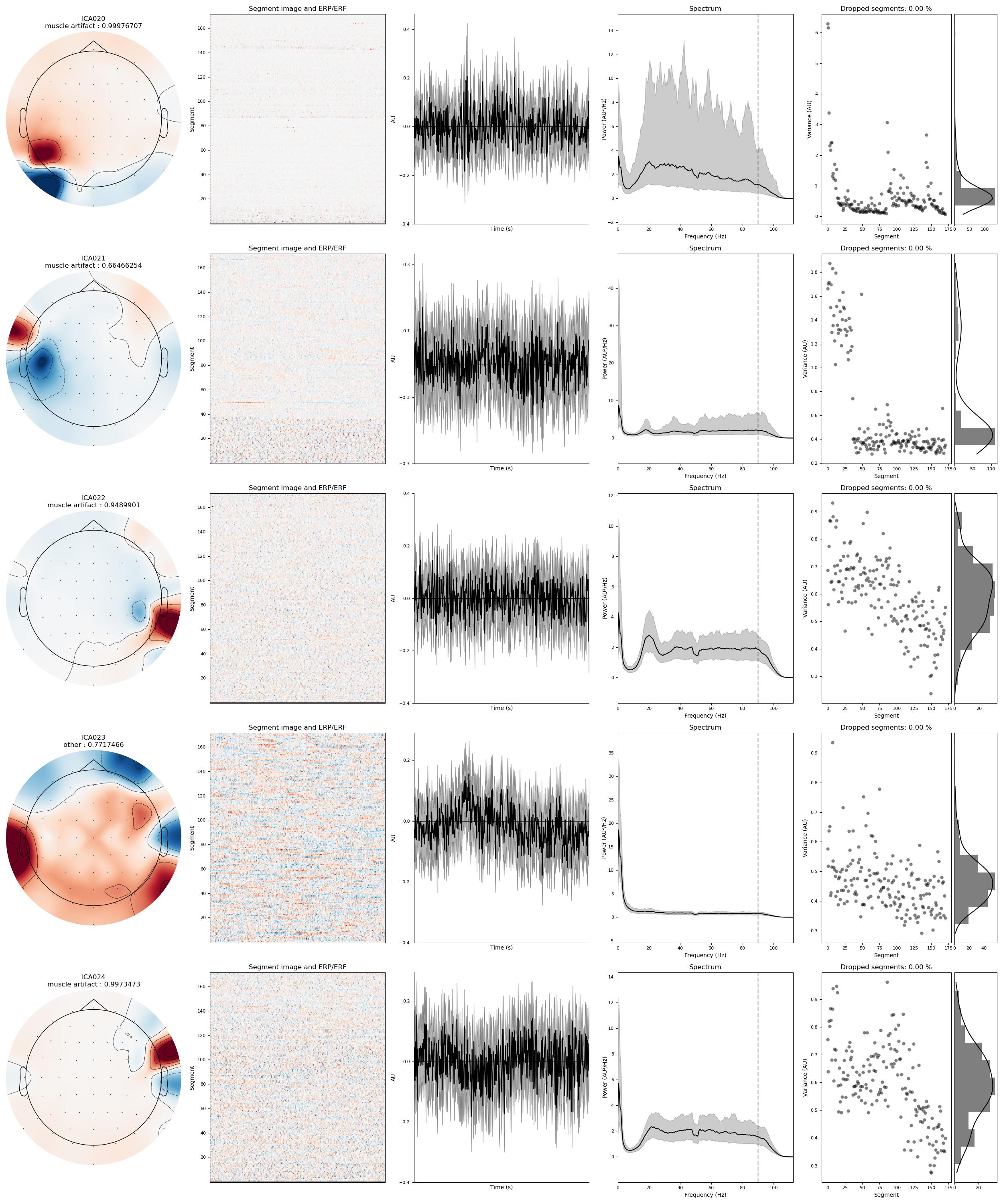

Supplement: Supplementary file 2 [file Data_Sheet_2.zip › component_image/sub12_session2_d1_block1112_4.jpg]

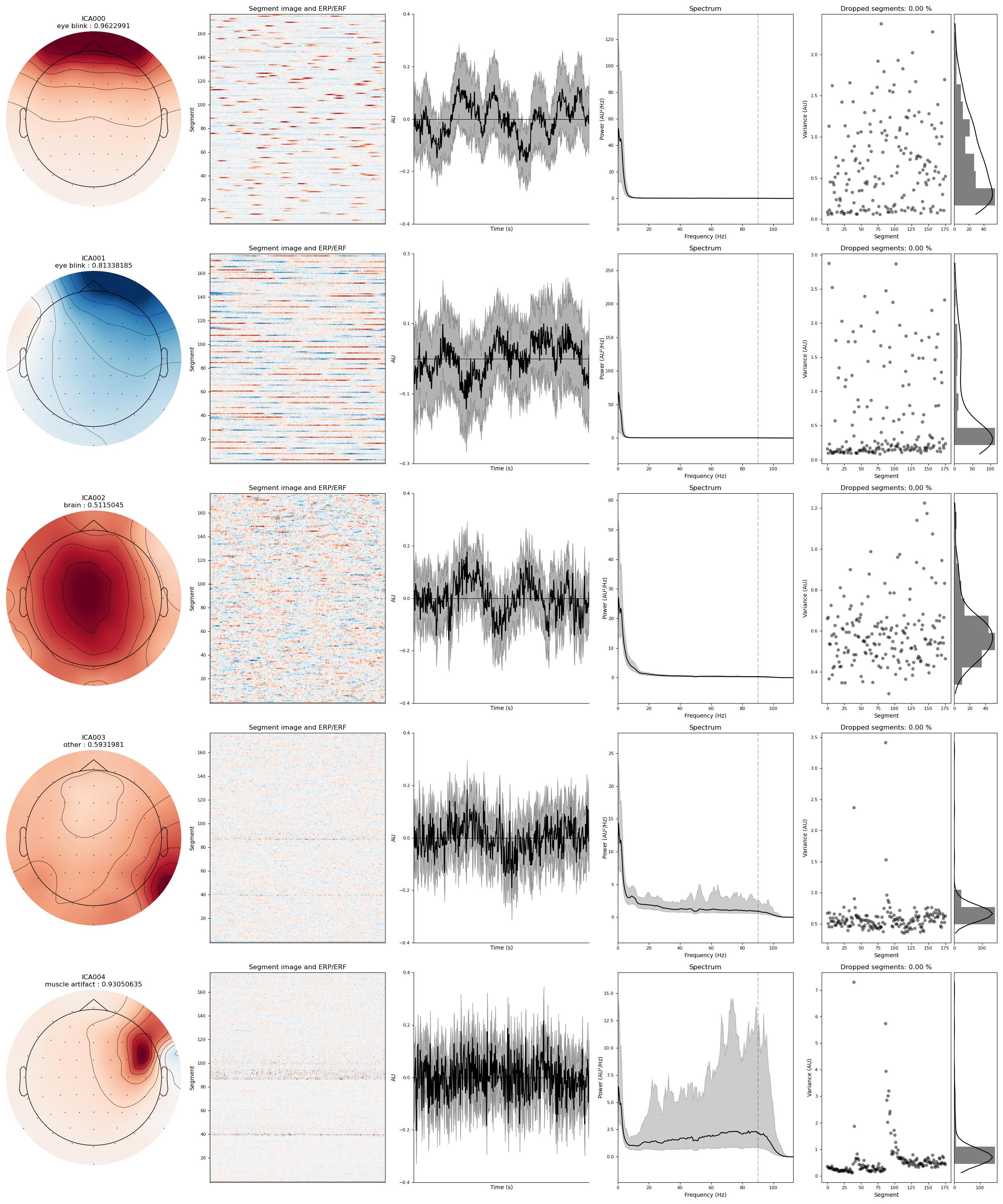

Supplement: Supplementary file 2 [file Data_Sheet_2.zip › component_image/sub14_session2_d1_block1112_0.jpg]

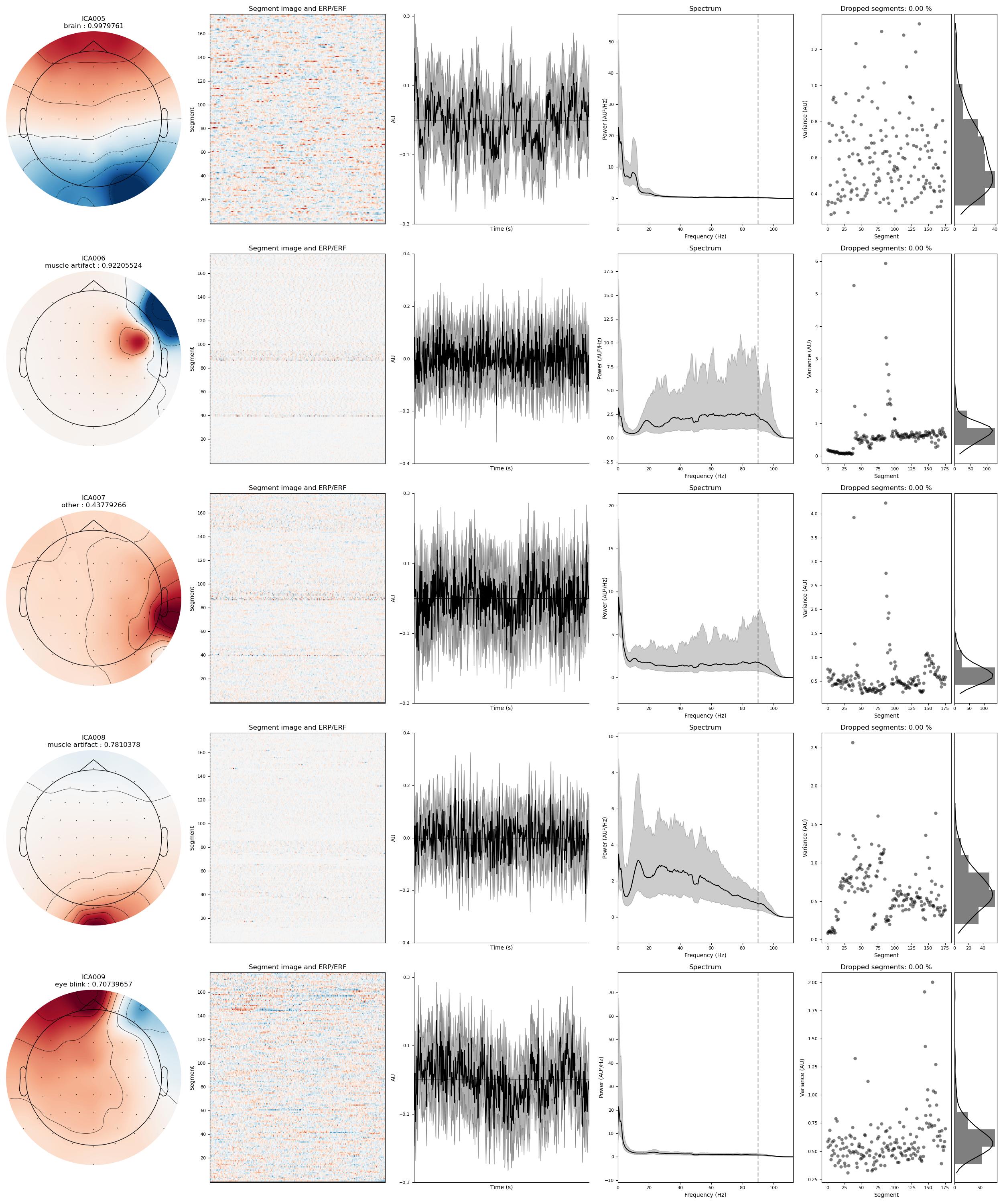

Supplement: Supplementary file 2 [file Data_Sheet_2.zip › component_image/sub14_session2_d1_block1112_1.jpg]

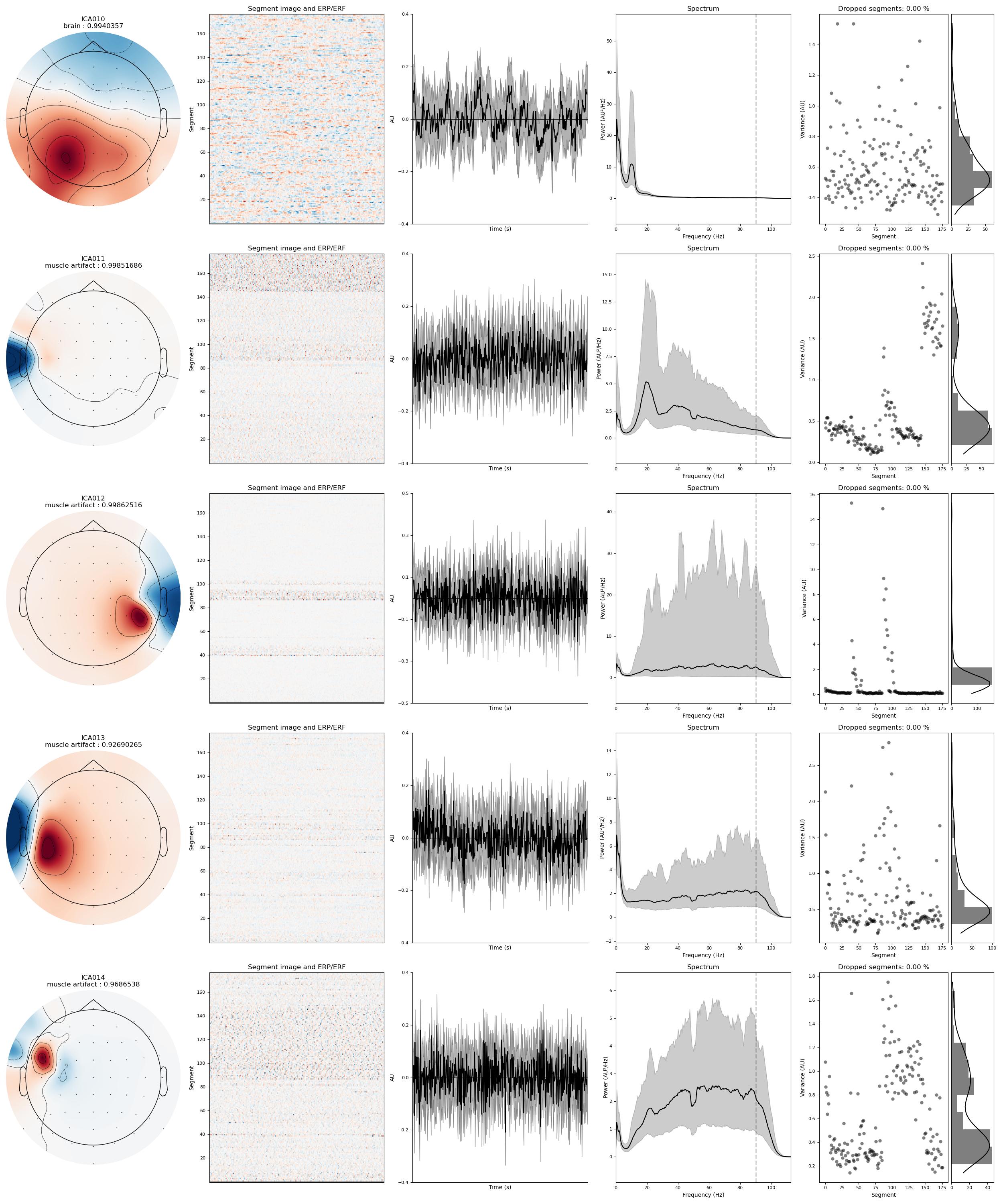

Supplement: Supplementary file 2 [file Data_Sheet_2.zip › component_image/sub14_session2_d1_block1112_2.jpg]

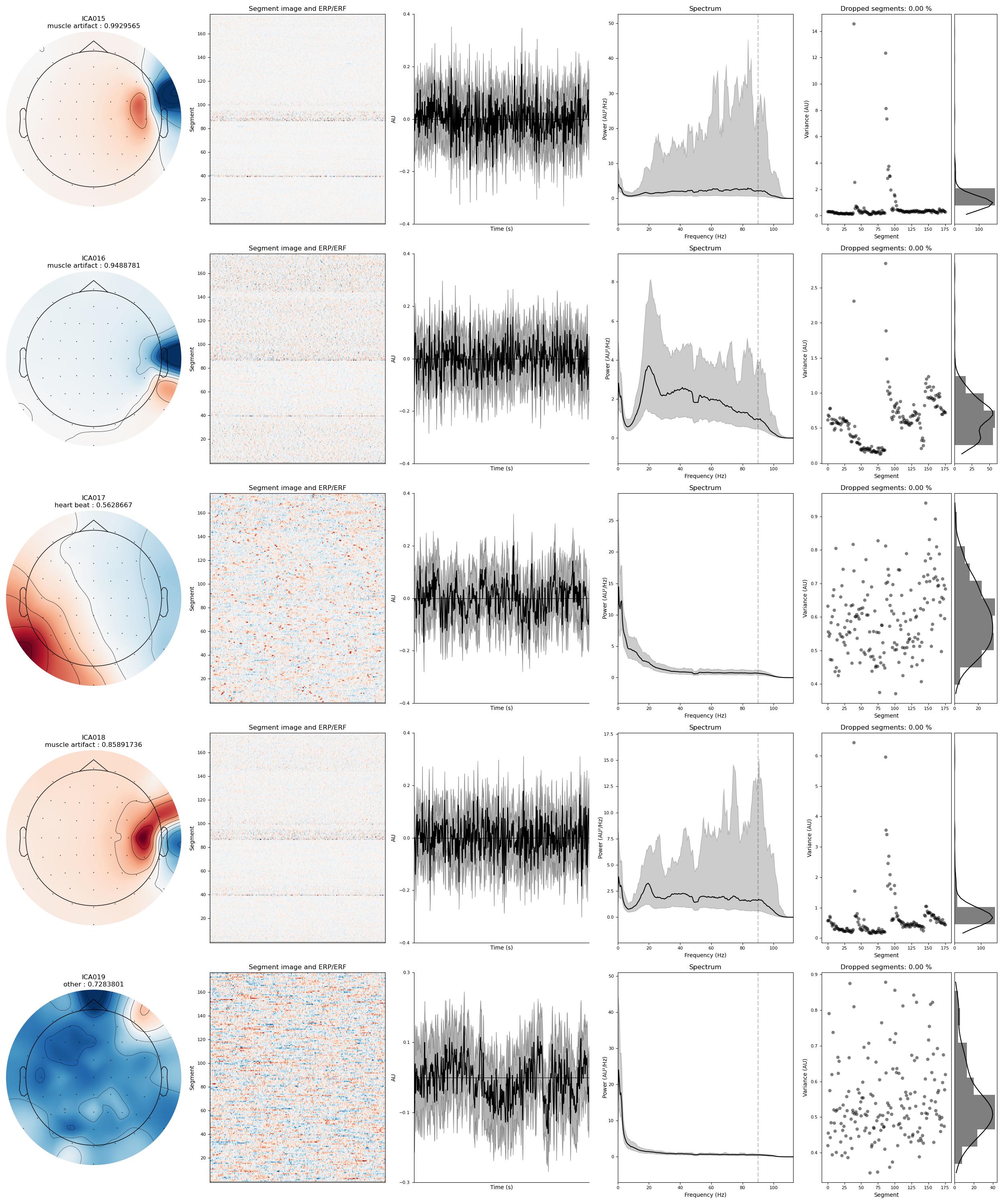

Supplement: Supplementary file 2 [file Data_Sheet_2.zip › component_image/sub14_session2_d1_block1112_3.jpg]

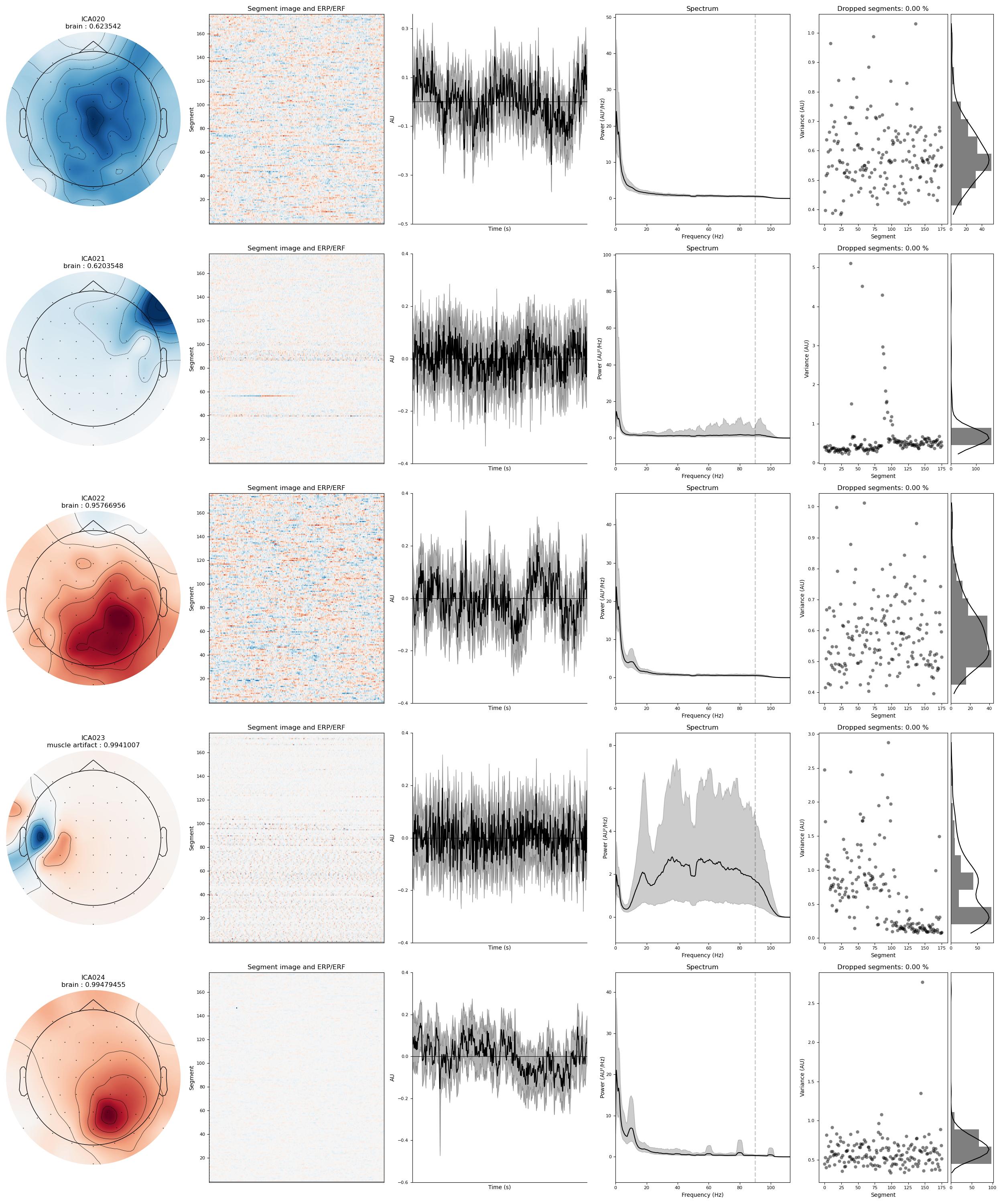

Supplement: Supplementary file 2 [file Data_Sheet_2.zip › component_image/sub14_session2_d1_block1112_4.jpg]

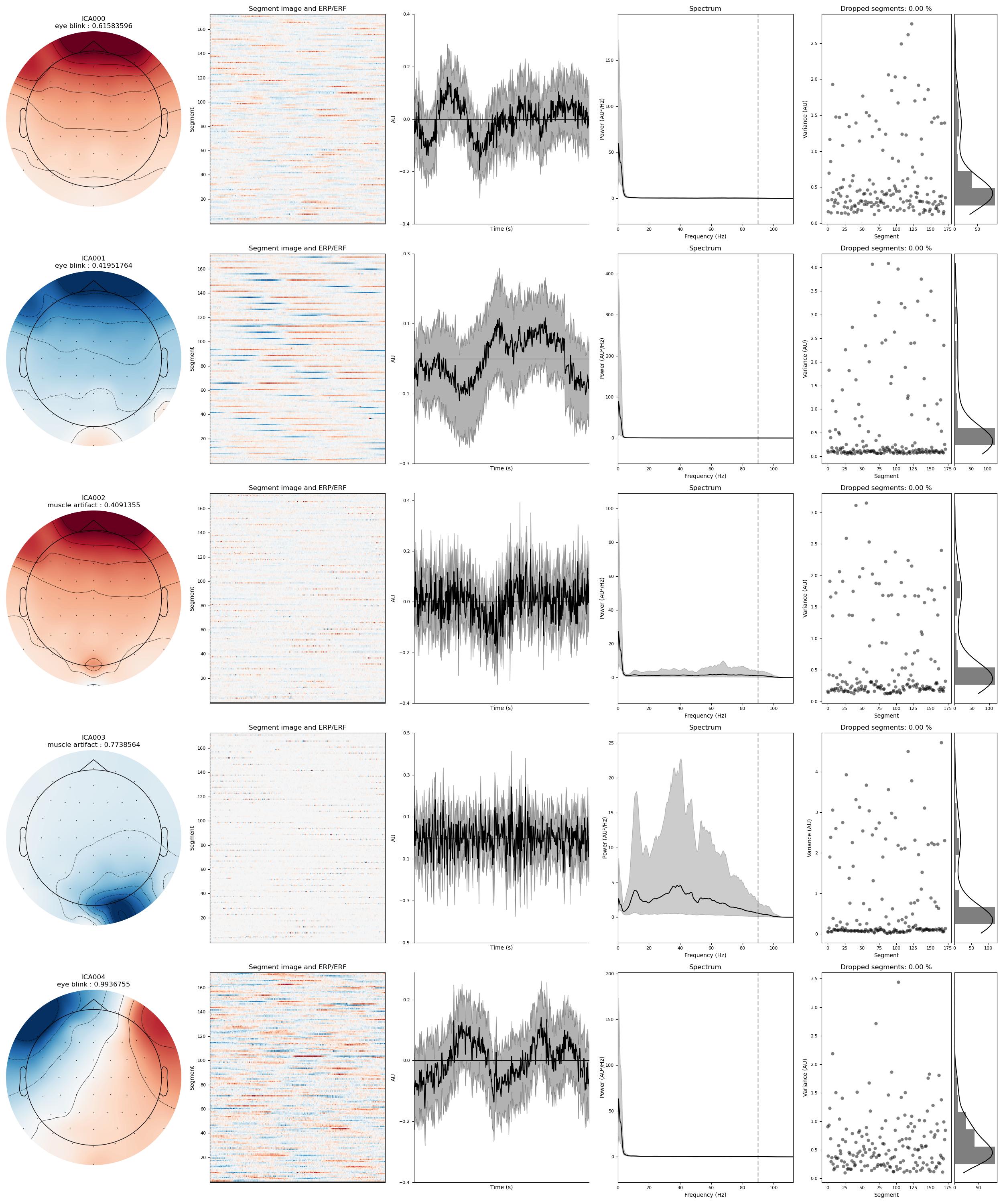

Supplement: Supplementary file 2 [file Data_Sheet_2.zip › component_image/sub16_session2_d1_block1112_0.jpg]

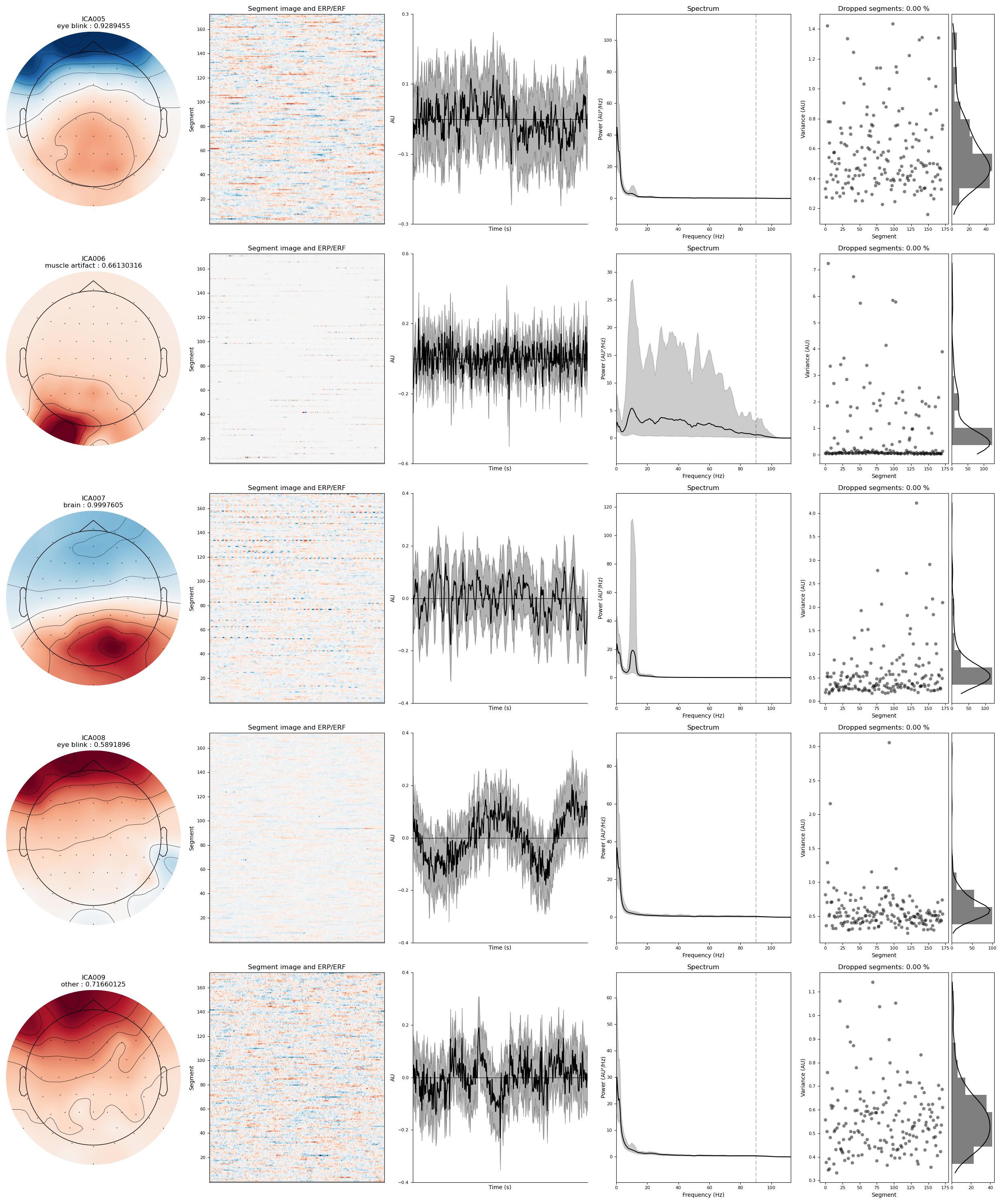

Supplement: Supplementary file 2 [file Data_Sheet_2.zip › component_image/sub16_session2_d1_block1112_1.jpg]

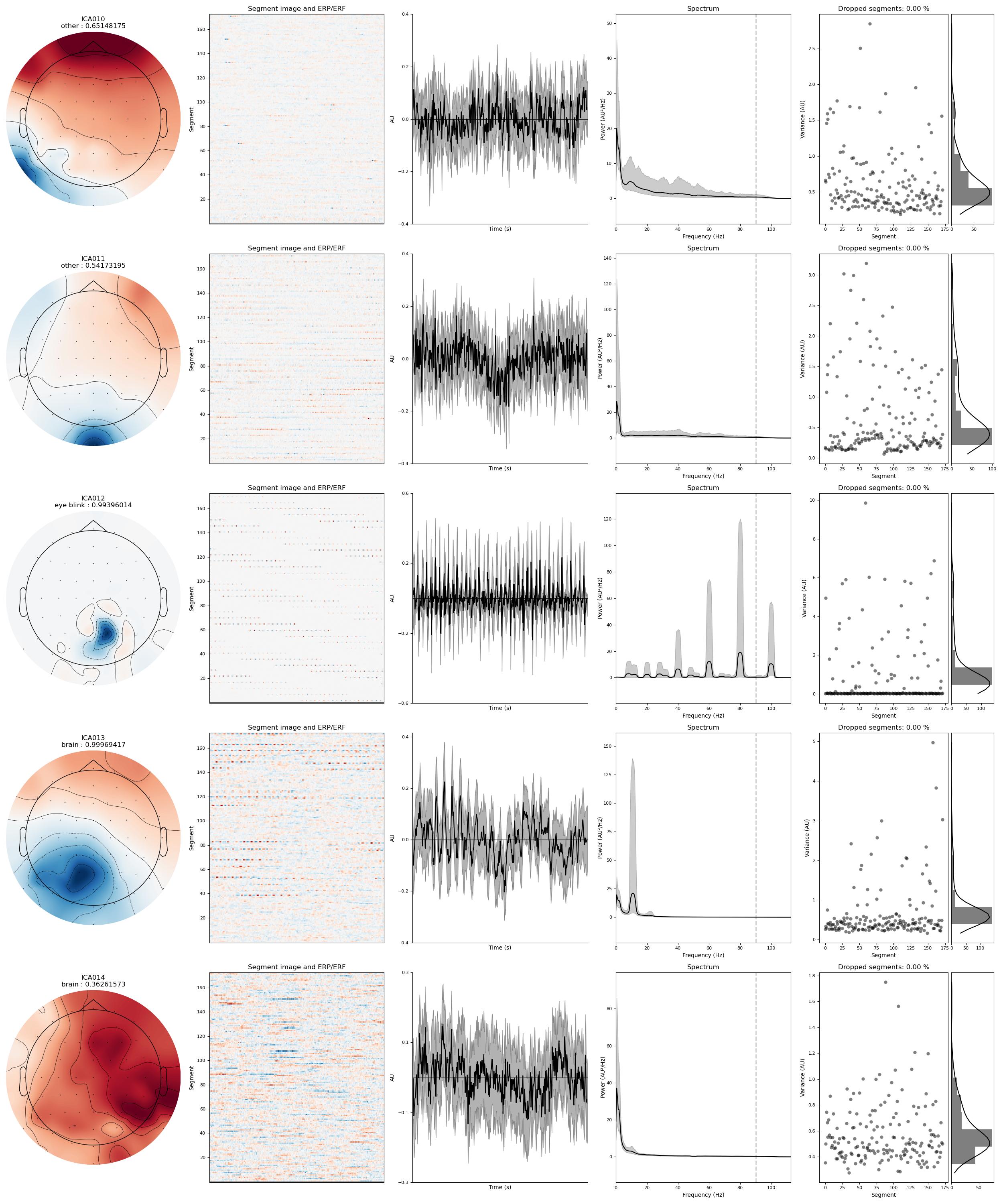

Supplement: Supplementary file 2 [file Data_Sheet_2.zip › component_image/sub16_session2_d1_block1112_2.jpg]

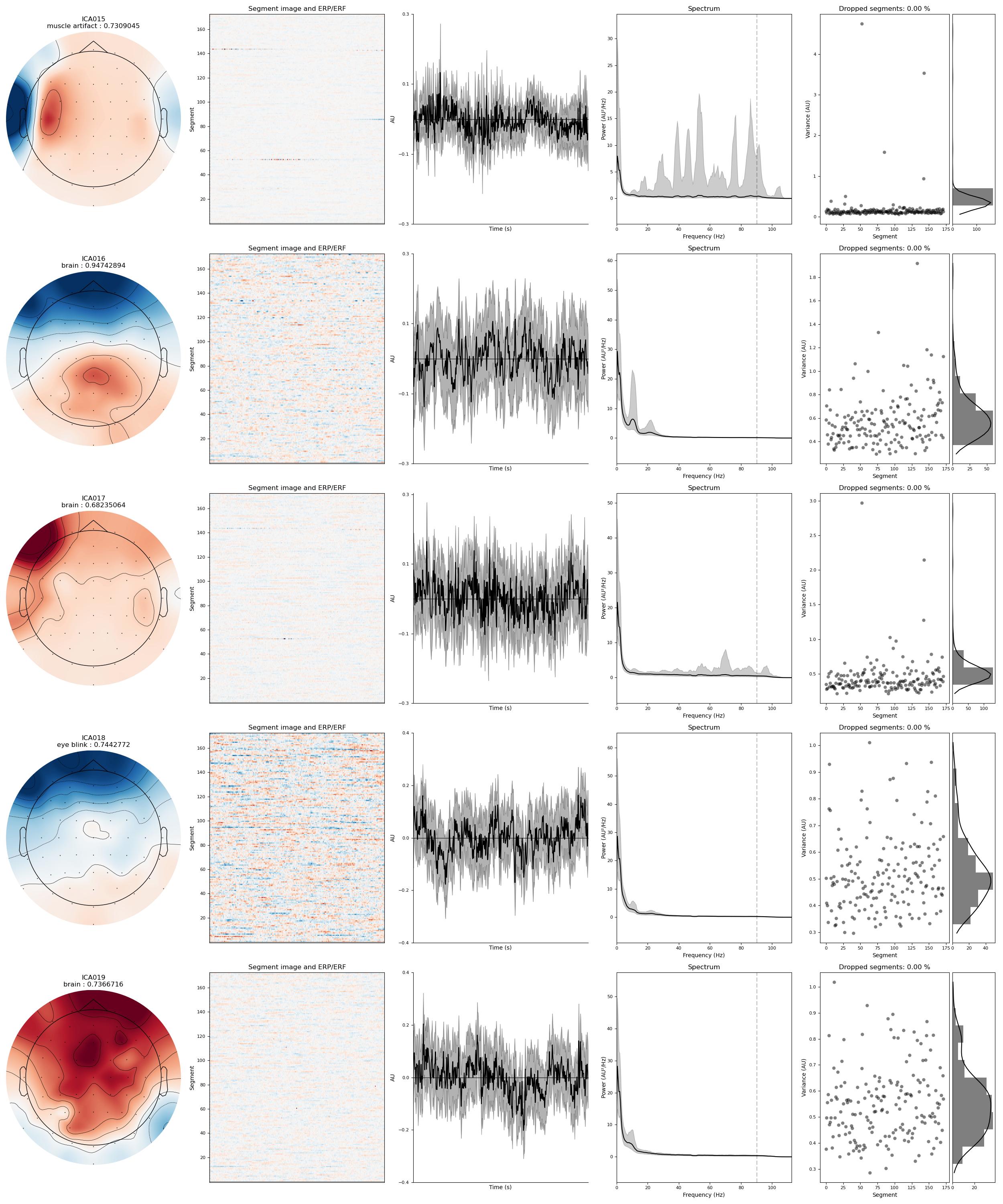

Supplement: Supplementary file 2 [file Data_Sheet_2.zip › component_image/sub16_session2_d1_block1112_3.jpg]

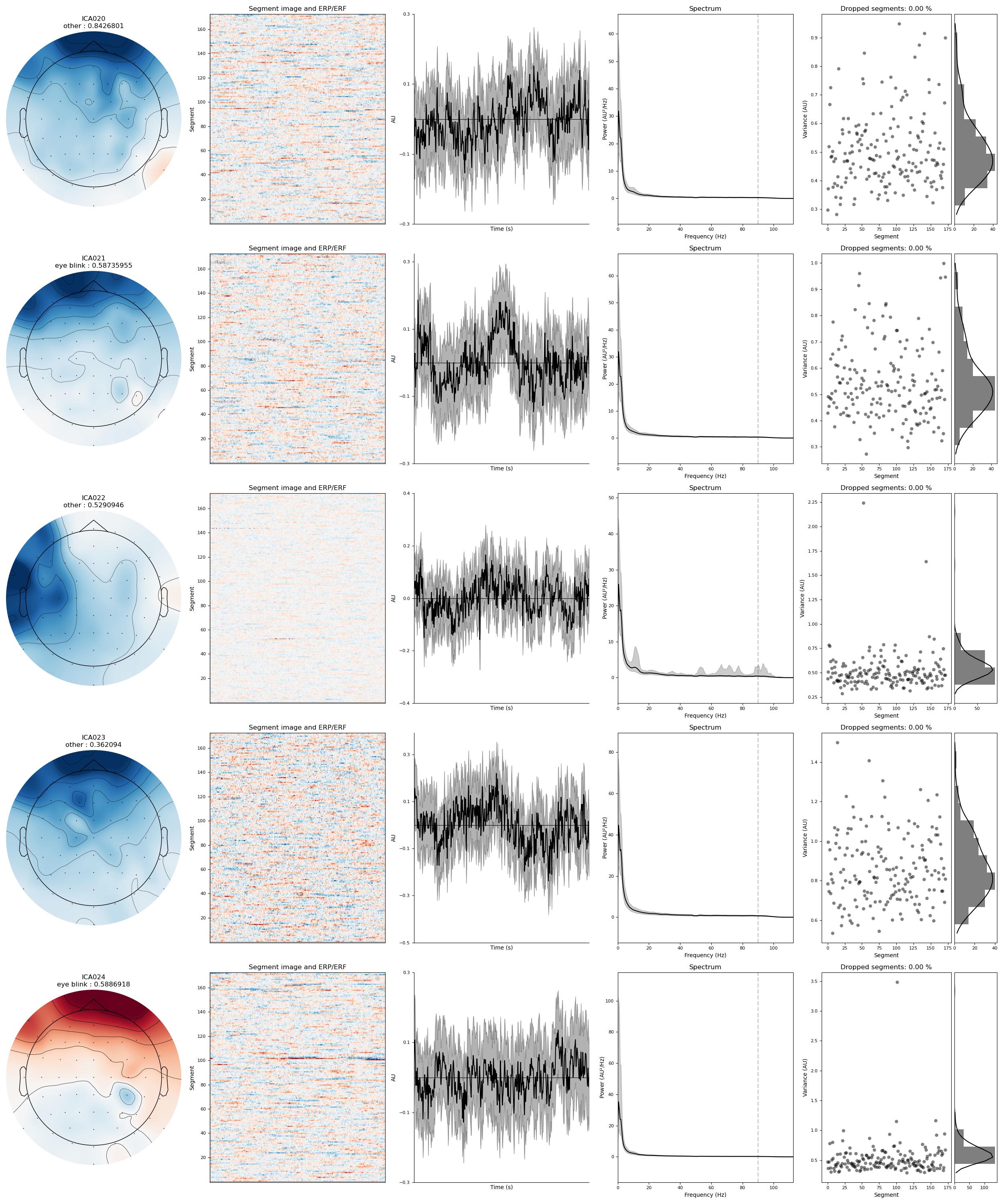

Supplement: Supplementary file 2 [file Data_Sheet_2.zip › component_image/sub16_session2_d1_block1112_4.jpg]
